# Getting back on track to meet global anaemia reduction targets: a Lancet Haematology Commission

**DOI:** 10.1016/S2352-3026(25)00146-2

**Published:** 2025-08-26

**Authors:** Sarah H. Atkinson, Parminder S. Suchdev, Michael Bode, Bianca Carducci, Carla Cerami, Martin N. Mwangi, Sorrel Namaste, Pattanee Winichagoon, Sumie Leung, Agnes M. Mutua, Kelvin Mokaya Abuga, Imelda Angeles-Agdeppa, Robin Blythe, Natalie Carvalho, Ana Cepeda-Lopez, James H. Cross, Saskia de Pee, Erica Di Ruggiero, Jessica Fanzo, Ugo Gentilini, Wanjiku N. Gichohi-Wainaina, Clare Glover-Wright, Filomena Gomes, Sonja Hess, Jacinta Holloway-Brown, Fatou Joof, Crystal Karakochuk, Nicholas J. Kassebaum, Leila Larson, Sachith Mettananda, John Muthii Muriuki, Martha Mwangome, Eric O. Ohuma, Victoria Oliver, Nandita Perumal, Kamija Phiri, Folake Samuel, Sheela Sinharoy, Tinashe Tizifa, Giorgia Valleriani, Kesso Gabrielle van Zutphen-Küffer, Florencia Vasta, Hans Verhoef, Yingying Wang, Kapil Yadav, Zhenyu Yang, Melissa Young, Michael B. Zimmermann, Sant-Rayn Pasricha

**Affiliations:** Kenya Medical Research Institute (KEMRI)-Wellcome Trust Research Programme, Kilifi, Kenya; Department of Paediatrics, University of Oxford, Oxford, UK; Centre for Tropical Medicine and Global Health, University of Oxford, Oxford, UK; Hubert Department of Global Health, Rollins School of Public Health, Atlanta, GA, USA; Department of Pediatrics, Emory University, Atlanta, GA, USA; School of Mathematical Sciences, Queensland University of Technology, Brisbane, Queensland, Australia; Food for Humanity Initiative, Columbia Climate School, Columbia University; The Medical Research Council Unit, The Gambia, London School of Hygiene and Tropical Medicine, London, UK; Healthy Mothers Healthy Babies (HMHB) Consortium, The Micronutrient Forum, Washington DC, USA; Division of Nutrition and Health, Wageningen University, The Netherlands; Data Innovation Alliance, The Micronutrient Forum, Washington, DC, USA; Institute of Nutrition, Mahidol University, Salaya, Nakhon Pathom, Thailand; Walter and Eliza Hall Institute of Medical Research, Parkville, VIC, Australia; Kenya Medical Research Institute (KEMRI)-Wellcome Trust Research Programme, Kilifi, Kenya; Kenya Medical Research Institute (KEMRI)-Wellcome Trust Research Programme, Kilifi, Kenya; Department of Science and Technology, Food and Nutrition Research Institute, Manila, Philippines; Duke-NUS Medical School, Singapore; School of Population and Global Health, The University of Melbourne, Melbourne, Australia; Tecnologico de Monterrey, The Institute for Obesity Research, TecSalud, Department of Nutrition, University of the Incarnate Word, San Antonio, TX, USA; Maternal Adolescent Reproductive & Child Health Centre, London School of Hygiene & Tropical Medicine, London, UK; World Food Programme, Rome, Italy; Dalla Lana School of Public Health, University of Toronto, Canada; Food for Humanity Initiative, Columbia Climate School, Columbia University; Social Protection and Jobs Global Practice, the World Bank; WorldFish, Penang, Malaysia; School of Population and Global Health, The University of Melbourne, Melbourne, Australia; Healthy Mothers Healthy Babies (HMHB) Consortium, The Micronutrient Forum, Washington DC, USA; NOVA Medical School, Universidade NOVA de Lisboa, Lisbon, Portugal; Department of Nutrition, University of California, Davis, USA; School of Computer and Mathematical Sciences, The University of Adelaide, Australia; Center for Global Infectious Disease Research, Seattle Children’s Research Institute, Seattle, Washington, USA; Department of Food, Nutrition, and Health, University of British Columbia, Vancouver, BC, Canada; Institute for Health Metrics and Evaluation, University of Washington, Seattle, Washington, USA; Arnold School of Public Health, University of South Carolina, USA; Department of Paediatrics, Faculty of Medicine, University of Kelaniya, Kelaniya, Sri Lanka; Kenya Medical Research Institute (KEMRI)-Wellcome Trust Research Programme, Kilifi, Kenya; Kenya Medical Research Institute (KEMRI)-Wellcome Trust Research Programme, Kilifi, Kenya; Maternal Adolescent Reproductive & Child Health Centre, London School of Hygiene & Tropical Medicine, London, UK; School of Population and Global Health, The University of Melbourne, Melbourne, Australia; Institute for Health Metrics and Evaluation, University of Washington, Seattle, Washington, USA; Arnold School of Public Health, University of South Carolina, USA; College of Medicine, University of Malawi, Zomba, Blantyre, Malawi; Training and Research Unit of Excellence, Blantyre, Malawi; Nutrition and Dietetics Department, University of Ibadan, Ibadan, Nigeria; Hubert Department of Global Health, Rollins School of Public Health, Atlanta, GA, USA; Training and Research Unit of Excellence, Blantyre, Malawi; Social Protection and Jobs Global Practice, the World Bank; Division of Nutrition and Health, Wageningen University, The Netherlands; Sight and Life, Basel, Switzerland; Global Alliance for Improved Nutrition, Geneva, Switzerland; Division of Nutrition and Health, Wageningen University, The Netherlands; School of Population and Global Health, The University of Melbourne, Melbourne, Australia; All India Institute of Medical Sciences, New Delhi, India; National Institute for Nutrition and Health, Chinese Center for Disease Control and Prevention, China; Hubert Department of Global Health, Rollins School of Public Health, Atlanta, GA, USA; MRC Weatherall Institute of Molecular Medicine, John Radcliffe Hospital, The University of Oxford; Walter and Eliza Hall Institute of Medical Research, Parkville, VIC, Australia; Department of Clinical Haematology, Peter MacCallum Cancer Centre and The Royal Melbourne Hospital, Melbourne, VIC, Australia; School of Population and Global Health, The University of Melbourne, Melbourne, Australia

**Keywords:** Anaemia, global health, epidemiology, interventions, policy and governance, global target-setting

## Abstract

**Global Burden and Data Gaps:**

Many countries lack reliable data on anaemia prevalence, especially for populations beyond young children and women of reproductive age. Few national surveys measure both anaemia and its underlying causes. We call for the creation of a standardised global data repository and the development of a harmonised micronutrient survey platform to collect comprehensive, periodic data. We also recommend better integration of data across sources, including household surveys and other health data sources, and inclusion of haemoglobin assessment in existing survey platforms that already collect venous blood. Continued financial support and coordination of demographic and health surveys are crucial, especially in light of potential reductions in U.S. funding for global data initiatives.

**Anaemia Aetiology and Management:**

The causes of anaemia are multifactorial including iron deficiency, other micronutrient deficiencies, infections, inflammation, blood loss, and inherited blood disorders. We identify critical knowledge gaps in the complex interactions between these risk factors across life stages in different populations.

We recommend targeted research to elucidate underlying mechanisms, improved tools for assessing anaemia determinants, advanced nutritional interventions, and integration of infection control with nutrition programs. Specific areas highlighted for further research include optimising iron dosing and formulations, effective combinations of micronutrients, improving fortification and biofortification strategies, and evaluating non-nutritional interventions such as delayed cord clamping and infection control, and management of heavy menstrual bleeding and post-partum haemorrhage. We also emphasise the need to address environmental factors contributing to anaemia, such as air pollution and climate change.

**Implementation and Governance:**

Effective implementation of anaemia control programs requires tailored, multi-sectorial strategies and ongoing monitoring. Our key recommendations for effective implementation of anaemia reduction programmes are: (1) developing clear governance structures at global, national and sub-national levels to ensure proper oversight and accountability; (2) broadening national nutrition plans to incorporate cross-sector coordination and efficient management of anaemia-related strategies, and (3) placing social equity and fundamental human rights at the centre of anaemia-focused policies and interventions.

**Redefining Future Anaemia Reduction Targets:**

This Commission critically evaluates the process by which the 2030 anaemia targets were set and proposes a more evidence-based, context-specific approach. Key limitations of the current 50% anaemia reduction target are: (1) not clearly accounting for country-specific contexts; (2) focussing on overall prevalence of anaemia rather than anaemia disease burden; and (3) a reduction target for magnitude that was unachievable using available interventions even if maximally deployed.

We proposed a novel target-setting framework based on health economic modelling. This approach incorporates national anaemia prevalence, current intervention coverage and effectiveness, potential scale-up costs, and a range of potential country-specific cost-effectiveness thresholds. This approach aims to balance ambition with achievability while maintaining a unified global vision. Preliminary application of this method suggests a global summary target of 12–22% reduction in anaemia prevalence, significantly lower than the current 50% target, with marked variation in country-specific targets. We advocate for a participatory, iterative target-setting process aligning with local priorities and resources.

**Conclusion:**

Reducing the burden of anaemia requires a comprehensive, multi-sectorial approach that considers its complex aetiology and varied impacts across populations. By adopting the recommendations outlined in this Commission—including improved data systems, more targeted research, integrated programme implementation, and evidence-based target-setting—the global health community can renew momentum toward meaningful anaemia reduction. Achieving progress will require sustained political commitment, increased investment, and coordinated action from governments, international agencies, civil society, and researchers. As the global health agenda evolves beyond the 2030 Sustainable Development Goals, the insights and strategies presented in this Commission offer a roadmap for a more effective, equitable, and sustainable approach to tackling anaemia worldwide.

## Executive Summary

Anaemia, a condition affecting over 1.9 billion people worldwide, disproportionately impacts children, adolescent girls and women. Despite longstanding interventions and guidelines, most countries remain off-track to meet global anaemia reduction targets, and recent cuts in global health funding further threaten progress. This Lancet Haematology Commission aims to reinvigorate efforts to prevent and control anaemia by addressing key gaps in data, evidence, implementation, governance, and target-setting approaches.

## Introduction

Anaemia, defined physiologically as a reduction in haemoglobin concentration below the oxygenation requirements of tissues and clinically as a haemoglobin concentration below a defined threshold,^1^ affects over 1·9 billion people globally.^2^ Anaemia disproportionately impacts children, adolescent girls and women. In 2019, the World Health Organization (WHO) estimated that, globally, 269 million children aged 6–59 months (40%) and over 500 million women aged 15–49 years (30%, including 36% of all pregnant women) were anaemic.^3,4^ According to the 2021 Global Burden of Disease (GBD), anaemia accounts for 5·7% of total years lived with disability (YLDs) worldwide. ^2^ Dietary iron deficiency is the leading cause of anaemia globally across most demographics, including women of reproductive age (WRA) and children.^2^ However, the contribution of other anaemia causes, such as blood loss, may be underestimated in certain regions due to limitations in available reliable data.

**Figure 1:**
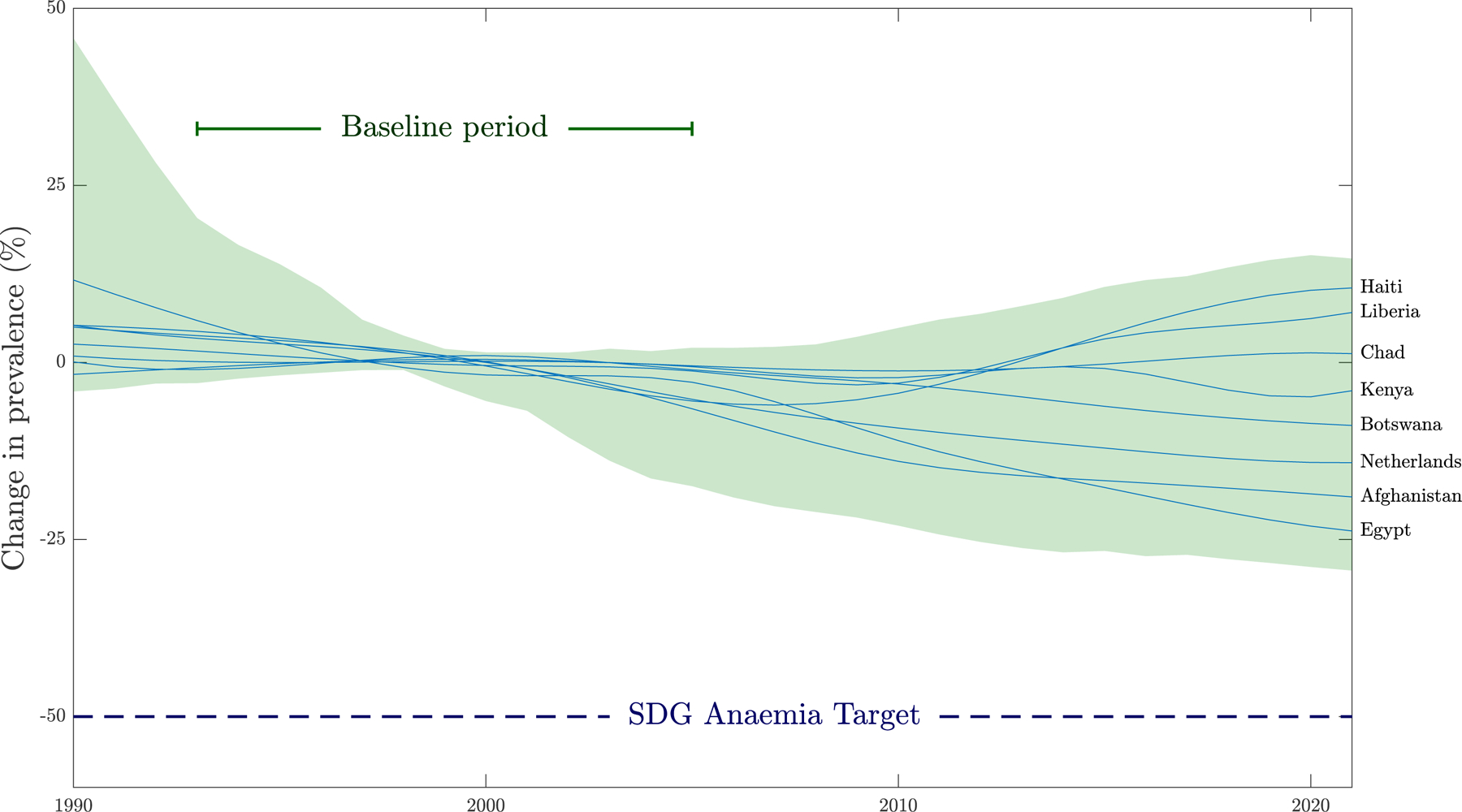
Global Progress towards anaemia targets in women 1990–2021. Shaded area encloses the estimated overall change in the prevalence of anaemia in women of reproductive age, in all countries, as estimated by the Global Burden of Disease (GBD) study.^2^ Individual lines show the specific trajectories of eight select countries. All lines are shown relative to the mean prevalence of anaemia during the baseline period (1993–2005). That is, from the perspective of the Sustainable Development Goals (SDGs) Target Indicator 2·2·3 for anaemia. The horizontal dashed line shows the SDGs and Global Nutrition Targets (GNTs) goal of a 50% reduction in prevalence.

Anaemia prevalence among women is a key indicator for Sustainable Development Goal (SDG) 2 (*Zero Hunger*), Target 2·2 (end all forms of malnutrition by 2030). In response, WHO set a global nutrition target to reduce anaemia in WRA by 50% by 2025, aligning with the SDG Target Indicator 2·2·3. However, despite the availability of interventions and international guidelines addressing anaemia control for decades, most countries remain off-track to meet the 2025 anaemia reduction targets (Figure 1).^5 6^ Alarmingly, anaemia prevalence in women is increasing.^7^ As a result, WHO has extended the target deadline to 2030, yet projections suggest that few, if any, countries will achieve this revised goal (Figure 1).

The global health, nutrition, haematology, and development communities must now critically ask: *Why have we failed to meet the 2025 targets? Why has there been little to no substantive progress in reducing anaemia prevalence? What changes are needed to catalyse transformative progress?* Achieving acceleration in anaemia reduction requires deep reflection on past failures and identification of critical gaps in knowledge across basic, translational, and implementation science. Furthermore, it is time to reconsider the feasibility and value of current targets and explore more sophisticated, evidence-based approaches to setting and achieving anaemia reduction targets beyond 2030.

### Conceptualising the determinants of anaemia

**Figure 2:**
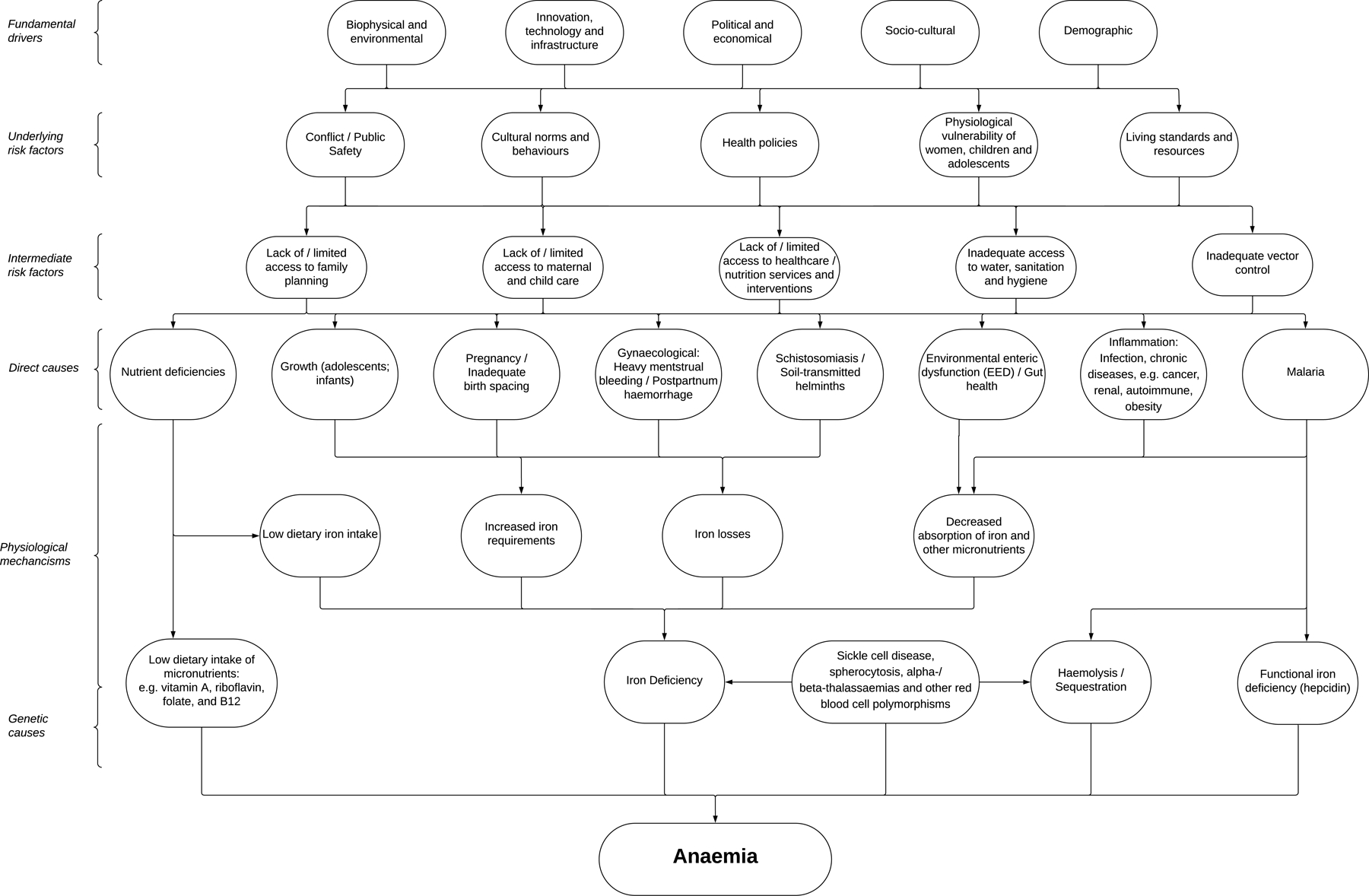
Conceptual framework of anaemia and its drivers

Anaemia can be considered as the endpoint of a cascading set of determinants (Figure 2), beginning with upstream societal drivers such as political, climate and socioeconomic factors. These shape underlying risk factors, including poverty, women’s empowerment, and access to education, which in turn influence intermediate risks like poor water, sanitation, and hygiene (WASH), food insecurity, and limited access to health care, public health programmes, and family planning services. These risk factors contribute to the direct causes of anaemia, such as nutrient deficiencies, blood loss, and infectious or chronic diseases, through distinct physiological mechanisms. For example, inadequate dietary iron intake, malnutrition, inadequate birth spacing, and blood loss due to heavy menstrual bleeding, haemorrhage or soil-transmitted helminth infections lead to iron deficiency (ID). Acute and chronic infections, inflammatory conditions including cancer and autoimmune diseases, may also contribute to anaemia due to impaired iron absorption and recycling. Additionally, recurrent infections may damage the gut and impair iron absorption. Furthermore, inherited red blood cell disorders or carrier states can directly result in anaemia. Multiple mechanisms and underlying conditions may co-exist in the same individual and in a particular community. Clinical testing and analysis of samples from population surveys often fail to fully capture the complexity of the causes of anaemia, and biomarkers of anaemia determinants (e.g. iron indices) can be difficult to interpret in the presence of concurrent inflammation. Recognising this complexity is essential for assessing progress against anaemia targets. While WHO-recommended interventions for anaemia have traditionally emphasised oral iron supplementation and staple food fortification, more recently, its comprehensive framework on anaemia reduction now emphasises multiple-domain solutions.^5^ While effective interventions like iron supplementation, deworming, and malaria control are available for many direct causes of anaemia, new interventions must be developed and tested for other underlying causes. In certain cases, uncovering the underlying mechanisms driving anaemia is crucial for designing appropriately tailored, context-specific interventions, recognising that many common causes, risk factors and implementation challenges can differ significantly between high- and low-/middle-income countries.

### A call to action

This *Lancet Haematology Commission* comes midway through a decade shaped by unprecedented global health and nutrition challenges following the COVID-19 pandemic, rising conflicts, economic shocks affecting food security, and significant changes in the international aid environment - particularly the major shift in U.S. foreign assistance policy in early 2025 (Box 1). It also comes as the world begins to recognise the complex impacts of climate change on health and nutrition.

This Commission aims to reinvigorate progress in preventing and controlling anaemia worldwide by addressing key gaps. First, it examines global estimates of anaemia and its causes (Section 1), characterising global estimates of anaemia and evaluating data quality, availability, and unmet needs in tracking anaemia and its causes. Second, it highlights evidence and knowledge gaps (Section 2), identifying deficiencies in knowledge of anaemia from a public health perspective: biology, epidemiology and public health interventions. Third, it explores implementation and governance (Section 3), proposing an integrated policy and governance framework to improve anaemia solutions over the short, medium and long term at both national and global levels. Finally, it examines global governance and targets (Section 4), reassessing the feasibility of current targets and developing a more sophisticated, data-informed approach to future target-setting.

This global effort integrates perspectives from a multidisciplinary international team of researchers, scientists, clinicians, and policymakers across over 20 countries. By addressing these gaps, this Commission seeks to reshape the global response to anaemia and drive transformative progress towards its reduction.

## Section 1: The global burden of anaemia: uncovering data gaps and challenges.

Robust, translatable and transparent global monitoring of anaemia is crucial for tracking progress, informing resource allocation decisions, and enabling benchmarking among regions and countries. It also helps identify areas for accelerated action in countries with slower progress and provides valuable insights from successful interventions in exemplar countries. Effective global tracking of progress in anaemia reduction requires high-quality, comparable, and reliable anaemia data across different settings.

### Anaemia prevalence: population and geographic distribution

**Figure 3:**
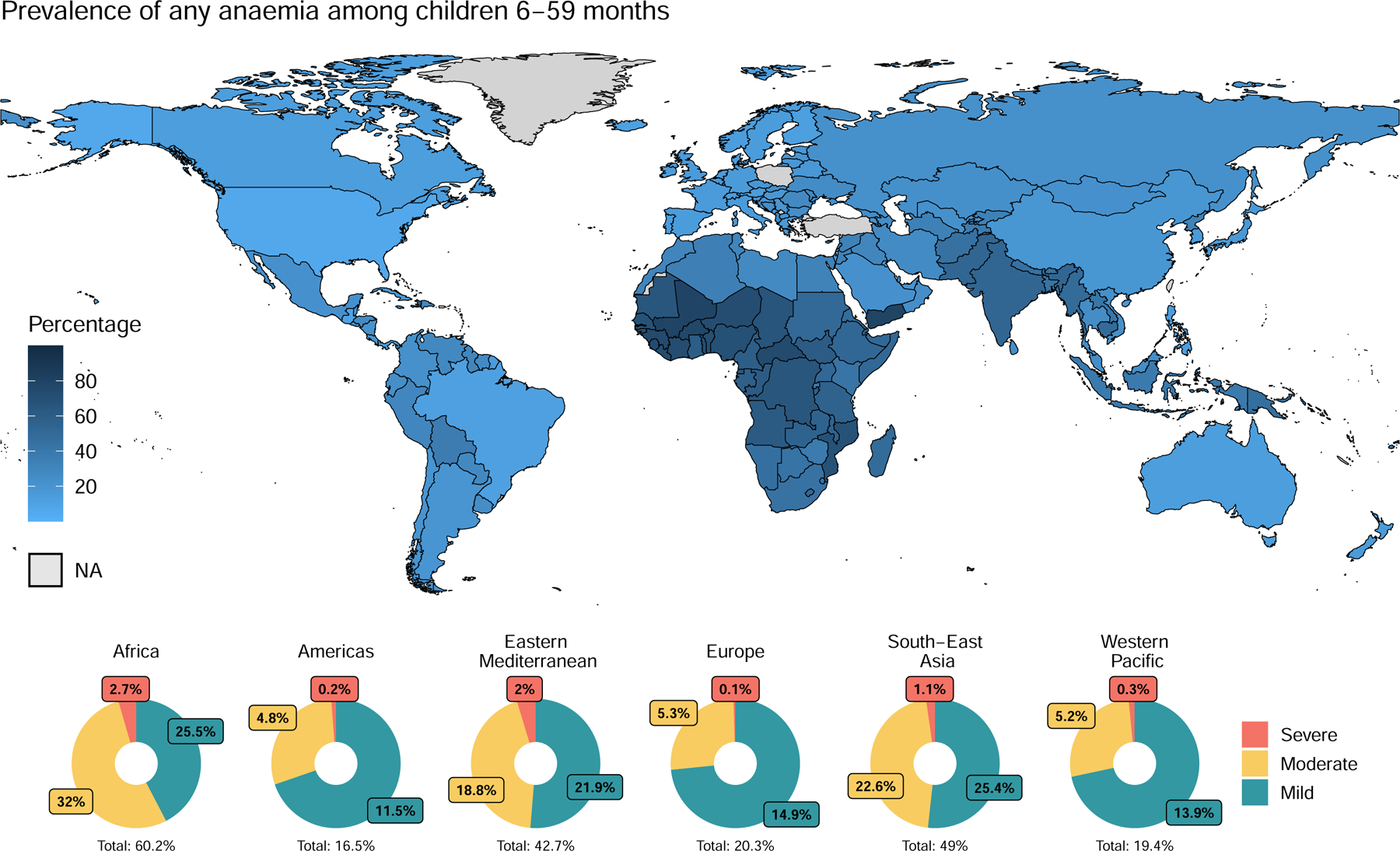

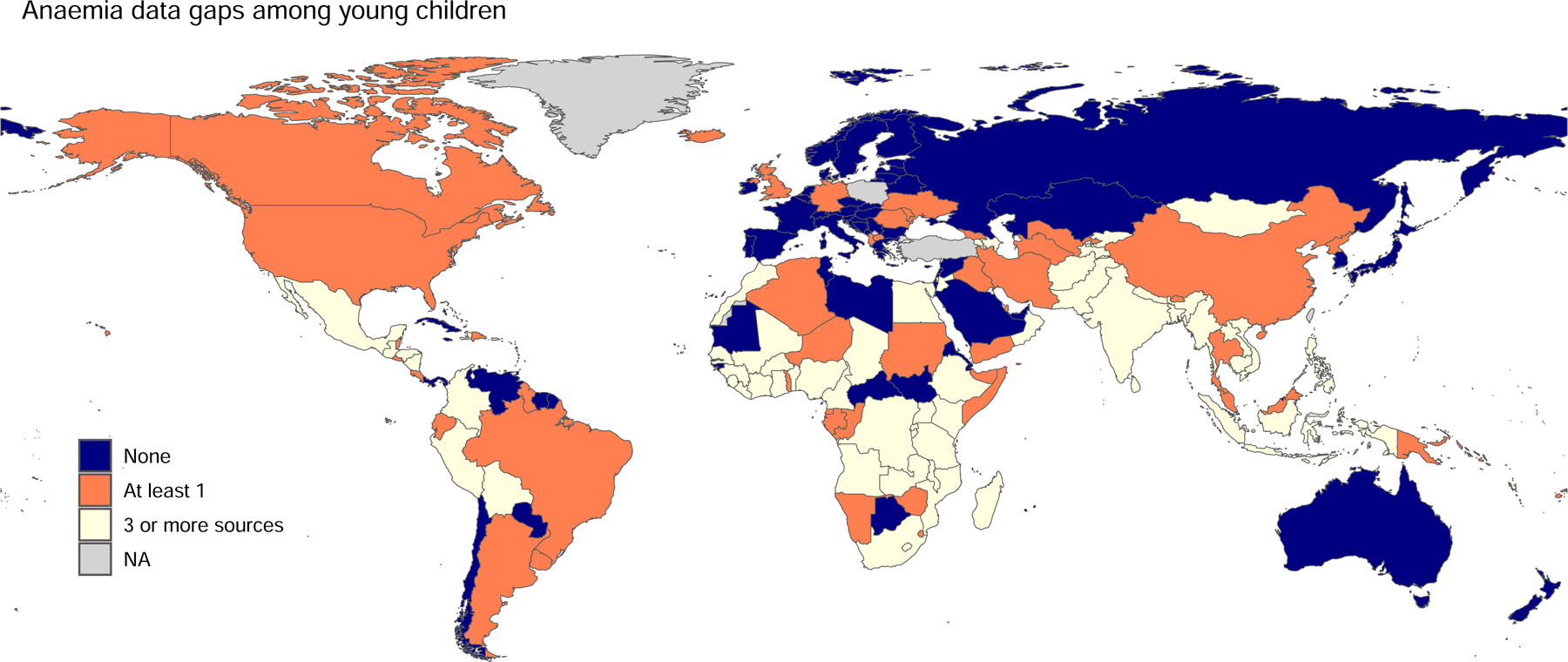

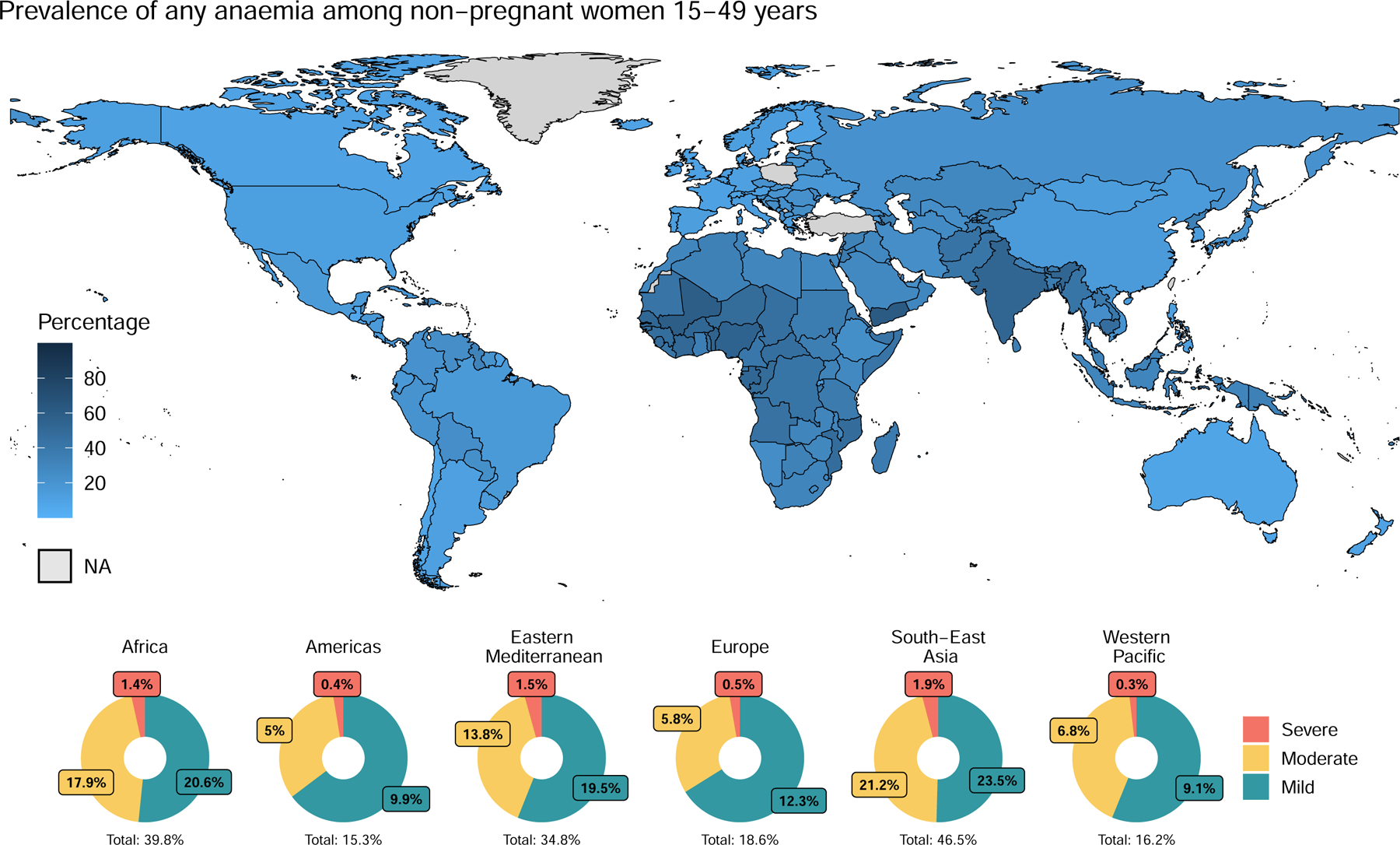

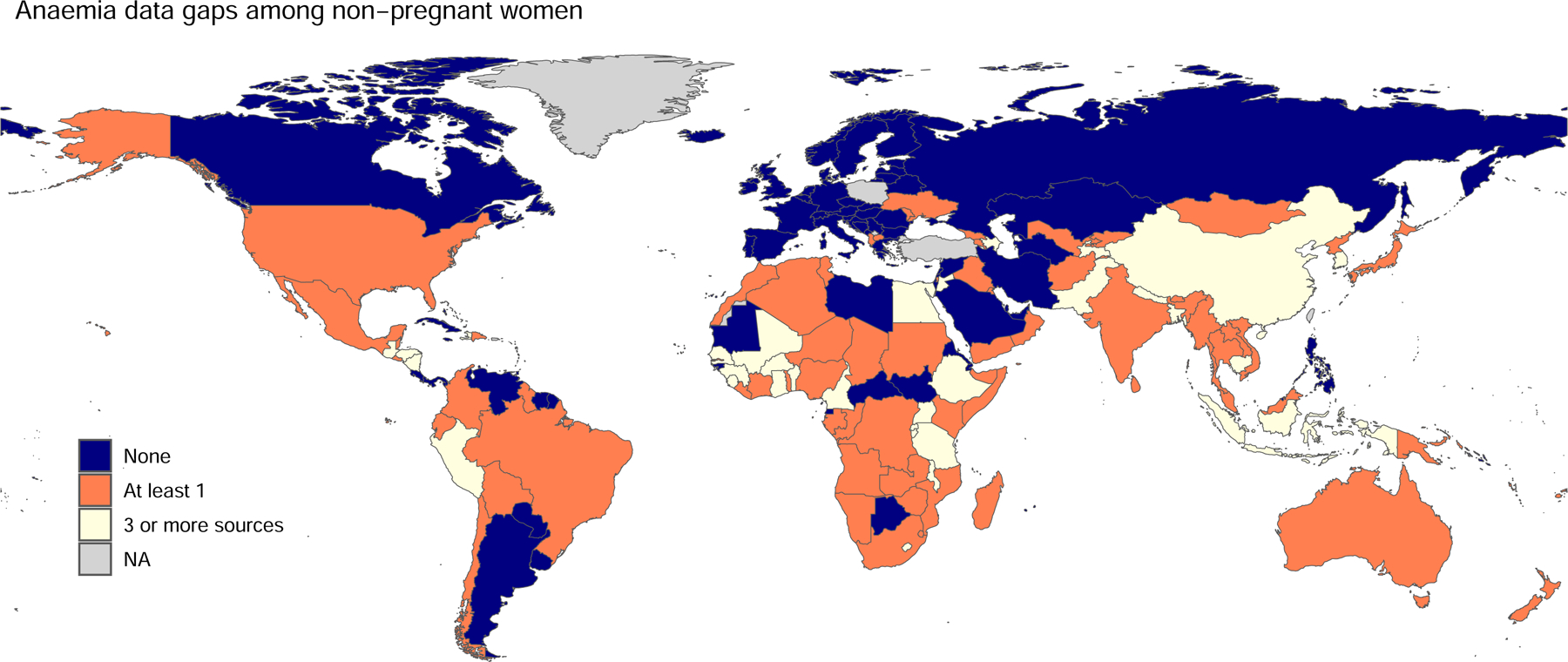

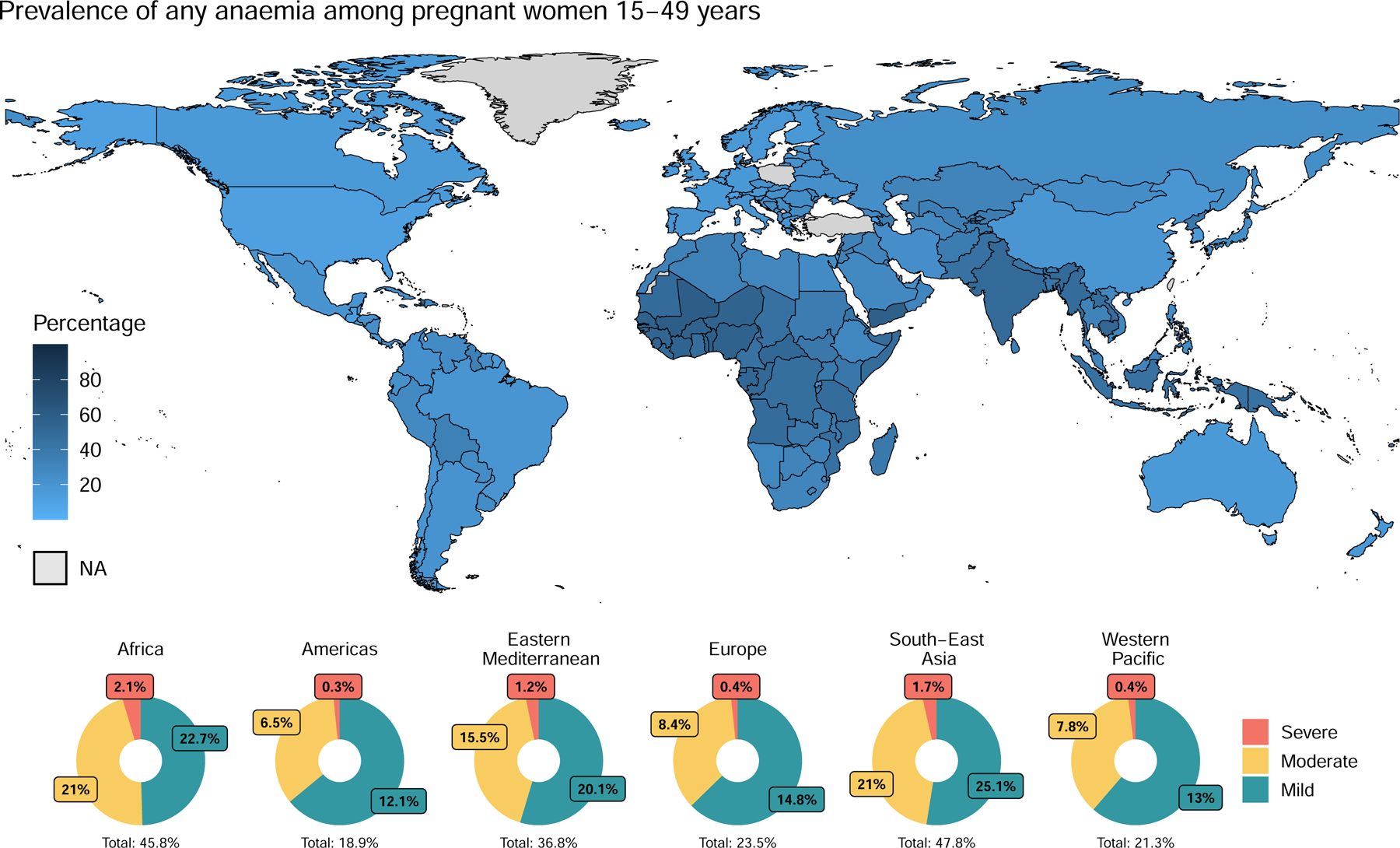

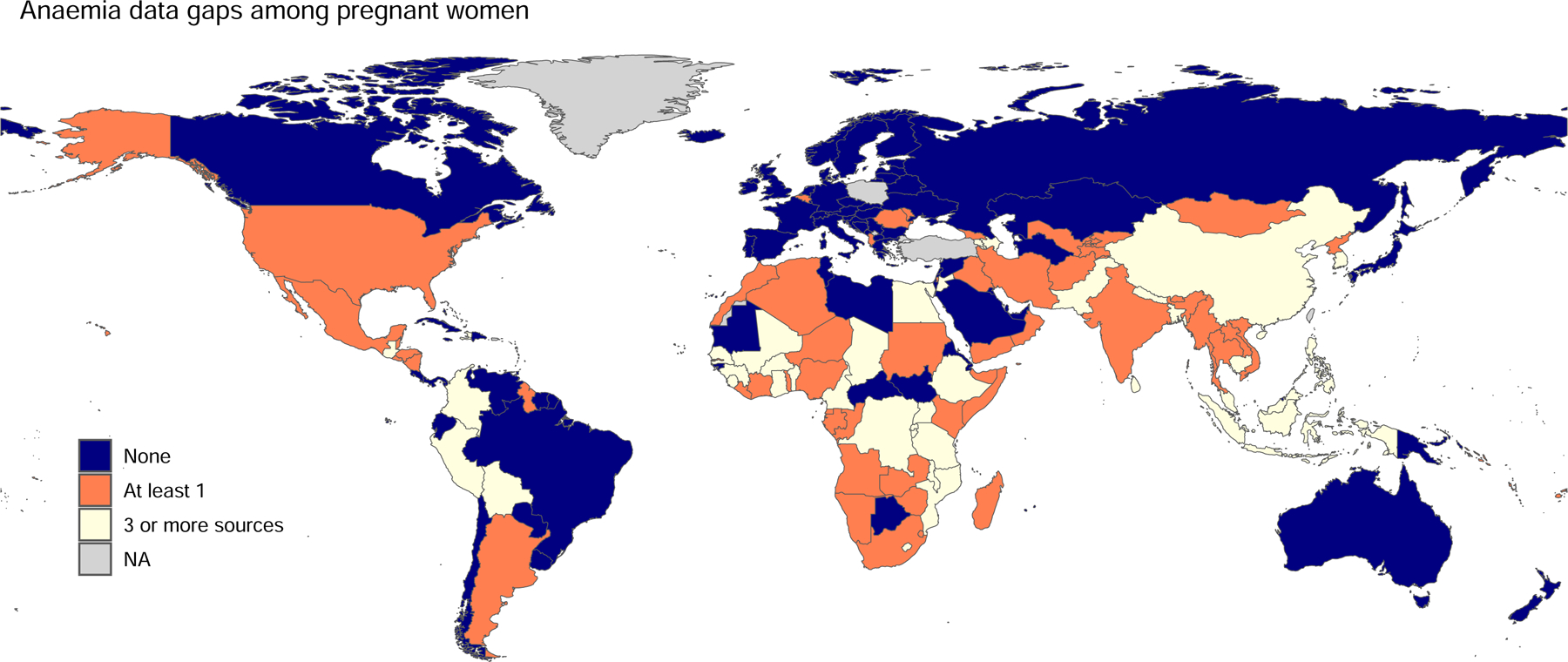
Prevalence of any anaemia in 2019 and data gaps in young children, non-pregnant women, and pregnant women according to WHO Key (for prevalence): 60, 40, 20, NA; Key (for gaps): No anaemia data; At least one source; Three or more sources; For prevalence: Haemoglobin, Hb. Any anaemia is defined as Hb <110 g/L in children aged 6–59 months; Hb <120 g/L in non-pregnant women aged 15–49 years; Hb <110 g/L in pregnant women aged 15–49 years. Data estimates derived from Stevens et al. 2022^3^. Estimates are not yet available based on updated WHO haemoglobin thresholds and adjustments.^12^ For gaps: Data from the Vitamin and Mineral Nutrition Information System (VMNIS) between 2000–2020. Age intervals are defined based on WHO’s VMNIS classification of population groups and do not exactly cover the age ranges of interest: preschool aged children (i.e. young children) 0–75 months, non-pregnant women 12–60 years, and pregnant women 10–54 years. The most common age grouping for each population is 6–59 months for young children (77%), 15–49 years for non-pregnant women (87%), 15–49 years for pregnant women (53%).

Anaemia remains prevalent in low- and middle-income countries (LMICs) (Figure 3; Appendix Figure 1–2 on p. 4–5). Global estimates of anaemia are primarily provided by WHO and the GBD study, both of which rely on national and sub-national population-based surveys. WHO estimates focus on children aged 6–59 months, non-pregnant, and pregnant women aged 15–49 years, while GBD provides estimates across broader age and sex groups. Although their methodologies and population group definitions differ (Appendix Table 1 on p. 6), both data sources show consistent trends over time and across population groups. However, significant data gaps remain. The reliance on modelled extrapolation estimates in data-sparse settings introduces substantial uncertainty, especially for populations beyond young children and women. Specifically, the GBD models incorporate multiple covariates, some of which directly affect haemoglobin levels (e.g. malaria), while others influence anaemia indirectly (e.g. contraceptives) or reflect broader upstream conditions.^8^ Efforts should be geared towards having data that can refine the modelling of estimates, thereby improving the precision of predicted anaemia prevalence and burden.

The global prevalence of anaemia as of 2019 was estimated at 30–40% among children, non-pregnant and pregnant women.^3,4^ GBD data for 2021 show that anaemia prevalence is highest in children under five years, then declines but remains prevalent at 32% in children aged 5–9 years. Anaemia prevalence is also high in adolescent girls with the onset of menarche, affecting 33% of females aged 10–19 years, compared with 18% of males in the same age group.^2,9^ In older persons, anaemia is highly prevalent, rising with advancing age (24% among those 55+, 34% among those 80+).^2,9^ The prevalence pattern by age and sex are reflective of, and aligned with, physiological risks across the lifespan as discussed in Section 2.

Globally, mild and moderate anaemia (categorised by statistically-derived haemoglobin thresholds) each account for approximately half of the total prevalence across most population groups. The pattern differs slightly in older persons (55+), where moderate anaemia constitutes about a third of the total anaemia and the greatest deviation from the pattern is observed in men, for whom moderate anaemia accounts for 15% of the total. Severe anaemia, defined by haemoglobin levels below 70 g/L in pregnant women and young children, and below 80 g/L in other groups, accounts for about 1% of anaemia across all population groups (except for men at 0·21%).^4,9^ The geographical distribution of anaemia prevalence is highly unequal (Figure 3; Appendix Figures 1–2 on p. 4–5). The region of the Americas and some parts of the Western Pacific region experience the lowest prevalence of anaemia (12% in both) while the African and South-East Asia regions experience the highest prevalence (40% and 39%, respectively) based on WHO classification of regions.^9,10^ There are also some geographic variations in the relative contribution of severe, moderate, and mild anaemia to the total anaemia. Severe anaemia is highest in the African region among children of all ages and highest in the South-East Asia region among non-pregnant women and the elderly.

Over the past two decades, global progress in reducing anaemia has been minimal (Figure 1).^2,3^ Between 2000 and 2021, global anaemia prevalence declined by less than three percentage points (from 27.6 to 25.2%) and reductions by region ranged from just one percentage point (Americas) to six percentage points (^9^Africa).

Encouragingly, severe anaemia, more closely linked to adverse health outcomes, has declined across regions and populations.^2,3,11^ Between 2000 and 2021, the global prevalence of severe anaemia reduced from 3·4% to 2·3% in children aged under five years old, and from 1·5% to 1·2% in women aged 15–49 years old, with the largest reductions observed in the African region (from 6·3% to 3·4%) for children and South-East Asia region (from 2·9% to 1·9%) for women.^9^

### Impact of revised anaemia definitions on prevalence estimates

**Figure 4:**
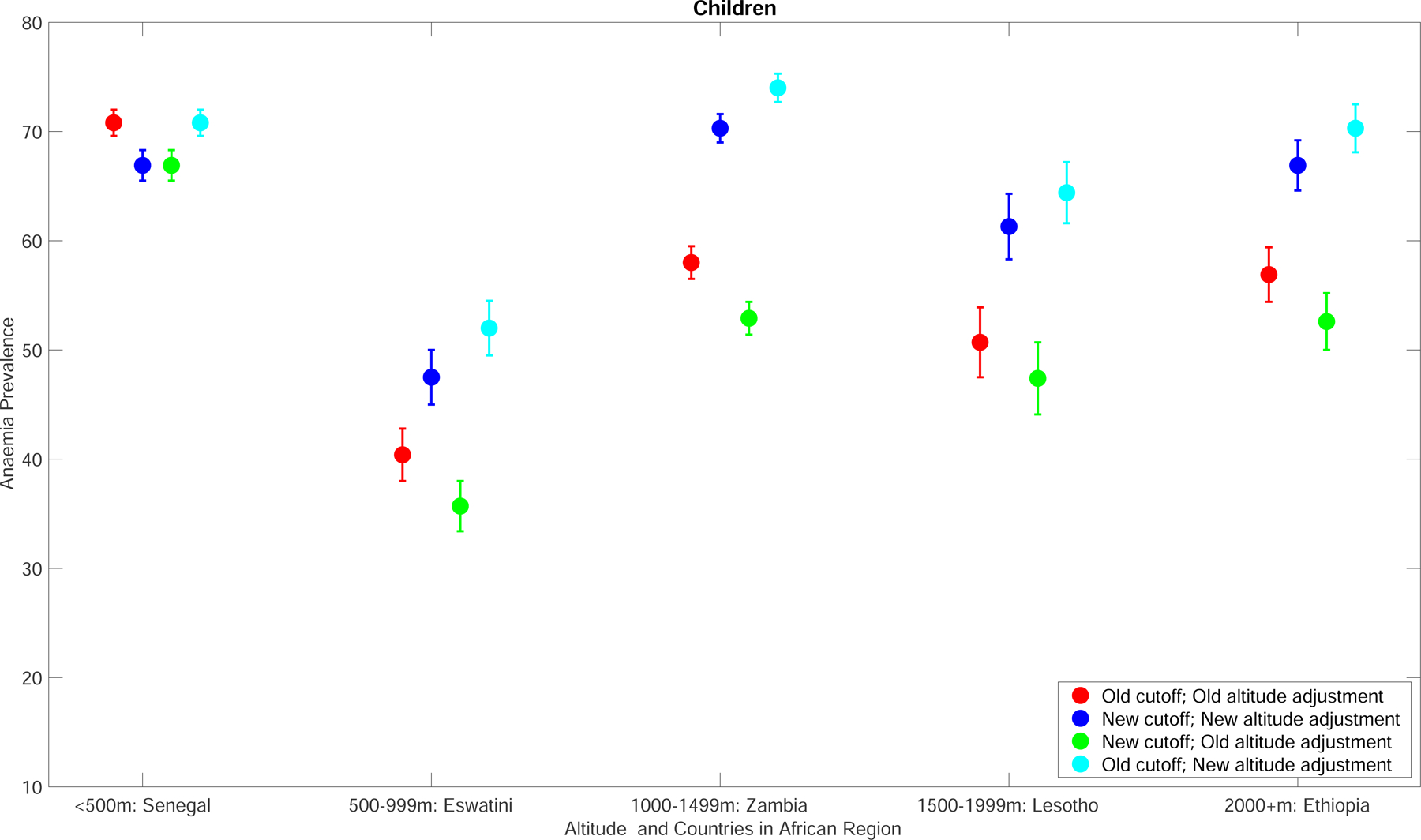

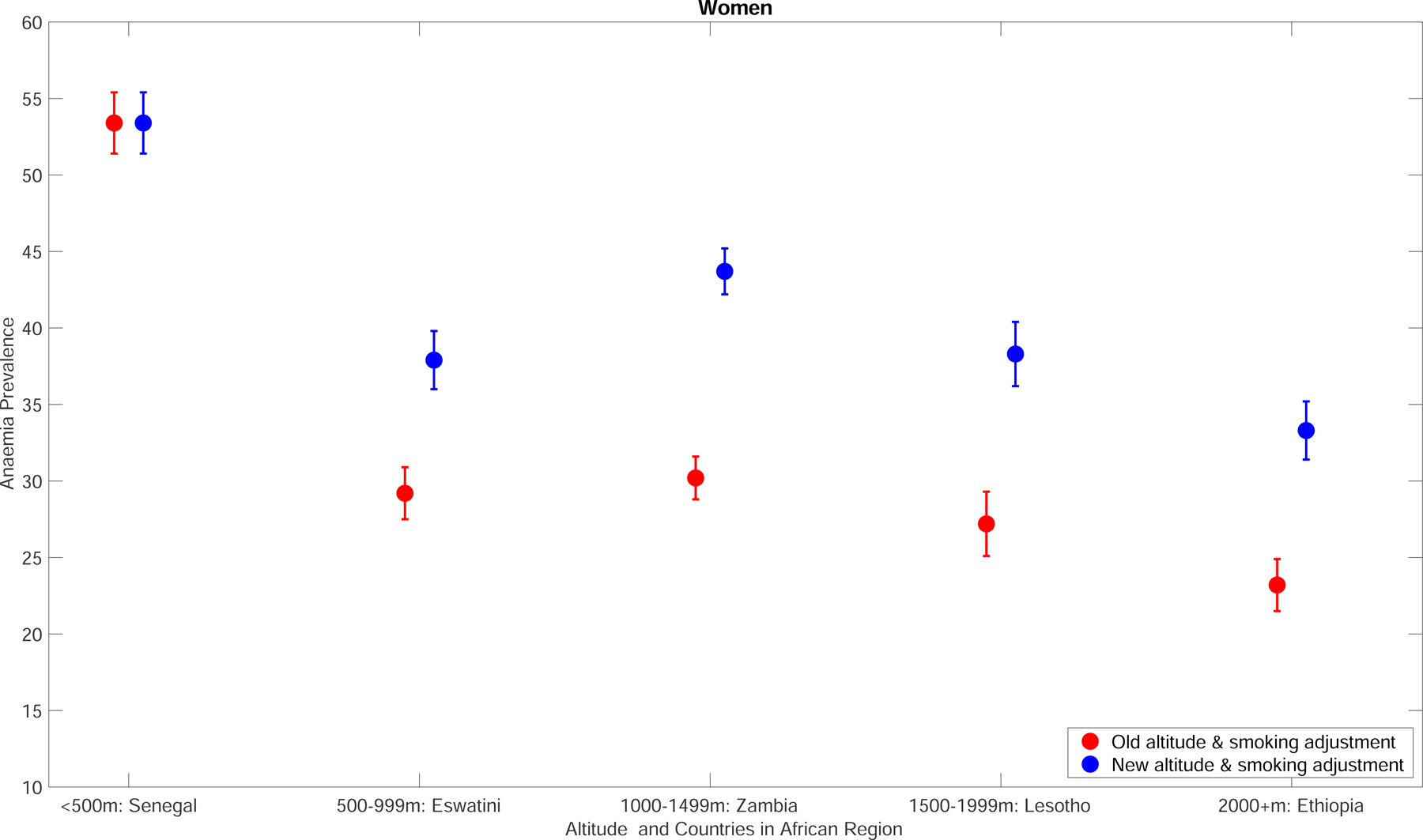
A) Comparison of anaemia prevalence (%) between the new and old cutoffs and altitude adjustment among children aged 6–59 months in the African region. B) Comparison of anaemia prevalence (%) between the old and new altitude and smoking adjustment among non-pregnant women aged 15–49 years in the African region. New cutoff defined as Hb<105 g/L for children aged 6–23 months and Hb<110 g/L for children aged 24–59 months; Old cutoff defined as Hb<110 g/L for children aged 6–59 months. New altitude adjustment defined as Hb adjustment (g/L) = (0·0056384 x elevation in meters) + (0·0000003 x elevation in meterŝ2); Old altitude adjustment defined as Hb adjustment (g/L) = − 0·32 x (0·0032808 x elevation in meters) + 0·22 x (0·0032808 x elevation in meters)^2. New smoking adjustment defined as Hb adjustment (g/L) = (0·4565 x cigarette number per day) + (−0·0078 x cigarette number^2). Old smoking adjustment defined by number of cigarettes per day: <10 (no adjustment), 10–19 (−3 g/L), 20–39 (−5 g/L), 40 or more (−7 g/L), unknown quantity or non-cigarettes smoking (−3 g/L). Sample was limited to the legally recognised resident population. Countries were selected based on the highest percentage of the population residing at different altitudes.^325^ If two countries met the criteria, one was selected randomly. If the same country had the highest percentage of the population at consecutive altitude bins, the altitude bins were combined. Data was restricted to countries with publicly available microdata for children and women.

WHO defines anaemia as haemoglobin values below a certain threshold based on age, sex, and physiologic status (appendix p. 20).^1^ In the revised 2024 WHO guidelines, updated haemoglobin thresholds and adjustments for altitude and smoking were introduced. For children aged 6–23 months, the haemoglobin cutoff was reduced to <105 g/L from the previous <110 g/L, while cutoffs for other population subgroups remained unchanged, except for the inclusion of trimester-specific thresholds for pregnant women and new upper age limits (15–65 years) for adult men and non-pregnant women.^12^ In addition, formulas for adjustments to haemoglobin concentrations for altitude and smoking were also revised (Box 2; Figure 4). Compared with previous adjustments, the updated adjustments to haemoglobin concentration have been increased at lower altitudes and for lighter smokers, resulting in lower haemoglobin levels, whereas adjustments have been reduced at higher altitudes and for heavier smokers, leading to higher haemoglobin values. All these updates represent the most significant change in the definition of anaemia in the past 50 years. Anaemia prevalence estimates presented in this Commission are based on previous thresholds and adjustments since global estimates have not yet been updated using the new definitions. For this Commission, we analysed the impact in selected countries which highlights the potential impact of these changes, emphasising the urgent need for new anaemia models (Box 2; Figure 4).

WHO largely relied on a statistical approach when revising the haemoglobin cutoffs. A limitation of this approach is that the reference population needs to consist of a healthy population. There were insufficient data from studies based in LMICs as well as among certain populations such as older persons when forming the recommendations.^12^ While ongoing work aims to evaluate functional haemoglobin thresholds linked to health outcomes, the current lack of evidence hampers efficient public health decision-making. For instance, it remains unclear if countries should prioritise reducing moderate and severe anaemia, which may have a greater impact on overall health rather than reducing mild anaemia. Research to inform the development of functional severity thresholds is needed, which will also help reclassify the public health significance of anaemia.

### Major data gaps in anaemia, and its causes in population-based surveys.

To assess the scale of the problem of anaemia and progress towards targets, it is crucial that the prevalence of anaemia in target groups (i.e. women, young children and other high-risk groups) is regularly measured at the country level. We sought to determine coverage in the collection and systematic synthesis of anaemia epidemiology data. An analysis of WHO’s Vitamin and Mineral Nutrition Information System (VMNIS), a repository for anaemia and micronutrient data from population-based nationally-representative surveys, revealed that many countries had no national anaemia prevalence data between 2000 and 2020. Even though investments in population-level anaemia assessment have largely focused on young children and women, nearly half of countries lack data within certain population groups (43% for young children, 48% for non-pregnant women, and 50% for pregnant women) (Figure 3).^13^ Data gaps are even more pronounced for other populations, with for example, 99% of countries having no national anaemia data for infants, 76% for school-aged children, 51% for adolescents, 69% for men, and 89% for older persons (Figure 3; Appendix Figures 1–2 on p. 4–5). On average, the European region has the largest gap in national survey data on anaemia within most population groups, while the South-East Asia region has the smallest gap, based on WHO classification of regions and population groups.^10^

Efforts to collect anaemia data are urgently needed for primary school-aged children and adolescents who are still physically, mentally, and cognitively developing and thus vulnerable to potential impacts of anaemia, and who may be accessible through school-based public health interventions. Preventing anaemia is more challenging among older persons, for whom the biological causes of anaemia may differ, and public health solutions remain uncertain. Further consideration for collecting data in this population is needed. For infants, there are challenges with drawing blood and therefore, surveys may not be the best source for such data.

There is also a lack of anaemia data with causes of anaemia measured concurrently. We undertook a mapping exercise of national surveys that collected haemoglobin measurements to assess the frequency with which the following direct causes of anaemia were measured: (1) chronic diseases, (2) infection, (3) inflammation, (4) micronutrient deficiencies; (5) gynaecological and obstetric conditions, and (6) inherited red blood cell disorders (haemoglobinopathies) (Appendix Tables 2 and 3 on p.8–10). No survey measured all these causes of anaemia, and 12% of surveys measured no causes at all. Close to half of the surveys in the African region captured three or more of the six anaemia causes, whereas only 17% of the Western Pacific region surveys included three or more of the six anaemia causes (Appendix Table 4 on p. 11). At least one chronic condition was commonly assessed, with 70% of surveys including data on cancer, gastrointestinal disease, kidney disease, or obesity (Table 1). This high percentage was driven by 69% of surveys that assessed obesity (predominately through height and weight measurements). Data on the other chronic conditions was self-reported and was scarce (5% or less) even in higher-income countries, likely due to reliance on tracking disease from sources that do not include haemoglobin measurements.^14^ Malaria (63%) and HIV (20%) were the only infectious disease biomarkers frequently measured and because of their higher prevalence in the African region most infection-related data comes from this region. Only a small fraction of surveys measured inflammation biomarkers (16%), with little regional variation. Micronutrient status was measured in less than one-third of surveys and the highest frequency of micronutrient assessment was observed in the European region (48%). Iron (27%) and vitamin A (24%) were the most commonly assessed micronutrients and only eight surveys included data on riboflavin all in the European region. Regarding gynaecological and obstetric conditions, four surveys captured self-reported data on heavy menstrual bleeding and few surveys included self-reported data on haemorrhage related to pregnancy or postpartum (8%). Inherited red blood cell disorders were rarely measured (3%); when they were, it was most common in the Eastern Mediterranean region (9%). The lack of data on causes of anaemia from the same source makes it difficult to quantify the contribution of the various causes to anaemia prevalence at the population level.

### Enhancing anaemia data quality and ecosystem.

#### Validity and reliability of anaemia estimates - a critical issue.

The quality of haemoglobin measurement for estimating anaemia prevalence depends on three major domains: pre-analytic factors (e.g. source of blood sample, specimen storage), analytic factors (e.g. choice and quality of analyser), and post-analytic factors (e.g. haemoglobin threshold and adjustments, discussed above).

**Figure 5:**
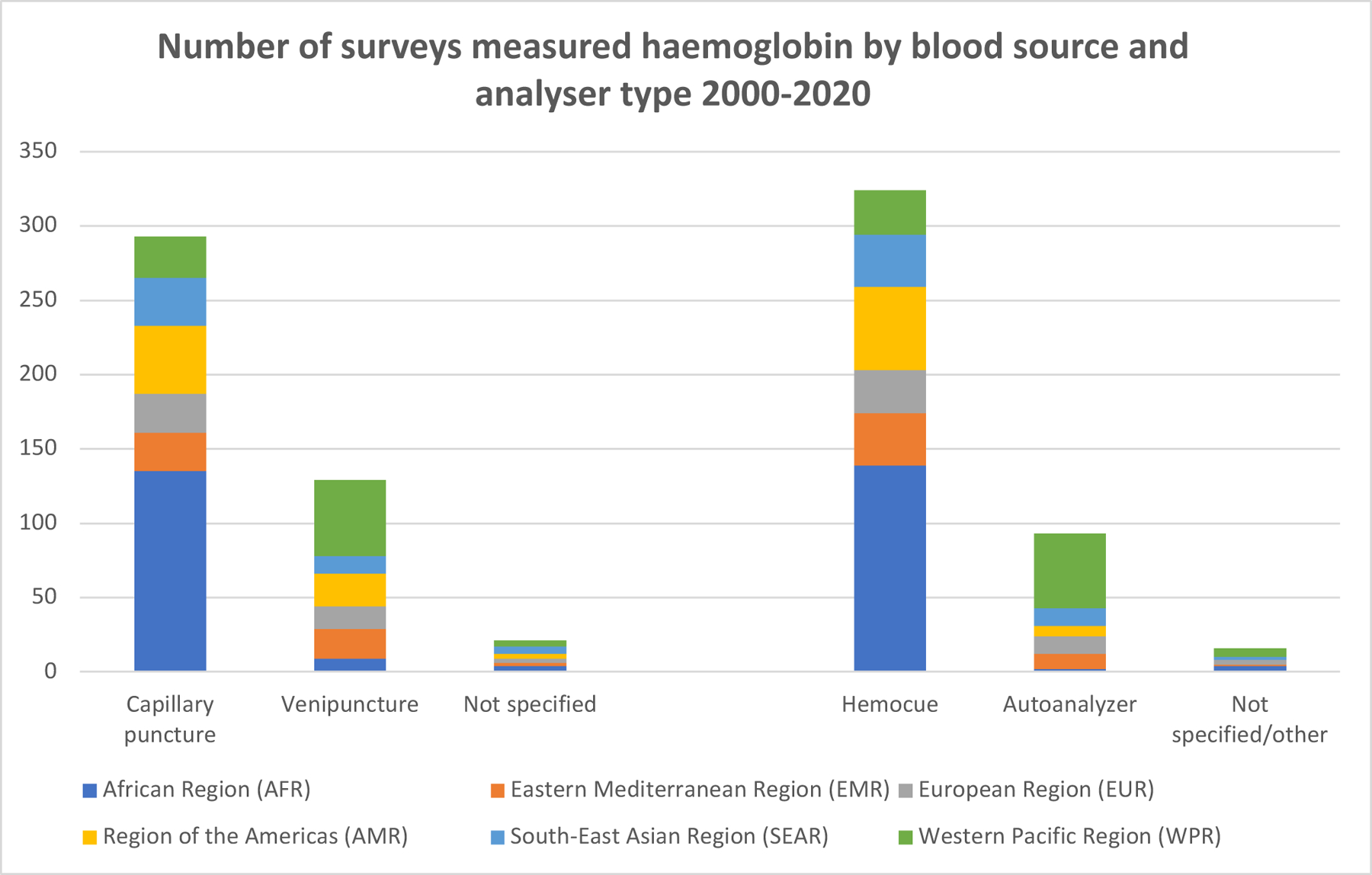
Haemoglobin measurement technique by region. Data from the Vitamin and Mineral Nutrition Information System (VMNIS) between 2000–2020.

Anaemia is typically assessed in population-based surveys by measuring haemoglobin concentration in capillary blood using a point of care (POC) device (Figure 5). Precision and bias issues with single-drop capillary haemoglobin estimates have raised concerns about the quality of global anaemia estimates. ^15^ Observational studies have demonstrated discrepancies between blood sources; for example, Stevens *et al*. found a bias between blood source for children, although not for women, and there was some indication that the HemoCue^®^ 201 performed better than the HemoCue^®^ 301+ device.^16^ In Demographic and Health Surveys (DHS) conducted in Malawi, Rwanda, and Tanzania, where haemoglobin was contemporaneously assessed with both venous and capillary blood in different subsamples, anaemia prevalence in children differed by 12–31% between sources though discrepancies were smaller among women (appendix p. 20).^17^

In response to these concerns, recent method comparison studies were designed to assess haemoglobin concordance between blood sources prospectively. Across these studies, when comparing blood sources measured on a POC device against venous blood with an automated haematology analyser, the 95% limits of agreement (LOA) around the mean were narrower for venous blood (median: ±9·2 g/L, range: ±4·8 to ±16·0 g/L) than for pooled capillary blood (median: ±12·1 g/L, range: ±6·6 to ±28·6 g/L) and single-drop capillary blood (median: ±15·7 g/L, range: ±9·4 to ±36·8 g/L) (appendix p. 20).^18,19^However, there is a large overlap in the ranges for the 95% LOA around the mean across all blood sources, with capillary blood even performing better than venous blood in some study sites. In a review of these and other studies, imprecision of the haemoglobin measurement on an automated haematology analyser compared with a POC device was consistently lower than the level of imprecision observed due to the blood source.^20^

For these reasons, the 2024 WHO guidelines recommended, where feasible, measuring haemoglobin using venous blood with an automated haematology analyser coupled with high-quality control measures.^12^ Standards have not been established on what constitutes an acceptable level of measurement uncertainty in haemoglobin measurements in the context of population surveys. Defining acceptable levels of uncertainty is urgently needed and should be driven by the intended uses of the data. Surveys are primarily used to inform population-level health and public health interventions and policies, rather than focusing on diagnostic accuracy at the individual level. Thus, an important criterion to consider is the impact the haemoglobin assessment technique has on data quality and the correct classification of anaemia as a public health problem at the population level.

Transitioning to the preferred method of measuring haemoglobin using venous blood is feasible, as demonstrated by its use in many surveys, despite the more complex logistics and increased resources required.^21^ However, shifting to venous blood calls for careful planning and monitoring of the factors that can impair data quality. In particular, there is the potential for lower response rates due to participants declining blood draws, especially for young children, over concerns about safety or discomfort.^22^ In addition, drawing a substantial volume of blood for a single analyte (haemoglobin) or just a few analytes (that could have been measured on capillary blood) may raise concerns regarding the acceptability of the survey in the community that could outweigh the benefits of improved data quality. While adding additional analytes that can be measured with the venous blood would greatly increase the benefit of performing venous blood draws, this necessitates laboratory-based testing, which significantly alters survey operations and costs, especially when the survey primarily collects questionnaire-based data. In the future, non-invasive haemoglobin measurement technologies could enable cheap large-scale population measurement, but the development of such technologies is still in the early stages, and currently available platforms do not yet meet the desired level of accuracy and precision for haemoglobin assessment in population-based surveys (appendix p. 20–21).^19^

#### Analytical method considerations for haemoglobin measurement

A well-maintained automated haematology analyser supported by high-quality control systems is considered more accurate in measuring haemoglobin concentration compared with a POC device.^23^ These instruments are generally available in clinical haematology laboratories but have been seldom used in surveys. Their use poses many logistical and resource constraints (Figure 5).^24^ At the pre-analytical phase, whole blood has to be maintained between 4°C and 8°C from the time of collection to analysis, which may be more challenging than freezing samples, as is commonly done for serum analytes including micronutrients. A real-world example that highlights this issue is the 22% sample loss from the difficulties of maintaining storage temperatures in a study that mimicked a survey-like context.^18^ Laboratories must be located in-country that meet quality management standards and have space to store refrigerated samples to allow for testing as soon as possible and within the seven-day limit.

In contrast, POC devices have many advantages – they are simple to use, portable, do not require a cold chain for the sample, and survey participants can receive their results immediately.^25^ Both automated analysers and POC devices require analysis of regular internal quality control samples to ensure optimal performance and should participate in external quality control. Specialised technicians are required to repair and calibrate instruments. Automated analysers require an ongoing supply of liquid reagents to perform analysis, whereas POC devices undertake the analysis within a cuvette. Since the blood source may contribute more to imprecision than analytical factors (i.e. type of instrument) (appendix p. 21),^20^ the potential benefit of automated haematology analysers may be outweighed by their increased field burden and risk of greater measurement error compared with POC devices.

Nevertheless, systems and infrastructure that allow automated haematology analysers to become standard practice in all surveys are needed given that this is a preferable method to POC devices.^12^ Beyond accuracy, a potential advantage is that automated haematology analysers also measure red blood cell (RBC) indices including mean corpuscular volume, mean corpuscular haemoglobin, and red cell distribution width; in some cases, they can also measure reticulocyte count and even reticulocyte haemoglobin concentration (a useful index of iron status). RBC indices can point to possible causes and/or types of anaemia.^26^ Nonetheless, while these indices aid in differential diagnosis of the cause of anaemia at the individual level, the interpretation of these indices at the population level, when these values have been cumulatively pooled across a large sample, is unclear. This validation research needs to be carried out even before we assess whether the additional information provided by these indices would incur a lower cost and field burden beyond measuring haemoglobin in conjunction with micronutrient biomarkers.

As a way forward, models for analysing haemoglobin using automated haematology analysers in population surveys have been used in some contexts (Appendix Box 1 on p. 12) and we should be working to develop data collection infrastructure that makes this feasible across contexts. Research should also be undertaken on the application of automated haematology analysers in resource-limited environments to inform the development of guidelines for their use in population-based surveys. This could involve identifying the minimum conditions required, such as cold chain parameters and validating storage periods, as well as quantifying differences in measurement error between using an automated haematology analyser and a POC device. Although the technology is not well advanced, development of portable analysers that are capable of withstanding survey field conditions would be preferable in the long term.

#### Using anaemia data to track global anaemia reduction progress

Anaemia data in WRA 15–49 years are critical for tracking the SDG 2 and the World Health Assembly (WHA) 2025 target. Most available haemoglobin data have been measured in capillary blood on a POC device (Figure 5). In the African region, where the burden of anaemia is greatest, 94% of surveys used capillary blood and 99% of surveys used a POC device. Excluding large quantities of historical data because haemoglobin was measured in capillary blood or on a POC device will cause the loss of substantial amounts of baseline information and introduce selection bias into the overall estimates. This is particularly relevant since measurement issues are likely to affect estimates for women less than for children, and the SDG indicator and WHA target specifically pertain to women. Given that data collection procedures from the past 20 years are unmodifiable, and it may take time to find suitable platforms for measuring anaemia with the preferred methodology, these existing data should still be utilised, ideally by applying adjustments or using modelling approaches.

#### Establishing new or strengthening existing survey platforms

Substantial investments in information systems are needed to increase the availability of anaemia and cause-specific data. The urgency is heightened by countries’ reliance on anaemia data from The Demographic and Health Surveys (DHS) Programme through DHS and Malaria Indicator Surveys (MIS) (comprising 34% of the data globally and 69% for the African region). As of 2024, The DHS Program no longer includes haemoglobin measurement as a standard in MIS surveys. In DHS surveys, it now recommends measuring haemoglobin with venous blood, and only if it is not the sole biomarker being assessed. This change may impact whether countries continue to include haemoglobin measurement in their DHS surveys.

This evolving landscape presents an opportunity to modify existing data collection platforms and develop new ones. Selecting the right survey platform requires careful consideration of its advantages and disadvantages and the most suitable platform will depend on the context (Appendix Table 5 on p.13–14). Presently, standalone micronutrient surveys are the most common approach for collecting data on anaemia and its causes, but their infrequent occurrence has prompted interest in leveraging alternative approaches.^27^ For example, in Malawi, data collection of micronutrient status and other causes of anaemia was added to an existing DHS survey (‘piggyback’ approach), where the micronutrient teams followed the main DHS survey teams.^28^ This approach provided comprehensive data but faced coordination challenges between the agencies responsible for the different survey components and between the field teams. In 2024, Malawi used a fully integrated approach to address these previous challenges, with micronutrient team members working alongside those collecting standard DHS data; however, this approach incurs significantly higher costs.^29^ In contrast, Guatemala has developed a high-quality, and timely nutrition surveillance system that continuously collects national survey data, and Uganda has integrated a nutrition module into an existing multitopic panel survey, with different biomarkers included in each cycle (appendix p. 21).^30^

In addition to household surveys, there are other sources of data on the causes of anaemia. Better linkage is needed between these sources and anaemia data, such as cancer registries and national tuberculosis notification systems. The integration of these data within a centralised monitoring system, e.g. led by WHO can help address this need. In line with a WHO resolution,^31^ the public release of country estimates involves consultation with WHO Member States, allowing them to review methods, data sources and provide updated data. During this consultation process, WHO can resist political pressures and publish its own comparable estimates alongside country-reported numbers. Such discrepancies increasingly drive efforts by countries, often in collaboration with WHO, to improve data availability and quality for the estimates.^32^

Since anaemia is an indicator of multiple health determinants within populations (Figure 2), combining measurement of haemoglobin with collection of data on anaemia aetiology can capture some of this complexity.^33^ However, this approach requires significantly more survey infrastructure, and haemoglobin data is still valuable alone. In Box 3, we highlight the existing limitations to the data ecosystem and propose key actions needed to strengthen the anaemia data landscape.

#### Using data on anaemia causes to develop context-specific solutions

Anaemia is caused by the complex interplay of fundamental drivers, intermediate risk factors, and direct causes (Figure 2). Untangling the complex interplay between anaemia, its causes, its risk factors and their clinical presentations on the wide range of social and economic consequences of anaemia is a major challenge.

The GBD study attempts to attribute the relevant contribution of different causes to anaemia, but with limited data on the determinants of anaemia it relies on proxy indicators, requiring assumptions and complex statistical modelling (appendix p. 21).^2^ The contribution of the different causes of anaemia also varies by severity of anaemia, age group, gender, and geographical region. Further, many of these causes occur concurrently and are also interconnected as they share the same risk factors. For example, the burden of infections with malaria, soil-transmitted helminths, including hookworm, and schistosomiasis is higher in tropical and sub-tropical regions of Sub-Saharan Africa. Low socioeconomic status exacerbates the risk of infection via poor living conditions, poor dietary quality, and limited access to health services. An additional complexity is the non-specific role of anaemia of inflammation, which is caused not only by chronic diseases and infections but also by “inflammaging” or chronic inflammation caused by early life adversity (appendix p. 21).^34^

Because of these complex relationships, it is challenging to adequately account for each risk factor and disentangle the contribution of each cause to the anaemia burden in different populations. To understand and prevent anaemia the planning of new multi-disciplinary surveys, and choice of specific indicators should be guided by the context-specific causes and risk factors for anaemia (appendix p. 21).^23,33^Additionally, further exploration is needed on how attributable fraction and other methodological approaches can be used to better understand the determinants of anaemia in different contexts to inform decision-making at the national and sub-population levels.

## Section 2 – Anaemia control must consider its complex multifactorial aetiology.

As described earlier, anaemia arises from a range of direct and indirect causes through distinct physiological mechanisms. This section revisits the biology of anaemia in a global health context, examining both nutritional and non-nutritional causes. We identify gaps in understanding of causes and their interconnections, prioritise areas for further research, and interventions to address anaemia.

### Nutritional factors in anaemia

Nutritional anaemia occurs when deficiencies in essential nutrients required for erythropoiesis lead to reduced haemoglobin levels. These deficiencies may result from inadequate dietary intake, poor absorption, nutrient losses or increased physiological demand during periods of rapid growth, pregnancy, or illness.

#### Physiological iron needs by life stages

Iron deficiency (ID) is the leading cause of anaemia globally, accounting for 10%–60% of anaemia cases, depending on the population and context (appendix p. 22).^35^ ID may arise from insufficient iron stores (absolute ID) or limited mobilisation of iron despite adequate stores (functional ID), both impairing haemoglobin production (appendix p. 22).^36^ Iron is an essential component of haemoglobin and myoglobin, proteins that are involved in oxygen carriage and storage. Iron supplies are achieved via nutritional intake from the diet, and from iron recycled from senescent red blood cells.^37^ In a normal physiological state, iron needs can be met by adequate bioavailable iron intake to offset basal iron loss from skin, hair, urine, faeces, and sweat. The most reliable data on basal iron losses, based on a single study in adult men with normal iron status, estimate losses at 14 µg/kg (~1–2 mg/day) and are typically extrapolated to other age groups by body weight.^38^ The body has homeostatic mechanisms to regulate iron balance to avoid iron deficiency and overload. Regulation of systemic iron metabolism is primarily driven by the liver-derived hormone hepcidin and the iron exporter ferroportin, which together control iron absorption, utilisation, recycling, and loss. ^36^

Recommended Dietary Allowance (RDA) for a nutrient, such as iron, is the average daily nutrient intake that is sufficient to support known nutritional requirements in nearly all (97%–98%) healthy individuals at a specific age, sex, life stage or physiological state.^39^ RDA values for iron are estimated based on assumed absorption rates from mixed (both animal and plant sources) diets: 25% for pregnant women, 10% for infants (7–12 months) and 18% for adolescents, adult men, postmenopausal women and the elderly. ^40^ During pregnancy, iron absorption rises from 1 mg/day to 7 mg/day by the third trimester to support red blood cell mass expansion and foetal development (appendix p. 22).^41^ To meet this high iron demand, the RDA reaches its peak at 27 mg/day during pregnancy.^40^ In healthy pregnancies, approximately 270 mg of iron is cumulatively transferred to the foetus, creating a critical iron reservoir for the first six months of life.^41^ However, placental iron transfer may be adversely affected by maternal ID, inflammation, and other underlying health conditions, leading to increased risk of anaemia in the infant (appendix p. 22).^41^ While dietary iron requirements are lower during lactation due to amenorrhea, this is a critical stage for maternal iron store recovery, given the high iron costs of pregnancy and postpartum blood loss.^41^ Pregnant adolescents face compounded iron needs due to both ongoing growth and the increased demands of pregnancy.^42^

**Figure 6:**
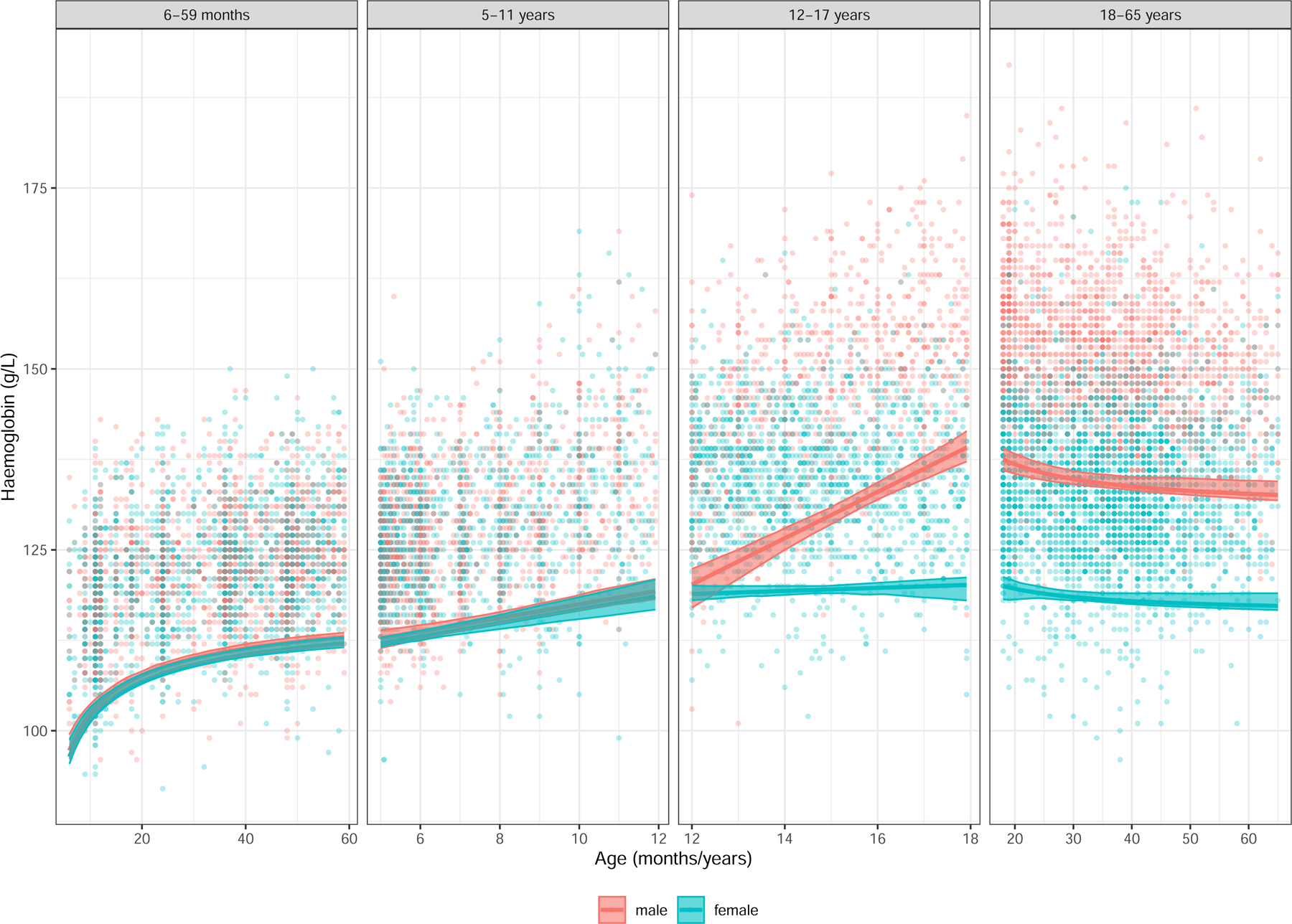
Continuous 5th centile haemoglobin thresholds in males (red) and females (blue) across the life course (6 months to 65 years) Using datasets from the Australian Health Survey-AHS (adults aged 18 to 65 years in Australia), China Health and Nutrition Survey-CHNS (adults aged 18–65 years in China) Applied Research Group for Kids-TARGet Kids! (children aged 6 months to 11 years in Canada), Benefits and Risks of Iron interventions in Children-BRISC (children aged 11 months in Bangladesh), Encuesta Nacional de Salud y Nutrición-ENSANUT (children aged 6 to 59 months in Ecuador), Generation R study (pregnant women aged 18 to 45 years in the Netherlands), Health Survey for England-HSE (Adults aged 18 to 65 years in England) and National Health and Nutrition Examination Survey-NHANES (children and adults aged 6 months to 65 years in USA). The pooled continuous centiles and confidence intervals across data sources were estimated without accounting for the complex survey and weighting. Figure reproduced with permission from Bratt et al. (2024).^1^

Healthy full-term infants are born with high haemoglobin levels due to a predominance of high oxygen affinity foetal haemoglobin (HbF) which provides them with a substantial iron reserve. However, preterm and small-for-gestational-age infants have lower iron stores at birth due to reduced gestational iron accretion, making them more vulnerable to early-onset ID.^43^ Further research is needed to optimise strategies for preventing anaemia in these high-risk infants. After birth, haemoglobin concentrations gradually decline, typically reaching their lowest point between 6–12 weeks of age, due to a decrease in erythropoiesis in response to higher oxygen levels and as HbF is replaced by production of adult haemoglobin (appendix p. 22).^44^ By six months, iron stores acquired at birth are typically depleted, necessitating dietary intake to meet high iron demands.^40^ Between 7–12 months of age, the RDA for iron intake for infants is high at 11 mg.^40^ Haemoglobin levels gradually increase at a steady rate during childhood, continuing through at least puberty, with a greater rise in males due to higher levels of testosterone (Figure 6; appendix p. 22).^1^

During puberty, iron needs for both girls and boys increase due to rapid growth and expansion of the erythroid mass. ^45^ Adolescent boys are recommended to consume 11 mg of dietary iron daily. ^40^ For girls, the onset of menarche further raises iron demand to compensate for menstrual blood loss throughout their reproductive years. ^40^ To meet this increased iron demand, the recommended daily iron allowance is 15 mg for adolescent girls and 18 mg for premenopausal women.^40^ In adult men, postmenopausal women and the elderly, the recommended daily iron allowance is 8 mg daily to cover basal losses.^40^ Blood loss due to chronic gastrointestinal bleeding, can further increase iron demands (appendix p. 22).^40^

#### Role of micronutrient deficiencies in the development of anaemia

In socioeconomically vulnerable populations, coexisting micronutrient deficiencies often interact to exacerbate anaemia (appendix p. 22).^46^ While ID typically causes microcytosis and vitamin B12 and folate deficiencies cause macrocytosis, the co-occurrence of these deficiencies may obscure clinical features and complicate diagnosis, leading to underestimation of the prevalence of iron, vitamin B12 and folate deficiencies. ^47^ The mechanisms through which micronutrient deficiencies contribute to anaemia are summarised in Table 2. Further discovery and population health data are needed to confirm the mechanisms by which some of these deficiencies cause anaemia. The potential for multiple interacting nutrient deficiencies to cause anaemia highlights the need to assess nutritional biomarkers more comprehensively when investigating causes of anaemia at the population level.

#### Dietary determinants of iron deficiency and anaemia

##### a) Dietary iron adequacy and bioavailability

The adequacy of dietary iron is influenced by both its quantity and bioavailability. Insufficient intake or poor absorption of iron can lead to iron deficiency anaemia (IDA). Iron absorption and utilisation depend on its form and the presence of other dietary components that influence absorption. Dietary iron exists in two forms: haem iron, predominantly found in animal products like meat, poultry, and fish; and non-haem iron, abundant in plant-based foods like beans, lentils, spinach, and fortified cereals.^48^ Haem iron is highly bioavailable, while non-haem iron absorption is significantly influenced by enhancers (e.g., vitamin C, organic acids) and inhibitors (e.g., phytates in cereals, polyphenols in tea and coffee, and calcium in dairy).^49^ Dietary fibre and dairy products may also interfere with non-haem iron absorption.^50^ Micronutrients, such as calcium and zinc, can inhibit iron absorption when present in a high molar ratio to iron and particularly when supplemented (appendix p. 23).^51^ Unlike other inhibitors, calcium affects both non-haem and haem iron absorption.^52^ Despite advancements in understanding non-haem iron absorption, the mechanisms of haem iron absorption and regulation remain poorly understood.^53^ Moreover, current evidence, based on single-meal studies, does not adequately reflect impacts from whole diets.^54^

Several approaches have been developed to measure iron bioavailability. Early methods relied on single-meal iron isotope studies, while later approaches have used population data to assess the effects of iron absorption inhibitors and enhancers (appendix p. 23).^55^ However, there is still no consensus on which algorithm(s) generate definitive data for predicting the dietary bioavailability of iron. ^54^ Evaluating long-term iron intakes of a population benchmarked against physiologic needs would provide insights into the population epidemiology of iron-deficient diets, identifying subgroups at risk of inadequate or excessive intake, which could inform public health nutritional strategies.^54^

##### b) Dietary patterns and iron deficiency and anaemia

Current evidence on the relationship between dietary factors and anaemia mainly focuses on individual nutrients or foods. While logical, this approach does not capture the dietary patterns (types, quantities and proportions of foods in daily diets) in populations or the interactions among foods and nutrients.^56^ Several tools have been developed to assess dietary patterns. These include minimum dietary diversity scores, the Alternative Healthy Eating Index to estimate micronutrient adequacy and food security, the Global Diet Quality Score (GDQS) to compare diet quality across nations, and dietary modelling software like Optifood to identify nutrient gaps and optimise diets using local foods (appendix p. 24).^56^Analytical methods, such as factor analysis, principal component analysis, and Treelet approaches are often used to identify dietary patterns linked to diet-related chronic diseases, for example, the Mediterranean and Dietary Approaches to Stop Hypertension (DASH) diets (appendix p. 24).^56^ While dietary pattern analysis has been widely used to study the links between diet and chronic disease (appendix p. 24),^57^ the use of these techniques to assess dietary intake in the field remains complex and there is a need for further development and validation of these tools in diverse populations and contexts.

Studies have reported negative associations between certain dietary patterns and increased risk for ID and anaemia. Western diets, characterised by ultra-processed foods, red and processed meats, sugary drinks, and refined carbohydrates, are linked to poor nutrient intake, low-grade inflammation, and gut dysregulation, all of which could increase the risk of ID and anaemia.^58^ Plant-based diets, such as vegetarian and vegan diets, and traditional diets in LMICs that are based primarily on cereals, tubers, and legumes, with limited animal-source foods, may also contribute to ID and anaemia due to the lower bioavailability of non-haem iron from plant-based sources (appendix p. 24).^59^ On the other hand, the Mediterranean diet, rich in fruits, vegetables, legumes, whole grains, and olive oil, has shown several health benefits, including improved micronutrient status and potential benefits for iron levels.^60^ However, further research is needed to assess how overall dietary patterns, including traditional diets in LMICs, affect iron status and anaemia risk across diverse populations. Without understanding diets holistically, dietary interventions that solely focus on increasing iron-rich foods or bioavailable iron will only partly address ID and anaemia. While the role of individual nutrients in iron absorption is well-studied, there is limited research on how entire dietary patterns influence iron metabolism. Mechanistic studies are required to explore the synergistic effects of various foods and nutrients in different dietary contexts, in relation to iron absorption.

#### Co-occurrence of anaemia and malnutrition

The co-occurrence of undernutrition (wasting, stunting and underweight) and anaemia is common, especially in low-resource settings, and is driven by several interlinked mechanisms that reflect both nutritional deficiencies and broader physiological processes as well as overlapping risk factors for both anaemia and malnutrition. Studies in LMICs consistently show a high prevalence of anaemia among stunted or wasted children. For instance, data from 43 LMICs showed that 56·4% of preschool children were anaemic, with 21·5% experiencing both anaemia and stunting and in Bangladesh, stunted children and women with low body mass index (BMI) had a higher risk of anaemia than their non-malnourished counterparts (appendix p. 24).^61^ However, the mechanisms linking malnutrition to anaemia are not fully understood, but may include impaired micronutrient absorption and disrupted erythropoiesis, especially in cases of severe malnutrition. Severe acute malnutrition (SAM) contributes to anaemia through multiple pathways involving protein and micronutrient deficiencies, impaired erythropoiesis, and increased susceptibility to infections. For instance, oedematous SAM is associated with secondary immune deficiency, intestinal dysbiosis, epithelial barrier disruption, and growth faltering, all of which exacerbate inflammation and worsen immune dysfunction (appendix p. 24).^62^ The compromised epithelial barrier in children with oedematous SAM also increases the risk of micronutrient deficiencies, including iron, zinc, and vitamin A.^63^

The triple burden of malnutrition, comprising undernutrition, micronutrient deficiencies, and overweight, is now recognised in many LMICs, and is driven by multiple risk factors including inadequate diets, infections, inflammation, gastrointestinal dysfunction, and physiological demands. To address the triple burden of malnutrition, it is important to understand the underlying risk factors and biological interactions rather than examining them in isolation.^64^ Undernutrition, particularly stunting and wasting, is monitored well in most LMICs due to high prevalence and clear health consequences. However, anaemia and iron and other micronutrient deficiencies may not be as well characterised in the same populations due to challenges around blood testing, potentially leading to the co-occurrence of undernutrition with anaemia being overlooked. As discussed previously in Section 1, appropriate survey platforms would be useful in simultaneously capturing both anthropometry and anaemia data across populations.

#### Nutritional interventions addressing anaemia:

To boost the possibility of achieving impact at scale and thus achieving the set anaemia targets, we highlight the most impactful interventions for anaemia control based on available evidence.

##### Oral iron supplementation

Oral iron supplementation is a common nutritional intervention in the global health context for the prevention or treatment of ID and IDA. There is a wide range of oral iron supplements available on the market, which vary in composition, bioavailability, price, and other factors. Iron salts (e.g., ferrous sulphate or ferrous fumarate) are the most common iron supplements used in the global health context.

The dosage of oral iron supplements depends on the indication for use (prevention vs. treatment of ID), their form, and the duration and course of the proposed intervention. In settings where anaemia prevalence exceeds 40%, WHO recommends universal iron supplementation for women and children through several established global guidelines (appendix p. 24).^65^ These guidelines are based on the assumption that ID is the most common cause of anaemia in these settings. However, multiple causes of anaemia commonly interact (e.g., nutritional deficiencies other than iron, haemoglobinopathies, inflammation, infections) and ultimately, iron interventions such as iron supplementation will only be efficacious at reducing the anaemia burden attributed to ID.^5^ Preventive doses of elemental iron can range from 30–60 mg daily for non-pregnant WRA, 10–12·5 mg for children aged six to 23 months, 30 mg for children aged 24 to 59 months, and 30–60 mg daily for children aged 5–12 years (appendix p. 24).^65^ During pregnancy, a preventive daily dose of 30 to 60 mg elemental iron (along with 0·4 mg folic acid) is recommended to prevent maternal anaemia, puerperal sepsis, low birth weight and preterm birth, which can be taken alone or within a multiple micronutrient supplement. ^66^ In malaria-endemic settings, WHO recommends that universal iron supplementation be provided alongside effective malaria prevention and treatment measures (appendix p. 24).^66^

Oral iron can be an efficacious intervention for treating ID, but oral iron dosing varies widely across guidelines and settings. Previous guidelines recommended high dose oral iron provided in split dosing, but this is no longer advisable. After the ingestion of oral iron, hepcidin is upregulated and consequently results in limited absorption.^67^ Therefore, bioavailability of oral iron supplements may be limited with daily ingestion. More recent research with stable isotopes indicates that dosing on alternate days may be a more efficient strategy for improving iron stores in non-pregnant women,^68^ although a more rapid increase in iron stores is achieved through daily dosing of iron. Thus, dosing daily or every other day is generally used depending on patient adherence and tolerability, and the necessity for rapid increase in iron stores.^69^

During pregnancy, countries may choose to use prenatal multiple micronutrient supplements (MMS) (e.g. containing 30–60 mg of iron) as a preventive strategy, combined with other WHO-recommended context-specific preventative measures for anaemia (e.g. antenatal deworming and intermittent preventive treatment of malaria). If women develop anaemia, additional iron should be provided while daily prenatal MMS are continued as a preventive measure throughout pregnancy, as would be done with iron and folic acid supplementation. Ideally, women presenting with anaemia should undergo further diagnostic testing, such as measurement of iron status, to identify the underlying cause of the anaemia. Recent guidance shows possible combinations of both MMS and iron supplements to treat mild to moderate anaemia, to reach an intake of 90 to 120mg of daily elemental iron.^70^ However, the optimal dose of oral iron for treating anaemia during pregnancy remains uncertain. Severe anaemia should follow the local standard of care, which may include intravenous iron or packed red blood cell transfusion.^70^

##### Intravenous iron

An alternative to oral iron is intravenous (IV) iron. Unlike previous IV iron drugs, third-generation intravenous iron formulations do not appear to cause anaphylaxis and enable a total dose iron replacement to be delivered over a rapid infusion (e.g. 1000–1500mg over 15–30 minutes, depending on the formulation). The main advantage of IV iron over oral iron treatment is its bioavailability: it bypasses gastrointestinal tract absorption, thereby minimising associated side effects and concerns about adherence to oral iron.^71^ A meta-analysis has shown that IV iron interventions, compared with oral iron, are more effective in improving Hb levels at delivery.^72^ However, few trials included in this meta-analysis have examined the effects of third generation IV iron formulations, or were conducted in low-income countries, particularly in the African region.

Recent studies have made a start in addressing these issues. For example, two large randomised controlled trials in a resource-limited, malaria-endemic setting showed that IV ferric carboxymaltose (FCM, a third generation IV iron formulation) is safe in pregnant women in their 2nd trimester,^73^ or 3rd trimester of pregnancy.^74^ FCM was more effective than oral iron in reducing anaemia, ID, and IDA when administered during the 3^rd^ trimester^74^, as well as in reducing ID and IDA when administered in 2^nd^ trimester.^73^ Long term follow-up from the second trimester trial showed that IV iron reduced anaemia prevalence in mothers for six months post-partum, and ID and IDA prevalence for at least 12 months post-partum.^75^ However, there was no impact of IV iron on neonatal or infant iron or haemoglobin status.^75^A larger trial comparing oral iron to FCM and iron derisomaltose (another third generation formulation) to treat iron deficiency anaemia in rural India found evidence that FCM may reduce the risk of low birth weight, and that neither IV formulations clearly enhanced the probability of achieving a non-anaemic state.^76^

A key challenge remains the high cost of third generation IV iron formulations, although there are potential savings in clinic personnel time and bed space. IV iron sucrose (an older formulation) may be cheaper but requires recurrent clinic visits to deliver an equivalent iron dose. ^77 73,74^Health economic analyses to address the cost-effectiveness of third-generation IV iron formulations in managing anaemia in pregnant women in resource-limited settings are needed to evaluate this intervention for translation to policy.

A risk of IV iron formulations (particularly FCM, less commonly with derisomaltose) is transient hypophosphataemia. Recurrent doses of FCM have been associated with osteomalacia.

##### Multinutrient supplementation to tackle micronutrient deficiencies known to cause anaemia

The first key outcome identified in the WHO framework for accelerating anaemia reduction is improved micronutrient status, which can be achieved through dietary diversification, food fortification, and supplementation.^5^ The interventions recommended to improve micronutrient status vary by target population, context and setting. For example, iron supplements alone or with other micronutrients, or point-of-use fortification with multiple micronutrient powders (or lipid-based nutrient supplements) is recommended for infants and young children (six to 23 months of age) in populations where anaemia prevalence exceeds 40%.^46,78,79^ In a double-blinded trial, iron supplements and multiple micronutrient powders produced similar effects on iron status and haemoglobin levels in infants.^5,80^ In WRA, WHO has specific recommendations of iron and/or folic acid supplementation based on pregnancy status and anaemia prevalence.^5^ In all other populations, screening to detect nutrient deficiencies with provision of appropriate follow-up (therapeutic micronutrient supplementation) is recommended.^5^

Folic acid supplementation is recommended for pregnant women from three months before conception to 12 weeks’ gestation to reduce the risk of neural tube defects and maternal anaemia. Since many pregnancies are unplanned and often discovered close to 12 weeks, improving population-level folate status through strategies like staple food fortification is crucial. Additionally, in malaria-endemic regions, increased folate demand due to haemolysis and fever further raises the risk of folate deficiency. More research is needed to evaluate a concern that folic acid supplements may contribute to treatment failure of antifolate antimalarial drugs.^81^

The positive effect of supplementation with micronutrients other than iron on anaemia has been demonstrated. Vitamin C supplementation not only enhances the absorption of non-haem iron but also increases haemoglobin concentration and serum ferritin in children and non-pregnant women, even without iron (appendix p. 25).^82^ Vitamin A supplementation may reduce the risk of anaemia and increase haemoglobin concentrations across various populations, including children, adolescents, and pregnant and lactating women, even without iron supplementation.^83^ These results support the need to assess vitamin A status in anaemia reduction programmes, especially in regions where vitamin A deficiency and anaemia coexist. Some studies show that vitamin B2 supplements co-administered with iron have a greater effect on increasing haemoglobin concentrations than iron supplements alone, particularly in anaemic school children.^46,82^ A similar effect was reported for vitamin B6 when used concomitantly with iron in anaemic pregnant women.^84^

There is potential for a synergistic or antagonistic effect of multiple micronutrient supplementation on anaemia, although this remains understudied. A meta-analysis of studies comparing multiple micronutrient supplementation (providing 30 mg iron/day) to iron and folic acid supplementation (60 mg iron/day) during pregnancy found no difference in the risk of maternal anaemia in the third trimester. In these study populations with a high prevalence of anaemia (29%–47%), there was a similar effect on maternal anaemia (RR 0·99, 95% CI: 0·92–1·07) despite the multiple micronutrient supplements providing only half of the iron dose provided by the iron and folic acid supplements. This suggests that the presence of other nutrients in the multiple micronutrient supplements (zinc, chromium, vitamins A, B2, B6, B9, B12, C, D and E) may have an important role in anaemia prevention.^85^ An alternative explanation is that there is no further haematological benefit for doses of iron above 30mg/day. A meta-analysis of systematic reviews on various interventions targeting anaemia endorsed the strategy of addressing nutrient-related anaemias beyond solely IDA.^86^ Further research is needed to determine the most effective combination of micronutrients to address anaemia in different populations.

##### Industrial food fortification

Industrial food fortification, the process of adding micronutrients to commonly consumed foods, is a widely endorsed strategy for combating micronutrient deficiencies that lead to anaemia, particularly ID. Fortifying staple foods like wheat flour, rice, and condiments like salt, with iron, folic acid, and other essential nutrients can significantly reduce the prevalence of anaemia in LMICs.^87^ This approach is advantageous due to its broad reach, targeting large populations with minimal behaviour change. However, challenges include variability in fortification compliance, bioavailability of added nutrients, and the need for ongoing monitoring to ensure effectiveness.^88^ Inaccurate selection of fortification vehicles, for example, staple foods not widely consumed by target groups, can also limit the impact of fortification on vulnerable subpopulations including women and children.^89^

There is a need for the development of advanced fortification techniques to enhance the nutrient content of staple foods, providing multiple micronutrients simultaneously. Additionally, more research on innovative delivery mechanisms and formulation strategies is needed to ensure the stability and bioavailability of fortified nutrients. Case studies such as the successful fortification of staple foods with iron and other micronutrients in Costa Rica and Indonesia and rice fortification in India and Bangladesh serve as models for effective implementation (appendix p. 25).^90^

##### Point-of-use fortification

Point-of-use fortification, where micronutrient powders are added directly to foods during preparation, offers greater control over individual nutrient intake and can be particularly effective in settings with limited access to fortified foods. However, this approach requires more active participation from the community and consistent use, which may limit its impact.^91^ A balanced strategy that includes industrial and point-of-use fortification alongside interventions to improve diet quality and healthcare access offers the most promising route to reducing anaemia at population level.

##### Food preparation techniques to improve nutrient intake

Food preparation techniques could play an important role in improving nutrient intake by enhancing bioavailability and preserving essential nutrients. Methods like steaming, blanching, and fermentation can boost nutrient retention while reducing anti-nutrients like phytates and tannins that can inhibit iron absorption (appendix p. 25).^92^ Food pairing strategies such as combining iron-rich plant-based foods with vitamin C sources can also boost non-haem iron absorption.^93^

Cooking with iron-containing pots and ingots like the Lucky Iron Fish (LIF) has been explored as a household strategy to address ID (appendix p. 25).^94^ These cookware release iron when cooking acidic foods, enhancing the solubility and availability of iron in the diet. However, their effectiveness with non-acidic foods is limited. With reasonable compliance (~26–71%), there is potential for these cookware to reduce ID in LMICs.^95^ However, concerns about leaching of harmful metals, including lead and cadmium, highlight the need for further research to assess their safety and efficacy before widespread adoption.^96^

##### Breastfeeding to control maternal and infant anaemia

Exclusive breastfeeding during the first six months is vital in preventing anaemia in both infants and mothers. For mothers, breastfeeding stimulates uterine contractions, reducing postpartum bleeding and thereby helping to address early postpartum anaemia.^97^ Exclusive breastfeeding also promotes lactational amenorrhea, delaying the return of menstruation and thereby reducing iron losses associated with menstrual blood loss. It can also act as a natural contraceptive, facilitating birth spacing and reducing the likelihood of closely spaced pregnancies, which could otherwise deplete maternal iron stores.^98^ Prolonged exclusive breastfeeding beyond six months increases the risk of anaemia at age 12 months.^99^ As infants’ iron stores decline around six months, introducing iron-rich complementary foods is essential to prevent ID and anaemia during critical developmental stages. Children aged four to six years who were introduced to complementary foods, particularly iron-unfortified plant-based foods at three to six months of age have a higher risk of anaemia compared with those who began complementary feeding at six months.^100^

There remains ongoing uncertainty regarding several aspects: 1) the impact on infant iron stores of the timing of complementary food introduction; 2) if and whether iron supplementation (e.g. in lactating women or in infants before six months of age) is merited and in which cases, and 3) approaches to optimise the nutritive quality of complementary foods, especially in LMICs.

##### Agriculture-nutrition programmes

Agriculture-nutrition programmes are initiatives designed to integrate agricultural practices with nutrition objectives to improve food security and nutritional outcomes. These programmes are a crucial channel for providing opportunities for reaching large segments of the population. This could be done through promoting various production approaches such as biofortification of staple crops and as a delivery platform for additional strategies to improve nutrition.^101,102^ Popular approaches such as integrated school and home gardens have shown mixed results on anaemia reduction, whilst interventions that integrated other strategies, such as nutrition education, hygiene improvement, and women’s empowerment, and training on marketing skills, appear to achieve anaemia reduction in children.^103^ Agriculture is especially important as it serves as an avenue to reach poor and marginalised populations, thus enabling equitable and sustainable access even beyond programme implementation.^102^ These programmes can contribute to more diverse diets and potentially generate income, leading to better preventive healthcare seeking and potentially preventing anaemia. While agriculture-nutrition programmes hold promise in addressing anaemia, more robust evidence is needed to identify effective programme models in different settings and assess their real-world effectiveness beyond efficacy trials.^101^

In addition, there is a need for research investment in biofortification research to breed crops with enhanced nutrient profiles, focusing on staple crops consumed by vulnerable populations. Genetic modification and traditional breeding techniques can be used to increase the content of essential micronutrients such as iron, zinc, and vitamin A in crops.^104^ Success stories like the introduction of vitamin A-biofortified orange-fleshed sweet potatoes in sub-Saharan Africa underscore the potential of this approach in addressing nutrient deficiencies.^105^ Further, there is a need to assess the iron content, bioavailability, feasibility, cost-effectiveness and acceptability of incorporating insects and artificial meat into diets to combat anaemia (appendix p. 25).^106^ Although these techniques are nascent, in the longer term they could offer cost-effective and environmentally sustainable sources of nutritional iron. Moreover, it is essential to explore how these agricultural interventions could complement other food systems approaches, such as food fortification, and investments in the health sector, such as supplementation, to optimise impact.

##### Nutritional education and awareness

Nutrition education and counselling has long been used to address anaemia and has broad global coverage (appendix p. 26).^107^ One key benefit is that improving diet quality could have positive health impacts beyond anaemia. However, nutritional education focusing solely on imparting knowledge may be limited in changing dietary practices. Incorporating Social and Behavioural Change (SBC) approaches can strengthen these efforts by addressing not only awareness but also the barriers and facilitators that influence attitudes and behaviours.^108^ SBC strategies consider social, cultural, and environmental factors to promote sustainable behaviour change and improve dietary practices. Future strategies could integrate modern communication techniques and leverage innovative tools like mobile applications and wearable devices for personalised education on nutrient-rich diets for anaemia prevention.^109^ Healthcare providers could utilise social media and digital platforms to disseminate evidence-based nutrition information while personalised dietary recommendations could be tailored to genetic profiles, lifestyle, and nutrient requirements (appendix p. 26).^110^ Community-and-school-based nutrition education and school meal programmes should target vulnerable populations, incorporating culturally appropriate messaging and integrate comprehensive nutrition education curricula into school systems.^111^ Governments should ensure supportive policies are in place, prioritising nutrition education in healthcare settings, schools, workplaces, and communities.^111^ Additionally, integrating nutrition counselling into routine clinical practice will ensure that all anaemia patients receive proper guidance. Finally, robust evaluation and monitoring mechanisms are essential to assess the effectiveness and impact of these programmes and guide ongoing improvements.^111^

#### Other interventions addressing anaemia:

##### Optimising iron stores through delayed cord clamping

An infant’s iron stores at birth are largely determined by the timing of umbilical cord clamping.

Delayed Cord Clamping (DCC) is the practice of postponing the clamping of the umbilical cord for one to three minutes after birth or until one minute after the cord pulsations have ceased.^112^ This procedure ensures the additional placental transfusion of approximately 80–100ml of blood, increasing the neonates’ total blood volume by 20–30% and elevating the red cell volume by 60% (appendix p. 26).^113^ A 2-minute DCC can increase iron stores by 27–47 mg in full-term infants at six months.^114^ The persistence of increased neonatal haematocrit and haemoglobin levels, potentially up to twelve months post-birth (appendix p. 26),^115^ has further underscored DCC’s positive effect on preventing IDA. This was evident in a randomised controlled trial of newborns born to anaemic mothers in India, which showed that by the age of three months, newborns who received DCC were less likely to have haemoglobin levels <10g/dL (44% vs 86%, OR: 7·7 CI, 1·84–34·9).^116^

Despite the evident benefits for nearly all newborns, information relating to the coverage and quality of DCC remains scarce and varied. High-income countries exhibit relatively high DCC adoption (appendix p. 26).^117^ However, there are few studies in high anaemia burden settings, where neonatal mortality and prematurity rates are the highest, ^118^ and thus where DCC would likely have the most significant impact.^119^ Moreover, despite having guidelines in place there is considerable variability in reported coverage rates of DCC in nations with the highest burden of anaemia, ranging from 4% to 98% (appendix p. 26).^120^ Current research also highlights several challenges associated with DCC, including insufficient knowledge and training, poor staff-to-newborn ratios, overwhelming workloads, infrastructure and resource inadequacies, and guidelines awareness and access (appendix p. 26–27). ^121^

We propose a need for a comprehensive multimodal toolkit for DCC implementation to address these gaps, similar to healthcare worker hand hygiene.^122^ This toolkit could be equipped with accessible monitoring and evaluation mechanisms and paired with strategic methodologies to elevate practice quality and extend its coverage to other settings. Additionally, there is a demand for robust quantitative evidence focusing on adherence to and quality of DCC across high-burden settings.^123^ Complementing this with qualitative insights from clinical professionals and mothers could enrich the current understanding of enhancing DCC adoption across varied settings.

##### Utilising cash transfer programmes to improve nutrition and address anaemia

Economic constraints are a main barrier to accessing diverse, nutrient-rich diets, especially for vulnerable populations. In low-resource settings, food insecurity and limited purchasing power contribute to inadequate intake of iron and other essential nutrients, increasing the risk of anaemia.^124^ Traditional social protection approaches to anaemia largely rely on in-kind transfers, which provide goods or services instead of cash. These include nutritional interventions such as food in-kind transfers to improve the diets of children or pregnant women (appendix p. 27).^125^ Cash transfer (CT) programmes play a key role, including alleviating families’ budget constraints and helping to smooth consumption.^126^ Conditional cash transfers (CCT) provide cash payments to individuals or households in exchange for performing specific activities such as attending antenatal visits and other human capital services.^127^

CT programmes have led to reduced likelihood and prevalence of anaemia and improved haemoglobin levels among children and WRA within the first two years of exposure, and even ten years after exposure to the CCT (appendix p. 27).^126^ CT programmes have also been used as an incentive to increase iron supplementation intake during pregnancy.^128^ These programmes are effective when complementing nutrition-specific interventions, and they can serve as a base for large-scale delivery platforms for nutritional programmes.^129^ While some CT programmes have improved diet quality and empowered women,^130^ receiving cash does not automatically translate into healthier diets. Households may prioritise other expenses or opt for cheaper, calorie-dense foods over iron-rich options. Cultural norms can also influence food distribution, for instance, in a study conducted in a rural district of Pakistan, meals were served first to men in nearly half of households, and only first given to children in about one-fifth of households.^131^

A range of research priorities emerge. The effectiveness of CT programmes on anaemia could be better benchmarked against other demand and supply-side barriers, e.g. access and availability of nutritious foods.^132^ This widening of the evaluative horizon echoes considerations made in CT approaches for other human capital formation e.g. child learning, and reproductive health (appendix p. 27).^133^ Similarly, more work on cost-effectiveness across different contexts is needed. This would call for the development of robust, consistent protocols for the identification and attribution of costs to particular programmes.

### Inflammation, infections and anaemia

#### Anaemia of inflammation: the role of hepcidin

Hepcidin, a hormone regulating iron levels, is a key player in inflammation-related anaemia by limiting iron availability for red blood cell production.^36^ During a steady state, little iron is absorbed from the diet or lost, with most of the iron requirement being met by recycling iron from macrophages during red blood cell turnover. During infection, the immune system releases pro-inflammatory cytokines such as interleukin-6 that up-regulate the production of hepcidin (appendix p. 27).^134^ Hepcidin, in turn, regulates the levels of circulating iron by degradation and occlusion of ferroportin on enterocytes and macrophages. Sustained expression of hepcidin during infection or inflammatory conditions including autoimmune diseases such as rheumatoid arthritis and inflammatory bowel disease, cancer, and chronic disorders such as kidney disease and heart failure results in anaemia due to reduced availability of iron for erythropoiesis (functional ID).^135^ In the elderly, low-grade chronic inflammation (‘inflammaging’) may also contribute to anaemia. Obesity has also been linked to inflammation and hepcidin upregulation which may also drive functional ID. The prevalence of overweight and obesity is rising in LMICs and globally. Children who are overweight and obese have poor iron status and a reduced response to iron interventions.^136^ Despite similar iron intake as their normal-weight peers, overweight school-aged children’s higher BMI was linked to elevated levels of soluble transferrin receptor (sTfR), hepcidin, and inflammatory markers, suggesting that iron availability for erythropoiesis was reduced due to impaired iron absorption and increased iron sequestration.^137^ These findings highlight the impact of obesity-associated inflammation and altered iron metabolism on anaemia risk, highlighting the importance of addressing obesity as part of anaemia prevention strategies across all age groups. Hepcidin also regulates placental iron transport to the foetus during pregnancy (appendix p. 28).^138^

Anaemia control is a major challenge in settings where chronic or asymptomatic infections are associated with chronically raised hepcidin. It is crucial to understand how hepcidin levels change in different population groups during infections because these can inform strategies for controlling anaemia caused by inflammation. For instance, in pregnant women, the mechanism underlying maternal hepcidin suppression as pregnancy progresses is still unknown,^139^ and the impact of maternal ID on infections has not been extensively researched.^140^ Recently developed treatment strategies targeting hepcidin to release sequestered iron from the reticuloendothelial system appear to be a promising alternative to conventional iron supplementation, particularly for managing anaemia of inflammation. However, the effectiveness of these therapies relies on accurately diagnosing and distinguishing inflammation-related anaemia from other anaemia causes such as IDA or mixed aetiologies.^141^ Thus, it is crucial to gain a more thorough understanding of the molecular mechanisms that drive anaemia of inflammation.^142^

#### Anaemia and Malaria

Malaria is a vector-borne parasitic infectious disease affecting humans worldwide in tropical and subtropical regions. Among the five protozoan parasites within the genus *Plasmodium*, *P. falciparum* is the deadliest, accounting for 99·7% of severe cases and mortality, primarily in WHO African region. WHO’s 2023 malaria report estimated 249 million cases and 608,000 deaths from malaria in 85 malaria-endemic countries during 2022, with WHO African region accounting for 94% of these cases.^143^

Malaria is a major cause of anaemia in endemic areas and is one of the most common reasons for blood transfusion in areas of high transmission.^144^ Severe malarial anaemia caused by *P. falciparum* is responsible for approximately one-third of malaria-related deaths, mostly in children under five.^144^ Children admitted to hospital with severe malaria anaemia have a high post-discharge mortality rate, up to a year after discharge.^145^

The red blood cells (RBCs) are the primary target of infection in humans by *Plasmodium* species. The pathophysiology of anaemia in malaria is multifactorial, involving both haemolysis and decreased erythropoiesis (appendix p. 28).^146^ *Plasmodium*, being an intraerythrocytic parasite, leads to the obligatory destruction of RBCs containing parasites upon schizont rupture. However, a more significant factor is the accelerated destruction of non-parasitised RBCs, which correlates with malaria severity.^146^ It is estimated that the loss of unparasitised erythrocytes accounts for approximately 90% of the acute anaemia resulting from a single infection. Other factors influence how malaria causes anaemia, including age, pregnancy, antimalarial immune status, the genetic constitution of infected individuals, and the intensity of malaria transmission.^147^

The WHO and the Roll Back Malaria Partnership have suggested using anaemia as a community-level malaria-burden indicator, especially as malaria control interventions are expanded.^148^ It suggests that anaemia prevalence may respond more rapidly than mortality to the scaling up of malaria interventions, such as insecticide-treated nets, malaria chemoprevention, and indoor residual spraying (appendix p. 28).^149^

Sub-patent malaria where *Plasmodium* parasites are undetectable in blood by microscopy or rapid diagnostic testing, but detectable by PCR, is associated with an increased risk of anaemia (appendix p. 29).^150^ The burden of sub-patent malaria is difficult to evaluate in endemic areas due to the limitation of diagnostic tools and techniques. Hence, novel public health strategies for identifying and reducing the hidden burden of asymptomatic and sub-patent infections should focus on using more sensitive molecular diagnostic methods to limit malaria transmission. Asymptomatic *P. falciparum* doubles hepcidin concentrations, impairing iron absorption and utilisation (appendix p. 29). ^151^ This suggests that providing iron interventions without first treating the infection will not address anaemia and routine iron supplementation may be potentially harmful in malaria-endemic settings.^152,153^ Treatment of malaria including asymptomatic cases can substantially reduce inflammation and hepcidin concentrations, increasing iron absorption, and reducing iron deficiency and anaemia (appendix p. 29).^153^ Therefore, integrated control strategies targeting both malaria and anaemia are essential in endemic settings.

#### Tuberculosis

Tuberculosis, caused by *Mycobacterium tuberculosis*, is the primary cause of death globally from a single infectious disease, resulting in 10 million new cases and 1·5 million deaths annually.^154^ Anaemia is a frequent occurrence in tuberculosis, affecting up to 88% of patients (appendix p. 29).^155^ The development of anaemia in tuberculosis is influenced by multiple factors, but hepcidin-driven functional ID is typically predominant.^156^

Although the role of hepcidin in anaemia caused by inflammation has been previously addressed, there are several specific interactions between tuberculosis, iron levels and anaemia (appendix p. 30).^36,156^ Excess iron significantly boosts the growth of *M. tuberculosis* in laboratory settings and in mouse models (appendix p. 30)^157^, and in humans, a diet rich in iron is linked to a greater risk of developing tuberculosis.^158^ However, both ID and anaemia are associated with higher mortality rates in tuberculosis patients.^159^

Similar to other chronic infections, distinguishing between anaemia due to inflammation and IDA in patients with tuberculosis is challenging since commonly used markers of iron status, such as ferritin and, to a lesser extent, soluble transferrin receptor, are influenced by inflammation. A study measuring iron absorption among adults diagnosed with tuberculosis reported elevated levels of erythroferrone, inadequate hepcidin suppression, and persistently low iron absorption during active tuberculosis even with intensive treatment.^160^ However, despite these impairments, mobilisation of sequestered iron supported a rapid increase in red blood cell production during early treatment, leading to haemoglobin recover.^160^ These findings suggest that iron supplementation before and during tuberculosis treatment may be unnecessary, aligning with previous studies (appendix p. 30).^156^ These data also argue that iron supplementation should be reserved for tuberculosis patients who remain anaemic after completing treatment.^160^ There is need for further evidence on whether and when to administer iron to anaemic tuberculosis patients.^160^ Providing iron-rich foods, especially those containing haem iron, may be crucial for haemoglobin recovery during tuberculosis treatment, particularly during the later stages of treatment when acute inflammation has been alleviated.

#### HIV disease

In 2019, 1·8 million children aged 0–14 years were living with HIV (HIV+) worldwide.^161^ Anaemia frequently complicates paediatric HIV infection, and is an independent marker for disease progression and mortality (appendix p. 30).^162^ In HIV+ adults, it is estimated that iron deficiency (ID) accounts for 20–44% of anaemia and functional ID accounts for 41–47%, but these often overlap.^162^

The complex aetiology of anaemia in HIV includes nutritional, inflammatory and HIV-specific determinants, for example, side effects from antiretroviral drugs such as zidovudine (ZDV), and impaired erythropoiesis due to disrupted bone marrow function (appendix p. 30).^163^ ZDV-based treatment has been shown to increase anaemia prevalence and has been linked to an increased likelihood of developing severe anaemia, as ZDV is a possible inhibitor of erythroid colony-forming units, which could reduce red blood cell production (appendix p. 30).^164^ HIV infection can alter iron distribution differently in various tissues, with increased iron levels in the liver and macrophages and ID in others.^165^

This complexity, together with the small sample sizes of randomised controlled trials, might have contributed to the inconclusive findings of the effectiveness and safety of iron supplementation among HIV-infected people.^162^ Similar to other infections, another knowledge gap in the underlying mechanism in anaemic HIV+ patients is how the opposing effects of ID and inflammation influence hepcidin concentrations, iron homeostasis, and dietary iron absorption.

#### Anaemia due to soil-transmitted helminths and schistosomiasis

Soil-transmitted helminths (STH) and schistosomiasis are common human parasitic infections that cause anaemia. They affect populations with limited access to clean water, sanitation, and hygiene. Approximately 1·5 billion people worldwide are affected by STH, which are transmitted when walking on soil contaminated with infected human faeces.^166^ School-age children bear the highest burden of STH. Hookworms (*Necator americanus and Ancylostoma duodenale*) inhabit and attach to the small intestine, where they feed on blood, leading to chronic blood loss.^167^ The extent of this blood loss depends on the severity of the infection, and in individuals with a moderate to heavy hookworm burden, it can result in IDA.^168^ Globally, hookworms infect up to 500 million people, particularly in tropical regions.^169^ Beyond hookworm, other STH species (e.g. whipworm, roundworm) may also impair intestinal health and may contribute to poor nutrition, including anaemia. Individuals may be infected with more than one species depending on exposure and endemicity (appendix p.30).^170^

Schistosomiasis infection, or ‘Bilharzia’, is caused by three main species infecting humans: *Schistosoma haematobium* (endemic in Africa and the Middle East), *S. mansoni* (Africa, the Middle East, the Caribbean and South America) and *S. japonicum* (mainly in China, Indonesia, and the Philippines). According to WHO, at least 251·4 million people required preventive treatment in 2021, with 90% of these cases in sub-Saharan Africa.^171^ The species *S. mansoni, and S. japonicum* are associated with intestinal schistosomiasis, whereas *S. haematobium* causes urogenital infection and chronic urinary blood loss, resulting in IDA.^172^ Schistosomes cause intestinal and urogenital blood loss as eggs translocate across the intestinal or bladder wall. Importantly, inflammation from schistosomiasis may cause anaemia by upregulating hepcidin, which blocks iron absorption and recycling, leading to IDA.^173^ Schistosomiasis primarily affects individuals in contact with infested water, with parasite burden increasing with age and peaking between ages 10 and 20 years, then declining as partial immunity develops.^174^ Both STH and schistosomiasis are a major cause of anaemia in at-risk groups living in endemic areas. In populations with heavy infection up to 25% and 32% of anaemia is attributable to STH and schistosomiasis, respectively.^175^

Notably, STH and schistosomiasis co-exist in the same regions as malaria, and the combination of malaria-induced haemolysis with chronic blood loss from STH and schistosome infections can significantly exacerbate the risk of anaemia. Studies show that coinfection with malaria and STH or schistosomiasis is associated with a threefold increase in anaemia risk, much higher than the risk from either infection alone (appendix p. 30).^176^ This highlights the need for an integrated approach to controlling malaria, STH, and schistosomiasis where these infections co-exist, especially among at-risk groups such as school-age children and pregnant women.

Anaemia associated with STH and schistosomiasis can be managed with anthelmintic treatment and iron supplementation. Currently, the benzimidazole anthelmintics, mebendazole and albendazole, are the primary treatments for STH.^170^ Schistosomiasis is mainly treated and controlled using praziquantel. The impact of anthelmintic treatment on anaemia is greatest when albendazole is co-administered with praziquantel.^168^ Despite the availability of anthelmintic drugs in endemic countries, only about half of at-risk children receive treatment, falling short of the global target of 75%. ^170,177^ This highlights the need to scale up coverage of anthelmintic treatment to effectively reduce anaemia on a global scale.

The control of STH and schistosomiasis relies on a comprehensive approach that includes large-scale treatment of at-risk population groups, access to safe water, improved sanitation, hygiene education and behaviour change, and snail control and environmental management.^177^ Although school-based deworming programmes have reduced the burden of STH (appendix p.30), ^177^ treatment of adults might also be required to control these parasites.^178^ However, reviews of randomised clinical trials found no overall population-level effect of deworming on child haemoglobin levels, indicating that additional interventions are needed together with STH control (appendix p. 31).^179^

There is a clear need for evidence to support a multifaceted approach that integrates treatment, WASH programmes and coadministration of micronutrients to address anaemia associated with STH and schistosomiasis. Additionally, the precise mechanisms through which STH and schistosomes cause anaemia are not fully understood and require further study. Evidence shows that combining multiple micronutrient fortification (iron, zinc, iodine and vitamin A) with deworming treatments is more effective at reducing helminth infections in school children than deworming alone.^180^ However, the interaction between micronutrient deficiencies, and parasitic infections in causing anaemia is poorly understood. Moreover, not all infected individuals develop anaemia, highlighting a need to understand the factors influencing variation in susceptibility.

#### The role of gut health in anaemia

Along with Environmental Enteropathy (EE), many other factors can influence gut health and, in turn, anaemia. EE, also known as environmental enteric dysfunction, is a chronic condition of the small intestine that is caused by multiple entero-pathogens including *Escherichia coli* and *Campylobacter* infections.^181^ EE is commonly found in individuals living in poor WASH conditions in LMICs and may contribute to anaemia, particularly in vulnerable populations like young children and pregnant women. Characterised by inflammation and altered gut mucosal structure, surface area and function, EE results in increased intestinal permeability and impaired absorption of essential nutrients. ^182^ EE is also associated with enteric microbiome dysbiosis. EE may contribute to anaemia by limiting the absorption of crucial micronutrients. Additionally, chronic gut inflammation may drive production of hepcidin, inhibiting iron absorption and further exacerbating ID.^181^ Anaemia itself may further contribute to increased intestinal permeability causing intestinal inflammation and barrier disruption (appendix p. 31).^183^

Some studies have indicated that oral iron administration may profile the intestine in favour of pathogenic bacteria and increase the risk of diarrhoea in children (appendix p. 31).^184^ Iron treatment for anaemia in individuals with diarrheal infections may be ineffective or deleterious.^185^ *Helicobacter pylori*, can impair absorption of vitamin B12 and iron, increasing the risk of anaemia.^186^

Several evidence gaps exist in understanding the role of gut health in anaemia. There is limited comprehensive data on the true prevalence and severity of EE in different populations, particularly in relation to anaemia. This is mainly due to a lack of standardised methods and biomarkers to diagnose EE accurately, and because histological alterations are common in most individuals exposed to poor WASH conditions in LMICs.^187^ Current methods of diagnosing EE are typically expensive and invasive (endoscopy) and unsuitable for use in the clinic or community in LMICs where EE is common. Additionally, while EE and gut dysbiosis are known to impair nutrient absorption, the exact biological mechanisms through which they contribute to anaemia, particularly the role of inflammation, intestinal permeability, and immune responses, are not fully understood. Further, there is a need to elucidate the dynamic interactions between different iron sources, gut microbiota composition, and host physiological responses. Moreover, exploring innovative strategies to manipulate gut microbiota to optimise iron absorption and mitigate anaemia risk represents a promising avenue for future interventions.

Treatment and control strategies for gut disorders, including EE, primarily focus on improving gut health, reducing inflammation, and addressing the underlying causes, such as poor WASH conditions and repeated enteric infections. Nutritional interventions on their own may not be sufficient to correct nutrient deficiencies due to EE-related nutrient leakage or malabsorption. Vaccination against key gut pathogens like rotavirus and norovirus, combined with early diagnosis of EE and diarrhoeal aetiologies, as well as timely treatment with antibiotics and rehydration, can help reduce bacterial overgrowth and alleviate the infection burden that contributes to EE and associated anaemia (appendix p. 31).^188^ However, long-term use of antibiotics can disrupt the gut microbiome or result in antimicrobial resistance complicating clinical treatment.^189^ Prebiotics, postbiotics, and probiotics may have the potential to restore healthy gut microbiota and prevent anaemia, although little is known about their therapeutic or preventive effects on anaemia.^190^ Since chronic gut inflammation is a key feature of EE, anti-inflammatory drugs developed for irritable bowel syndrome (IBS) may be considered as both conditions involve enteric inflammation and intestinal alterations. However, the safety of these therapeutics in at-risk groups, such as children in infection-endemic settings remains insufficiently studied.^191^

Given the apparent link between environmental exposures, EE and helminthic infections, several studies have examined the effectiveness of reducing environmental contamination through WASH programmes. While randomised controlled trials in LMICs have shown some evidence of modest beneficial effects of WASH interventions on undernutrition and infections,^192^ there is inconclusive evidence on the direct effectiveness of WASH programmes in anaemia reduction (appendix p. 31).^193^

Recently, there have been calls for “transformative WASH”.^194^ Even though there are ongoing debates as to what transformative WASH entails, some improvements have been suggested, including expanding the scope of WASH indicators beyond diarrhoeal infections and linear growth, to include other biosocial indicators such as nutrition, water and sanitation insecurity experiences, gender roles and mental health among others (appendix p. 31).^195^ In addition, there is growing recognition of the need for a context-specific, risk-based comprehensive package of WASH interventions that prioritise community needs and feasibility.^196^ It will be crucial to understand the effectiveness and role of transformative WASH programmes in anaemia reduction.

#### Anaemia in the elderly

The aetiology of anaemia in the elderly is complex and diverse and can be classified into three overlapping categories: 1) nutritional deficiencies including iron (due to poor nutrition, absorption or chronic blood losses), vitamin B12, and folate; 2) chronic inflammation from chronic disease including chronic kidney disease (CKD), infections, auto-immune diseases and age; 3) clonal haematopoiesis.^197^ In the remaining cases, the cause of anaemia remains unidentified, and is categorised as ‘unexplained anaemia of the elderly’.^198^

The prevalence of undernutrition, including deficiencies in iron, vitamin B12, folate and other micronutrients is rising among the elderly and is a leading cause of anaemia in this population. Older people may experience nutritional challenges, including low appetite and energy expenditure (“anorexia of ageing”) along with declines in biological and physiological changes such as diminished senses of smell and taste, loss of lean body mass, and changes in fluid and electrolyte regulation.^199^ In addition, age-related changes in gastrointestinal function, use of multiple medications, and social isolation can contribute to malnutrition, which in turn may contribute to anaemia.^197,199^ Blood loss, especially from the gastrointestinal tract due to a range of non-malignant conditions and gastrointestinal cancers, potentially exacerbated by oral anticoagulants, aspirin or other non-steroidal anti-inflammatory disorders is the most common and important cause of IDA in the elderly (appendix p. 31).^200^

Chronic inflammation may arise from existing comorbidities, such as rheumatoid arthritis or advanced cancers, infections and, to some extent, from ageing itself (“inflammaging”).^201^ Elderly people with chronic inflammation exhibit elevated hepcidin levels causing functional ID as discussed above.^202^ Impaired renal function and CKD impairs erythropoietin production, which can also drive anaemia. Further, in elderly men, age-related decline of testosterone levels can impair erythropoiesis. ^203^

Clonal haematopoiesis - subclinical somatic genetic changes in circulating leukocytes - is increasingly recognised as a cause of anaemia and is linked to increased risk of haematologic myeloid malignancies, such as myelodysplastic syndrome,^204^ and is also a risk factor for vascular disease.

Given the multifactorial aetiology of anaemia in the elderly, early and accurate diagnosis, along with effective management of both anaemia and its underlying causes, is complex. Significant evidence gaps remain. The characterisation of anaemia and its burden in older adults is limited, especially in LMICs, partly due to the lack of standardised diagnostic criteria and biomarkers to distinguish between different types of anaemia, such as anaemia of chronic disease, IDA, and unexplained anaemia. ^197^ Additionally, the biological mechanisms linking chronic conditions like inflammation and CKD to anaemia in older adults are not yet fully understood, and treatment options for unexplained anaemia remain limited.^205^

### Environmental considerations for anaemia

#### Impact of air pollution on anaemia

An increasing body of research has examined associations between air pollution (both ambient and household) and haemoglobin levels and risk of anaemia (appendix p. 32).^206^Exposures to air pollutants, such as fine particulate matter (PM_2.5_), were associated with reduced haemoglobin levels and increased risk of anaemia.(appendix p. 32).^207^ Importantly, the underlying mechanisms of these associations remain unclear. One suggested mechanism is that air pollutants can disrupt iron homeostasis by chelating or displacing iron from pivotal sites in the cell, which results in absolute or functional cell ID, which could contribute to increased risk of IDA with long-term exposure to air pollutants (appendix p. 32).^207^ In addition, air pollutants are potent oxidants and can generate reactive oxygen species (ROS), contributing to oxidative stress.^208^ Air pollution may also cause sterile inflammation^208^ or increase the risk of infection; this may trigger hepcidin, resulting in anaemia of inflammation.^209^

Exposure to carbon monoxide (CO), as a common component of air pollution, can lead to an increase of haemoglobin levels. CO binds to the sites in haemoglobin that normally bind to and carry oxygen, forming carboxyhaemoglobin (COHb), which reduces the oxygen-carrying capacity of haemoglobin (appendix p. 32).^210^ The elevated measured Hb levels caused by smoking might be mediated by exposure to CO.^211^ Consequently, WHO recommends adjustments to haemoglobin measurements based on smoking status and the number of cigarettes smoked per day.^12^

The contrasting impact of various air pollutants on haemoglobin levels increase the complexity of examining the overall effect of air pollution on anaemia. Future epidemiological and biological research is needed to understand how different air pollutants contribute to different types (e.g. IDA, anaemia caused by inflammation) and severities (mild, moderate and severe) of anaemia.

#### Impact of climate change on anaemia

Global heating has been projected to exacerbate the burden of childhood anaemia, mediated through factors such as malnutrition and malaria infection.^212^ Further work will be needed to validate these projections and begin to identify potential solutions. Limited research has evaluated the effects of climate change on the micronutrient content of food crops, agricultural production, and the food system (appendix p. 32).^213^ For instance, increased atmospheric CO_2_ concentrations could lead to lower iron concentrations in some highly consumed crops.^214^ This drop in iron supply could, in turn, increase the potential risk of ID.^215^

The impact of climate change on human body functions remains unclear. Heat stress could induce dehydration and electrolyte changes,^216^ and increase inflammation and oxidative stress,^217^ but the impact on iron homeostasis remains uncertain. Further studies are required to better understand the effects of climate change on human physiology and how that could, in turn, affect future global anaemia prevalence.

### Blood loss and anaemia in women of reproductive age

#### Heavy menstrual bleeding

Heavy menstrual bleeding (HMB) is defined as excessive menstrual blood loss that affects the physical, social, emotional or material quality of life.^218^ WRA with HMB are at risk of ID and anaemia (appendix p. 33).^219^ Using surveys implemented across two projects, a recent cross-sectional study in 10 low-income and middle-income cities found that 48·6% (95% CI 44·4–52·6) of women self-reported experiencing HMB.^219^

Key knowledge gaps remain in the context of HMB. While HMB has previously been defined as menstrual blood loss greater than 80mL per cycle,^220^ this definition is outdated and has been superseded by the more recent definition above. Improved tools for assessing heavy menstrual bleeding (HMB) status in LMIC are needed to enable the collection of better data on the prevalence of HMB and its impact on anaemia.

##### Interventions to reduce heavy menstrual blood loss

Drug therapy is the preferred initial treatment for those who want to maintain their reproductive function. These therapies fall into two categories: non-hormonal and hormonal interventions. Non-hormonal treatments consist of non-steroidal anti-inflammatory drugs (NSAIDs) and antifibrinolytic agents. Hormonal treatments include progestogens, combined oral contraceptive pills, and danazol.^221^

The antifibrinolytics, tranexamic acid (TXA) and mefenamic acid are effective first-line drugs used to treat heavy menstrual blood loss. TXA is the most used antifibrinolytic agent for reducing menstrual bleeding, and it reduces 40% to 50% of menstrual blood lost per menstrual cycle.^222^ NSAIDs, such as ibuprofen, mefenamic acid, naproxen, or aspirin, could reduce menstrual blood loss by 25% to 35% or more in approximately three-quarters of women with heavy menstrual blood loss.^221^ Mefenamic acid has been shown to reduce mean menstrual blood loss by 124 mL among women with HMB (95%CI: 61·6–186·4 mL/cycle; from 189 mL to 65 mL) in a study that objectively measured blood loss.^223^ Other effective first-line drug therapies include combined oral hormonal contraceptive methods, which contain oestrogen and progestin (appendix p. 33).^224^ Progestogen-releasing intrauterine systems such as the Levonorgestrel-releasing intrauterine system (LNG-IUS) are more effective for the treatment of heavy menstrual blood loss than oral medical treatment.^225^ Specifically, LNG-IUS demonstrated a reduction in menstrual blood loss with a mean difference of 66·91 mL (95% CI: 42·61 to 91·20 mL/cycle) when compared with other medical therapies, substantially lowering the mean menstrual blood loss from baseline (appendix p. 33).^225^

Addressing menstrual disorders is crucial to improving anaemia as it enhances iron reserves. Particularly in adolescents, utilising oral contraceptives as part of the treatment not only helps manage menstrual issues but also postpones pregnancy. This delay helps avoid the nutritional competition that can occur between a young woman and her developing foetus, allowing her more time to mature and potentially improving her nutritional status. Aside from assessing the effectiveness and safety of HMB treatments, future clinical trials can incorporate self-reported outcomes such as quality of life and participant satisfaction, include a cost-effectiveness component, and be conducted for a more extended treatment period.^222^ Global guidelines and comprehensive strategies that focus on reducing anaemia by addressing menstrual health have the potential to significantly enhance the quality of life for women, especially in LMICs.^226^

#### Postpartum haemorrhage

Postpartum haemorrhage (PPH) is typically defined as blood loss of 500 mL or more within 24 hours of birth. PPH impacts 14 million women globally and is a leading cause of maternal mortality worldwide.^227^ Antenatal anaemia can promote post-partum blood loss and increase the risk of PPH complications (appendix p. 33).^228^ Two mechanisms seem to be involved: a) women with anaemia cannot tolerate the same volume of postpartum bleeding as healthy women, and it can be life-threatening in mothers with clinically significant anaemia; b) antenatal anaemia may potentiate an increased heart rate and cardiac output, and reduce blood viscosity, increasing the risk of PPH.^228^ PPH also drives postpartum anaemia, which may impair preconception haemoglobin status for subsequent pregnancies (appendix p. 33).^229^

##### Interventions for postpartum haemorrhage

Preventative measures for PPH include anaemia-correction prior to delivery, uterotonics (oxytocin, heat-stable carbetocin, carbetocin, ergometrine, misoprostol) and TXA. Treatment measures include uterotonics (intravenous oxytocin) and TXA. While the benefits of iron supplementation in addressing maternal anaemia are well-documented,^230^ its role in reducing the risk of PPH remains unclear. A recent meta-analysis of a limited number of trials found that daily antenatal iron supplementation did not reduce the risk of PPH (risk ratio: 0·81, 95% CI: 0·49, 1·34; 2 trials).^230^

Intravenous TXA (given within three hours of giving birth) has been shown in a randomised trial to reduce maternal mortality when treating PPH.^231^ In response to this result, WHO updated the recommendation on TXA for PPH treatment in 2017.^232^ However, a recent multi-country clinical trial, administering TXA via slow intravenous injection within 15 minutes of the umbilical cord being cut or clamped, failed to reduce the risk of clinically diagnosed PPH in women with moderate or severe anaemia.^233^ Furthermore, a study demonstrated that early detection combined with a bundle of first-response treatment, including uterine massage, oxytocic drugs, tranexamic acid, intravenous fluids, examination, and escalation, reduced the risk of severe PPH, laparotomy for bleeding, or death from bleeding compared with usual care, although anaemia information was not collected in this trial.^234^ These findings underscore the need for further research, particularly in resource-limited settings, to better understand TXA’s efficacy, optimal administration of various interventions for preventing PPH, and their effect on anaemia.

Well-designed educational programmes for carers during childbirth, for example, midwives, nurses and trained birth attendants, that target behaviour change can improve healthcare workers’ knowledge, attitudes, and practices around PPH prevention and treatment with uterotonics.^235^ Specific studies on the availability and efficacy of various treatments including heat-stable carbetocin and TXA, education programmes, and how these interventions improve postpartum anaemia in women in LMICs are warranted.

#### Birth spacing for anaemia control

Birth spacing may be a complementary intervention to reduce anaemia in women and children, especially in settings where closely spaced pregnancies are common, and healthcare resources are limited. WHO recommends at least 24 months between a live birth and the next pregnancy to minimise health risks.^236^ High parity and short inter-birth intervals are associated with maternal depletion syndrome (MDS), a complex state characterised by poor maternal nutritional and health status during the reproductive cycle, which subsequently affects child health outcomes.^237^ Solutions that enhance birth spacing and reduce parity may enhance maternal iron and haemoglobin levels. Observational evidence on the specific effects of birth spacing on anaemia is limited. One study reported an increased risk of maternal anaemia among pregnant women with a birth interval shorter than two years.^238^ Additionally, a DHS survey across 28 Sub-Saharan African countries found that shorter birth intervals were associated with an increased risk of anaemia in children aged one to four years.^239^ Similarly, a large cross-sectional survey across 20 African countries found a modest positive association between longer birth intervals and reduced anaemia in preschool children, though this effect was only observed in girls.^240^ These findings highlight plausibility and a need for more research on the effects of birth spacing on maternal and child anaemia and the potential for increasing birth spacing as an intervention for anaemia control.

### Haemoglobinopathies and anaemia

Human adult haemoglobin is composed of two α- and two β-globin chains, encoded by the α- and β-globin genes, respectively.^241^ Over 300 mutations in the β-globin gene and 120 mutations in the α-globin gene cause various forms of haemoglobinopathies that lead to reduced synthesis of normal haemoglobin (e.g., α-thalassaemia, β-thalassaemia) or synthesis of structurally abnormal haemoglobin (e.g., HbS, HbE and HbC). ^242^ Carrier states for haemoglobinopathies are protective against severe malaria and originated in populations where malaria has been historically present and have accompanied their diaspora. They are present at varying frequencies and can contribute significantly to the global burden of anaemia via clinically significant homozygous forms and the more common, usually clinically silent carrier forms ^243^ (Table 3). In most cases, individuals with anaemia due to haemoglobin disorders will not respond to iron therapy and indeed this may be harmful.

Pregnant women with haemoglobinopathies are at risk of adverse maternal outcomes, including preeclampsia, heart failure, adverse birth outcomes and mortality.^244^ While haemoglobinopathies are associated with alterations in iron homeostasis, critical gaps remain in our understanding of the implications for placental iron transfer, infant iron endowment and risk of anaemia. Further research is needed to explore potential interactions of haemoglobinopathies with overlapping risk factors for anaemia (diet, micronutrient status) during pregnancy.

#### Homozygous (transfusion-dependent) thalassaemia

Transfusion-dependent thalassaemia poses a significant burden to health services due to its life-long chronic nature and severity. Haemoglobin Barts hydrops fetalis, the most severe form of α-thalassaemia, presents prenatally with foetal anaemia and hydrops. It is invariably fatal *in utero* without intrauterine transfusions.^245^

β-thalassaemia major, the homozygous form of β-thalassaemia, presents during infancy or early childhood with transfusion-dependent anaemia.^246^ Affected children and adults require lifelong regular 2–5-weekly blood transfusions; with a high risk of hepatic, cardiac and endocrine complications of iron overload necessitating iron chelation. Current research gaps for transfusion-dependent β-thalassaemia include optimising blood transfusion and iron chelation regimens, development of stem cell-derived blood products to replace donor-derived blood, inhibition of ineffective erythropoiesis (e.g. through ligand traps of TGF-β superfamily molecules, blocking SMAD2/3 signalling, hepcidin analogues, and inhibition of Janus kinase 2), and achieving cure through gene therapy (β-globin gene addition) or genome editing (upregulating γ-globin and foetal haemoglobin).^247^

#### Heterozygous thalassaemia (thalassaemia trait)

Heterozygous states of thalassaemia, though asymptomatic, contribute significantly to the global burden of anaemia.^248^ Both α- and β -thalassaemia traits results in hypochromic microcytic anaemia with haemoglobin concentrations in the low normal range, for example 100–130g/L.^246^ The combined prevalence of α- and β -thalassaemia trait is high (up to 50%) in some geographical locations, including parts of South and South East Asia and some parts of Africa, complicating the determinants of anaemia in these regions.

Individuals with thalassaemia trait have low red cell indices and are frequently erroneously diagnosed with IDA. This is confounded by the lack of widespread diagnostic facilities for thalassaemia trait. β-thalassaemia trait is accurately diagnosed by haemoglobin HPLC or electrophoresis demonstrating elevated HbA_2_. Diagnosing α-thalassaemia trait is more challenging as it necessitates technically demanding genetic testing.^242^ Establishing cheap and accurate point-of-care diagnostic assays for α-thalassaemia and improving the availability of haemoglobin HPLC and electrophoresis to diagnose β-thalassaemia are important priorities.

Although thalassaemia trait is considered an asymptomatic condition, evidence suggests it may be associated with increased erythropoiesis, increased soluble transferrin receptor levels, low hepcidin and high GDF15.^249^ Similarly, there is evidence that mild anaemia in thalassaemia trait could be significant, especially in states of high metabolic demand, for instance in pregnancy, affecting the offspring. ^250^ Defining the impact of thalassaemia trait on health and options for therapy is an unmet research need.

#### Structural variants of haemoglobin

Haemoglobin E (HbE), haemoglobin S (HbS) and haemoglobin C (HbC) are important structural variants of haemoglobin, each due to point mutations of a single nucleotide base of the β-globin gene. HbE, in both heterozygous (HbE trait) and homozygous (HbE disease) states results in mild asymptomatic anaemia, but may contribute to overall anaemia prevalence. However, the compound heterozygous state of HbE and β-thalassaemia has a variable phenotype ranging from mild anaemia to severe transfusion-dependent thalassaemia and is prevalent in South and South-East Asia and accounting for nearly half of the global burden of severe β-thalassaemia.^243^ HbS is a structurally abnormal haemoglobin polymerising in low oxygen conditions. The homozygous state, sickle cell anaemia, presents in childhood with recurrent vaso-occlusive episodes and mild to moderate anaemia.^251^ A similar phenotype is seen when HbS is coinherited with β-thalassaemia trait. Although sickle cell trait is generally asymptomatic, clinical features of sickle cell disease can appear under certain metabolic or environmental conditions (e.g., hypoxia and dehydration). However, sickle cell trait does not lead to anaemia and is not a significant contributor to the overall burden of anaemia.

HbC, caused by the substitution of glutamate by lysine in the sixth position of the β-globin chain, is a less soluble form of haemoglobin than HbA. HbC trait (heterozygous state) is clinically silent. Homozygous HbC disease (HbCC) results in mild chronic haemolytic anaemia with splenomegaly. The compound heterozygous state of HbC and HbS (HbSC) presents with clinical features of sickle cell disease.

#### Safety of iron interventions in areas with high prevalence of haemoglobinopathies

In regions with a high prevalence of haemoglobinopathies such as β-thalassemia, sickle cell disease, HbH disease, or HbE β-thalassemia, the safety of iron interventions requires careful consideration due to the complex interplay between iron metabolism and haemoglobin disorders. ^252^ Mutations in the haemochromatosis gene *HFE* and other haemoglobinopathies ^253^ that increase the risk of iron overload are quite rare^254^. However, it is important to note that nutritional interventions that increase iron intake (e.g. blanket iron supplementation programmes) may be harmful to individuals, including carriers of haemoglobinopathies, as they may be unable to fully downregulate iron absorption in the presence of iron repletion. In ideal circumstances, individuals with severe anaemia should be assessed for underlying causes of anaemia (e.g., haemoglobinopathies or infection) before undertaking any nutritional treatment or therapy.

### Achieving public health impact through effective anaemia interventions

**Figure 7:**
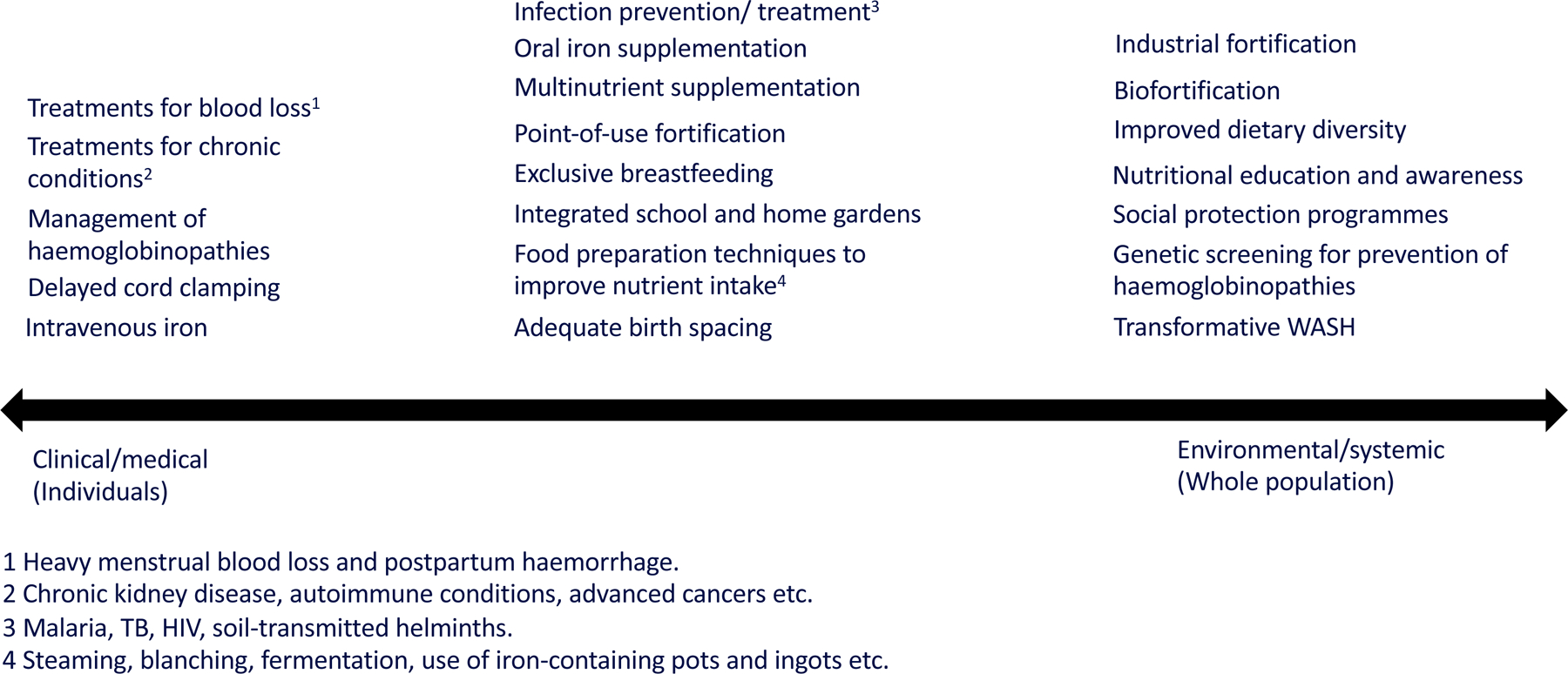
Overview of current interventions addressing anaemia. On the left are clinical interventions targeting individuals; in the middle are interventions aimed at vulnerable sub-populations, such as women of reproductive age, young children, or those in high-burden infection settings; and on the right are systemic or environmental interventions designed to benefit whole populations.

The key knowledge gaps and recommendations related to anaemia aetiology and management outlined in this section are summarised in Table 4. The objectives of public health anaemia interventions (Figure 7) should be context-specific and align with other health interventions in terms of their efficacy, safety and cost-effectiveness in improving functional health outcomes within a reasonable time frame. Priority should be given to selected evidence-based interventions and the creation of a minimum set of conditions that is necessary for these interventions to take hold and be sustained. This includes considering factors such as feasibility, availability of resources and patient or consumer preferences. In the next section, we will discuss aspects of integration of anaemia interventions into programmes.

## Section 3: Improving anaemia control programs: implementation and governance.

Despite decades of investments and global research, anaemia remains a pressing global public health problem. Implementation of anaemia control programmes at a large scale requires detailed baseline epidemiologic assessments, understanding the aetiology of anaemia, pilot testing of interventions, crafting and resourcing of policies and programmes tailored to country and population needs, delivery of a comprehensive range of interventions with high fidelity to target all populations, prioritising equitable access, and ongoing monitoring to assess safety, effectiveness and equity. This section aims to answer the following questions: 1) what are the enablers and barriers to implementation and equitable access to anaemia prevention, control, and treatment measures?; 2) which actors must govern, fund, and advocate for anaemia in health and nutrition agenda-setting and policy, and what are the best mechanisms for governance at global, national, and sub-national levels?; and 3) what policy and governance instruments can drive action on anaemia? We propose an integrated global policy and governance approach, highlighting mechanisms that can be adopted at global, national, and local levels. Understanding the challenges of implementation, including governance and financing, is paramount to accelerate progress towards achieving the anaemia-specific global Sustainable Development Goals (SDGs), the World Health Assembly (WHA) Global Nutrition targets, and for any future post-2030 goals.

### Current state of anaemia governance and implementation

Although many global nutrition issues, such as stunting and wasting, have received increased attention and prominence in global policy negotiations over the last two decades, anaemia has not received comparable prioritisation in the policy agendas of major economic powers or key bilateral and multilateral institutions. Despite the established association with economic indicators such as educational attainment and productivity, ^255^ anaemia prevention and management has not been prioritised by governments. The estimated economic return in productivity gains from investing US$8 billion in targeted interventions to reduce the global anaemia burden among women by 50% is US$66–110 billion.^256^ However, according to recent donor disbursement estimates, global anaemia financing, which supports food-based (i.e., staple food fortification) and non-food interventions (i.e. iron supplements, multiple micronutrient supplementation and powders, malaria control), declined to US$116 million in 2022 from US$152 million in 2021.^257^

In addition to these limited donor disbursements, global anaemia governance has suffered from fragmentation and political inertia. The complex architecture of actors across sectors including agriculture, health, social protection, water, hygiene, and sanitation, as well as across levels of government has resulted in asymmetries and a diffusion of power, limiting the reach, effectiveness and efficiency of core services to prevent and treat anaemia. Now, more than ever, there is an urgent need to reconstruct power and governance for a more purposeful, systematic, integrated, transparent and accountable collaboration that leverages the collective strengths of all relevant parties,^258^ including multi- and bi-lateral agencies, governments, civil society, and the private sector to effectively help countries accelerate progress on anaemia-related SDGs (Appendix Table 9 on p. 19) and WHA targets (Section 4), as well as future sustainable goals post 2030.

### Core Principles for effective, prioritised, and high-quality implementation of anaemia solutions

To facilitate the development and delivery of more equitable, sustainable and evidence-informed anaemia policies and programmes, we outline four core interrelated principles that can guide prioritisation, implementation, monitoring and evaluation in the political economy of anaemia. These principles draw on frameworks by Shiffman and Smith,^259^ which examine why some global health initiatives receive political priority over others, and the WHO’s framework for accelerating anaemia reduction’.^5^ Within the WHO framework, five action areas are identified, including prioritising key preventive and therapeutic interventions and optimising service delivery across platforms and sectors. In adapting these frameworks, we describe factors shaping implementation and challenges experienced in global initiatives for anaemia control which may impair progress in reaching national and global targets (Table 5).

#### Core Principle 1: Leverage existing data and improve infrastructure and resources to collect new data

The complexity and dynamic nature of anaemia necessitates continual attention to sociopolitical, geographical, environmental, and cultural contexts. Contextual conditions drive and inform the development, adaptation and implementation of evidence-based interventions (nutrition-specific and nutrition-sensitive) for populations. Unpacking context requires two activities: 1. measuring the prevalence and aetiology of anaemia within the population or across settings and 2. considering the enabling environment which influences implementation across levels of a socio-ecological framework (individual, interpersonal, organisational, community, policy).^260^

We have discussed challenges in anaemia surveillance and suggested approaches to strengthening the anaemia data landscape in Section 1 (also see Box 3). Multi-sectoral efforts are needed to efficiently collect good-quality data to inform policies and programmes. In addition, understanding barriers and facilitators to implementation strategies is critical. A comprehensive assessment (formative research) should account for historical, cultural, social, and systemic contextual factors that shape implementation, including colonialism and racism (e.g. inequitable practices and policies), institutional and providers’ biases (e.g. gender-related inequities), while also identifying community strengths and assets to enhance implementation efforts.^260^ For example, gender-based social norms can influence the quality and quantity of food allocated to women and children in social protection schemes, as well as women’s freedom of movement and time allocation to visit health centres and markets to obtain antenatal care, family planning, prenatal iron and folic acid (IFA) supplements and healthy and diverse diets (appendix p. 34).^261^A landscape of potential platforms where target populations might best be reached (e.g. adolescent girls through schools or mothers through prenatal visits) is also important information for planning of implementation and sustainability of interventions.

#### Core Principle 2: Catalyse multi-sectoral governance and engagement

To ensure continuity in implementation and maximise impact at scale, national strategies and plans are best developed through collaborations between sectors, levels of government, and communities of practice (e.g., practitioners, researchers, community-based organisations, and the affected population) (appendix p. 34).^262^ Multi-sectoral governance and engagement in anaemia prevention and control strategies is necessary due to the complex aetiology of anaemia and the need for long-term commitments to support anaemia as a political priority, despite a change of government, and to improve the coordination of monitoring and evaluation mechanisms. More importantly, the co-production of an anaemia strategy allows for policy coherence (or horizontal coordination) through the adoption of a ‘Health in All Policies’ approach.^258^ This equity-centred collaborative approach recognises that health is created by a multitude of factors and sectors, and in many cases, beyond the scope of traditional public health. By integrating health into policymaking processes across sectors, the co-benefits become increasingly apparent. For example, the societal value of win-win interventions with impacts on multiple development goals can stimulate co-financing and co-benefits and ensure the total multi-sectoral value of these interventions is captured, and sectors’ shared goals are achieved more efficiently.

Engagement of multi-sectoral relevant parties does not stop with the approval of a national strategy and budget. It is crucial to consider what type of governance mechanisms or structures will be required to ensure that the coordination of activities and objectives of the strategy can be put into practice and monitored and evaluated to determine whether the desired population health and health equity impacts are being achieved. Therefore, a multi-sectoral anaemia strategy needs to outline which body at the central level is responsible for overall coordination and oversight of the programme or policy; how this body will be managed, financed and governed, especially at local levels; and specific roles, responsibilities, and accountability mechanisms for other relevant bodies.

#### Core Principle 3: Integrate prioritised interventions into implementation plans

Effective implementation requires knowledge of which intervention, or package of interventions, is effective, context-appropriate and should be prioritised.^263^ This selection is largely informed by data and context (Core Principle 1). Coordination is required from the onset across multiple sectors and even within agencies in a country to plan, integrate, implement, and monitor interventions to track progress towards comprehensive efforts to better reduce anaemia. Systematic monitoring allows adjustment based on what works in specific contexts. Institutional strengthening and capacity-building efforts are paramount, requiring an assessment of resources and technical capacity at leadership and implementing levels.

#### Core Principle 4: Centre social justice and human rights

Health inequities pose ethical challenges for progress on global health issues^264^ For anaemia, disparities begin with a lack of contextualised data. A review of nutrition data found that the largest data gap was related to biomarkers of micronutrient status for women.^265^ This inequity can be compounded by factors such as age, sex, race, geography and family income.^266^. For instance, while the overall anaemia prevalence among individuals aged two years and older in the U.S. is relatively low (9·4%,) the prevalence among African American women is 31·4%.^267^

Taking a rights-based approach to anaemia means everyone is entitled to have available, accessible, acceptable and quality health care, while also addressing the underlying determinants of health that extend beyond health services, goods, and facilities.^268^ It requires laws and policies addressing the social determinants of health for women and girls, who are at highest risk of anaemia. This includes promoting gender equity in education and information communication technology, as educational status and media exposure are factors associated with anaemia in WRA across LMICs.^269^As noted under Core Principle 1, attention is also required to increasing women and girls’ agency and transforming social norms that may constrain their access to nutritious diets and health services. Rights-holders should be involved in all stages of policies and programmes, in line with the human rights principles of participation and accountability. This can ensure that policies and interventions are being enforced, and rights are being upheld. Women and girls should be supported in this effort to hold governments accountable on their obligations.

### Global governance for anaemia

#### Current global governance architecture

Research emphasises the need for a global action plan and collaborative implementation to build sustained global political commitment and financing for interventions to be effective (appendix p. 34).^256^ To actualise the core principles at the global level requires a strategic vision, an understanding of the actors involved in anaemia governance and diplomacy, mechanisms for performance and accountability and ensuring fairness.

WHO’s role in addressing anaemia is to set global guidance, framework, norms and standards, provide technical support, raise awareness, and monitor data to track progress. The WHO Comprehensive framework for action to accelerate anaemia reduction outlines five action areas for member states, civil society, academia and other non-governmental organisations (e.g., professional associations and societies).^5^ These include: analyse data on causes and risk factors for anaemia; prioritise key preventive and therapeutic interventions; optimise service delivery across platforms and sectors; strengthen leadership, coordination, and governance at all levels; and expand research, learning and innovation.^5^ While the work they have undertaken is critically important, there is still much more work to undertake by WHO and partners, with more significant investment and support for the agency.

Anaemia is highlighted in other global strategies such as The Global Strategy for Women’s, Children’s and Adolescents’ Health (2016–2030) and The Global Action Plan for Healthy Lives and Well-being for All (SDG3 GAP). Under the SDG3 GAP, agencies commit to aligning their ways of working to provide more streamlined support to countries and reduce inefficiencies. For anaemia, SDG3 GAP has also contributed to greater alignment in the multilateral system by integrating elements of the Every Woman, Every Child initiative to support the closer integration of sexual and reproductive health and rights, and women’s, maternal, newborn and adolescent health into primary health care. This is particularly important as women’s issues have historically received little attention and inequitable financing (appendix p. 34).^270^ In addition to this, WHO and UNICEF established the Anaemia Action Alliance to foster accelerated and coordinated efforts to reduce anaemia, including the implementation of the comprehensive framework through core and working groups. The Alliance is primarily composed of multilateral institutions (e.g., UN agencies and other intergovernmental organisations), bilateral development agencies and other non-state actors, including non-governmental organisations and philanthropic foundations. While the Alliance is still in its infancy, global governance and diplomacy for anaemia cannot be stressed enough as we move towards the post-SDG era.

Global governance of anaemia faces challenges including conflict in values, ideologies and norms, as well as increased fragmentation, competition for financing, and access to and influence over policy agendas.^271^ Power asymmetries exist between public and private actors within and outside of health, and result in the dominance and influence of some actors over others (appendix p. 34).^272^ Strengthening governance mechanisms is crucial for improving policy coherence, collective decision-making, and action across sectors to address health issues such as anaemia effectively.^273^ To be effective, these mechanisms need to be equity-driven, evidence-informed, adaptable, resilient, scalable and sustainable.

#### Global finance mechanisms

While there are disparate priorities across the health sector, siloing issues such as anaemia is counterproductive from a financing perspective. In recent years, a number of innovative financing tools have been launched to spur greater investment in global health challenges.^274^ These tools aim to track and leverage investments across public, private and philanthropic sectors to address financing gaps at various parts of the development pipeline. For example, the international community has called for a coherent, comparable and unified system to capture resources flowing into developing countries for their sustainable development.^274^ In response, the Organisation for Economic Co-operation and Development (OECD) developed the Total Official Support for Sustainable Development in 2017 to monitor multilateral and bilateral spending and inform evidence-based policy discussions.^274^ While an important step, disaggregated tracking of investments for nutrition, and specifically anaemia, remains limited.

Beyond financial tracking, global collaboration and appropriately structured financing mechanisms are needed. The Global Financing Facility (GFF), the funding arm of Every Woman Every Child, was launched in 2015 to address funding gaps in 63 target countries by supporting country-led efforts to build equitable and resilient health systems and promote long-term sustainable financing.^275^ For nutrition, GFF accelerates the implementation of high-impact, evidence-based direct and indirect nutrition interventions across sectors through innovative financing instruments including increasing domestic resource mobilisation, increasing and better aligning external financing, and leveraging private-sector resources.^275^ As of 2021, GFF has provided US$186 million in grant financing to scale-up nutrition interventions across 22 countries, though it is unclear how much has been allocated to anaemia. Other innovative financing partnerships include Power of Nutrition ^276^ and Stronger Foundations for Nutrition ^276^ which bring together a diverse network of donors, implementing partners, and national governments to mobilise more financing to transform global nutrition together.

However, despite these emerging mechanisms, nutrition financing remains fragile, especially for anaemia surveillance, data systems, and program evaluation. This vulnerability is further exacerbated by shifting political agendas and tightening budget in major donor countries, including the United States (also see Box 1).

#### Global monitoring and equitable target-setting for anaemia reduction

WHO is well positioned to continue the global monitoring of anaemia because of its constitutional mandate, its accountability to member states, its ability to mobilise global expertise, and its unique position to generate productive interactions with country information systems. Still, civil society, academia, international and local non-governmental organisations also have a role to play in holding governments accountable and ensuring actions are implemented at national and local levels.

There is much debate whether global target-setting is equitable, including current anaemia targets. Despite the aspirations of the SDGs, global targets do not adjust for factors such as country income, geopolitical tensions and the aetiology of health problems that might inhibit or support progress. Using the SDGs to monitor progress and classify countries as on or off track, while intuitive, may be misleading because the numerical targets are based on time trends, without attention to what could be reasonably achieved by each country given their available resources. Countries that are off track can be excused from attaining targets that were unachievable in the first place and LMIC policymakers can question the relevance of targets that were set without regard to resources and challenges. Therefore, we propose that particular attention should be paid to equitable and achievable target-setting for anaemia as we move towards the end of the SDG era. ^277^ In Section 4, we will propose a new national-tailored approach for setting future targets.

### National government-led anaemia strategies

#### Building and sustaining political commitment for anaemia reduction

Political will is foundational for advancing anaemia reduction strategies and ensuring these efforts translate into action.^278^ Generating political momentum requires visibility of the issue and demonstrable evidence that the problem can be effectively addressed. However, anaemia often remains invisible due to lack of quantification of anaemia-associated morbidity and mortality, and significant gaps in data and knowledge, as well as the lack of attention to women’s health issues.^2,265^

However, evidence alone is insufficient without a supportive political economy. National consensus on the main drivers and causes of anaemia and the corresponding policy responses is crucial. Historically, anaemia reduction strategies have focused only on iron delivery, but this approach alone is insufficient to address the many underlying causes of anaemia beyond dietary iron deficiency.^2^ Instead, multi-sectoral strategies addressing context-specific anaemia causes are needed, requiring engagement and commitment from policymakers across ministries beyond health, such as agriculture and the environment. Multi-sectoral integration in planning and action has been key to stunting reduction and is likely equally important for anaemia reduction.^279^ Thus, it is critical to engage and hold to account the multiple actors through some type of coordinating mechanism (see below and Core Principle 3).

National ownership of the problem is crucial (appendix p. 35).^280^ For example, an analysis of the 60 member countries of the Scaling Up Nutrition movement found that higher operational and embedded commitment for nutrition was associated with lower prevalence of anaemia in WRA.^281^ Operational commitment includes resource allocation and effective coordination mechanisms, while embedded commitment additionally includes a commitment to actions that indirectly influence nutrition (e.g., poverty reduction). However, competing health and nutrition priorities exist, with prioritisation often based on the power of the actors involved, the ideas they use to portray the issue, the nature of the political contexts in which they operate, and the characteristics of the issue itself.^259^ Strong leadership, including anaemia “champions” at the national and donor level, may be needed to achieve and maintain commitment to anaemia reduction. Various actions related to actor accountability and garnering champions can promote anaemia ownership and prioritisation.

#### Developing a national strategy for anaemia reduction

National health policies, strategies and plans (NHPSPs) play an essential role in defining a country’s vision, policy directions and strategies for ensuring the health of its population. NHPSPs provide a framework in almost every country for dealing with the complex range of issues needed to improve health and health equity outcomes, including those related to the SDGs. However, the development of NHPSPs is a complex and dynamic process, with the precise nature of developing NHPSPs varying from country to country according to the prevailing political, historical and socio-economic climate.

In developing a standalone national strategy for anaemia reduction, it is first essential to assess the prevalence and distribution of anaemia, the existence and effectiveness of current policies and programmes, the availability of institutional resources, and the existing institutional and human capacity to implement interventions. Once the landscape has been analysed, a coordination mechanism or task force can be established to advocate for and spur the drafting of a national strategy for anaemia reduction to build prioritisation, ownership, and commitment. The strategy should specify goals and specific time-bound objectives, the interventions to be implemented vertically (between the national and various subnational levels) and horizontally (across sectors within national and subnational levels), and the mechanisms by which different government and non-government institutions will coordinate efforts in an integrated programme. Once the strategy is developed, protocols, guidelines, and training materials should be updated to align with the national strategy and context. If the political context and capacity are restrictive, better integration of anaemia into the wider national health strategy should be prioritised.

#### Establishing structures for coordination, collaboration, monitoring, and accountability

Prevention, control, and treatment of anaemia require a coordinated response that involves many institutions that bear some responsibility.^282^ Multi-sectoral and disciplinary action is required through collaboration and sharing of roles and responsibilities, most predominantly across health and agriculture ministries with substantive engagement with international and local non-government organisations (NGOs), civil society, private sector actors and UN/bilateral agencies. Health and agriculture ministries should create a platform that engages other ministries involved in social protection, environment and climate, child and women welfare and education, as some examples.

There are various examples of multi-sectoral action for anaemia. Within Uganda’s National Anaemia Policy, the government established a National Anaemia Working Group in which multiple sectors, including agriculture, health, and education engaged. This group coordinated all anaemia-related activities which included an operational plan, data management, and training of workers across the country. Similar coordinating mechanisms have occurred in the Philippines and Sierra Leone. In both Sierra Leone and Uganda, there were positive lessons in that coordination allowed for more cohesive structures to prioritise and align activities. These bodies also brought together organisations that normally would not work together, creating better coordination and trans-sectoral collaboration. The last lesson is that coordinating bodies serve as a mechanism to hold the various actors accountable through monitoring activities at the sub-national level.^283^

#### Operationalisation and continuity to sub-national planning efforts

There is a critical need for better mechanisms to link national and sub-national efforts and actors involved in anaemia programming across sectors to enhance the effectiveness of prioritised interventions as well as their monitoring and evaluation. A multi-country analysis of political and institutional determinants of delivering a multi-sectoral nutrition response noted that local actors are motivated to implement national policies when given direct responsibility and commensurate funding for implementation.^284^ At sub-national levels, health systems depend on the capacity of local or regional decision-makers, including their skills and knowledge and their ability to engage with local communities to ensure decisions reflect their needs and priorities. However, there are several barriers to local ownership and accountability. First, because sub-national data on anaemia is not routinely collected, the burden, contextual factors and effect of investments on improving intervention coverage cannot be accurately measured, especially as it relates to geographical equity gaps.^285^ Second, local political party turnover can disrupt programmes or the planning of forthcoming programmes if priorities change for the incoming party.^286^ Therefore, it is necessary to garner community and civil society support to ensure continuity. Third, the extent to which power and responsibilities for anaemia prevention and management are centralised versus decentralised influences how anaemia interventions are integrated or coordinated with other healthcare services within the NHPSP, particularly to reduce overburdening staff, costs and potential worsening of health inequities.^287^ Specific efforts may be needed to overcome these barriers and identify incentives to coordinate. Recognising that the drivers of anaemia are diverse, we provide two examples of successful country implementation that lend invaluable learnings for replicating and accelerating anaemia reductions in other contexts (Box 4).

#### Mobilising domestic resources and financial flows

Health financing encompasses mobilisation, accumulation, and efficient and effective allocation of funds to cover the health needs of the people, individually and collectively.^288^ It ensures funding is available and sets the right financial incentives to providers, to ensure all individuals can access effective, essential, high-quality health services. In low- and middle-income countries, financing schemes include domestic funding from central and local governments, out-of-pocket household expenditures, non-governmental organisations (including religious organisations and local philanthropies), private companies, and development assistance. While depending greatly on country context, domestic investments are key for country ownership and funding that is predictable, reliable, and sustainable, building system health and nutrition resilience in the medium and long-term. This is particularly important for global health issues such as anaemia where change is gradual, and social and economic benefits often lag behind initial investments. Like the development of the overall strategy, financing should engage multiple stakeholders to avoid redundancy, but also ensure interventions are appropriately resourced given some interventions carry significant costs. Importantly, linking financing to human resources is critical for implementation, monitoring and evaluation.^289^ A health workforce that is equipped with the knowledge, skills, and tools to deliver anaemia interventions is essential for providing effective coverage that can deliver on the nutrition-related SDGs. However, as evidenced in the policy review and similar reviews, limited information is available on how much governments provide in support of health worker capacity and training.^290^

In addition, leveraging quantitative tools such as the World Bank Optima Nutrition and the Micronutrient Intervention Modeling Project (MINIMOD) can provide practical advice to governments to assist with the allocation of current or projected budgets across nutrition programmes. These geospatial models can determine funding allocations that minimise anaemia at both the national and regional levels using a portfolio of interventions including micronutrient supplementation programmes, infant and young child feeding education, treatment of severe acute malnutrition, treatment and prevention of diarrhoea, fortification of foods, family planning, and malaria prevention interventions.^291^

## Section 4: A better way forward: redefining future anaemia reduction targets

In 2012, the global community pledged to halve the prevalence of anaemia in WRA, as one of WHO’s Global Nutrition Targets (GNTs).^292^ This pledge was reaffirmed in 2015 under the SDG framework, with a 2030 deadline ^293^ It is now clear that most countries will fail to meet this target (appendix p. 36).^6^

Once the 2030 deadline has passed, new global targets for anaemia reduction should be set.^294^ This section critically evaluates the process by which the 2030 anaemia targets were set and introduces a tailored, evidence-based methodology for setting ambitious but realistic targets for future anaemia reduction efforts.

### International development goals

Sets of international development goals like the SDGs and GNTs aim to define specific, time-bound targets to address global challenges such as poverty, health inequities, and environmental sustainability. Since the 1960s, the international community has repeatedly established such goals ^295^. For instance, during the UN’s First Development Decade (1960–1970), all countries pledged to grow their aggregate national income by more than 5 per cent per year. By the Third Development Decade (1981–1990), countries also pledged to lower their infant mortality rates to below 120 per 10,000 live births.^296^

While early international development goals focused primarily on economic development, they have become more ambitious and diverse over time. The SDGs (2015–2030) comprise 17 interconnected objectives intended to guide the international community towards a more equitable and sustainable future, with 169 specific targets for different dimensions of social health, economic, and institutional improvement. Parallel, overlapping goals have been set by other international institutions, including WHO’s GNTs (2012–2025),^292^ the OECD’s seven International Development Goals (appendix p. 36),^297^ and the Food and Agriculture Organisation’s Rome Declaration on Food Security (1996–2015).^298^

### The purpose of international target-setting

International development goals define specific quantitative targets.^299^ It is, therefore, tempting to interpret them in a purely literal manner: as a commitment to improve particular metrics of development by a given amount.^300^ Moreover, because these targets are stated as thresholds, a country either succeeds or fails, depending on whether it meets this quantitative threshold or not.

Targets should be ambitious to reflect the tone and intent of international development goals: to inspire governments, non-government organisations, donors, industry, and civil society to move beyond business-as-usual and deliver transformative change. This ambition is reflected in the absolutist language that the SDGs often employ in their overarching goals. For example: No Poverty (Goal 1), Zero Hunger (Goal 2), and Gender Equality (Goal 5). However, we propose that the more specific, quantitative targets should be achievable for all signatories as well as ambitious. Unattainable targets may reduce motivation rather than increase it, and failure to reach targets may attract unwarranted criticism rather than support.^301^ Moreover, unachievable targets could distort the allocation of limited national and international resources and undermine the credibility of both the individual targets and the overall development programme.^302^

Achieving quantitative targets is the literal and explicit purpose of setting international development goals, and countries are likely to be judged by whether they reach them. It would therefore be helpful if a rational and defensible process is used to set the targets. However, if we assume that the only reason to set international development targets is to have them met, we overlook a range of ancillary benefits that targets are also intended to deliver. We recognise four implicit motivations for setting development targets: 1) Setting a Collective Vision. Targets aim to unify governments, donors, NGOs, and civil society in pursuit of transformative change. ^297^ Ambitious, even absolutist goals set the tone for significant advancements; 2) Encouraging Equity and Solidarity. Development goals foster a collective vision, emphasising global solidarity and shared responsibility;^293^ 3) Focusing Attention. Setting specific targets shines a spotlight on neglected dimensions of development, such as anaemia, which receive less attention and funding relative to their burden^303^ and 3) Standardising Measurement. Quantifiable targets drive the development of reliable data collection, monitoring, and reporting systems, essential for tracking progress and identifying gaps (appendix p. 36).^304^

Each of these implicit motivations can drive international development and be achieved without necessarily making material progress towards the development target itself.

Both the explicit purpose and implicit motivations of international development goals should shape how future targets for diseases and conditions like anaemia are framed and chosen. This involves balancing competing priorities and ideas. For example, an ambitious global target may be more likely to attract attention and increased funding to important issues, but a modest target is more likely to be achieved.^305^ Targets should also be quantifiable by readily available indicators ^306^ that may miss the complexity of a disease but are straightforward to collect by all countries, regardless of capacity (appendix p. 36).^307^ Keeping considerations such as these in mind, this section reflects on the existing 2030 anaemia targets and proposes revisions to the target-setting process.

### Global anaemia targets

Despite the consistent focus of international development goals on hunger and malnutrition, anaemia has only been included as an explicit target since 2012, when the condition was included as one of the GNTs.^292^ In 2015, the SDGs also adopted this anaemia target as indicator 2·2·3 of “Goal 2: Zero Hunger”. Both goals pledged to halve the overall prevalence of anaemia in WRA by 2030.

Before we assess international progress towards this target, it is worth dissecting the anatomy of this target in detail. To begin with, the target population is WRA (pregnant and non-pregnant women). This subpopulation is particularly impacted by anaemia; prevalence and severity are high,^35^ complications are more common, and the consequences for foetal development and child health produce a multiplier effect.^308^ Secondly, anaemia is typically classified as mild, moderate, or severe, based on haemoglobin concentration.^309^ Both global targets for anaemia focus on the overall prevalence of anaemia – the sum of the prevalence of all three severity categories – which is a common and established measurement of the condition. Thirdly, the SDGs and GNTs both aim to halve the overall prevalence of anaemia, compared with a baseline set during the period 1993–2005.^310^ This 50% target was chosen based on the progress of “exemplar” countries that have achieved exceptionally large and sustained reductions in anaemia. These include China (a 4·2% annual rate of reduction between 1981–2002), Nepal (a 7·8% annual rate of reduction between 1981–2002), and Guatemala (a 7·6% annual rate of reduction between 1981–2002).^310^ Finally, both sets of international development goals aim for the same 50% reduction in anaemia in all signatory countries. This international uniformity creates a target that is equal, compelling, and easy to communicate.^306^

The global targets for anaemia have been in place for more than a decade, and so these four dimensions of the target are now familiar and widely accepted. However, during the GNT setting process for anaemia, each was debated, and alternatives were considered.^310,311^ For example, while WRA are the most vulnerable subpopulation to anaemia, the inclusion of other vulnerable groups – particularly children – was also considered.

Three key limitations of choices made in setting the global anaemia target have been identified: First, the international uniformity of the anaemia target is perhaps its most striking characteristic. A country’s capacity to reduce anaemia prevalence depends on a range of political, economic, demographic factors, and local determinants of anaemia, which vary considerably between countries. Different countries are also likely to place different priorities on anaemia based on national preferences and competing demands on limited health resources. Thus, while a uniform international target provides a shared, nominally egalitarian benchmark for progress and accountability, it ignores meaningful differences between countries. The SDGs framework explicitly encourages countries to set national targets that take into account unique national circumstances,^293,310^ but we could not identify any countries that set individualised targets.

Second is the focus on overall prevalence. Overall prevalence is a simple indicator of anaemia. It has a long history as a metric in anaemia epidemiology and is straightforward to measure and communicate. However, the burden of a disease or condition is based on both prevalence and severity, typically measured using a disability weight (DW) ranging from 0 (reflecting perfect health) to 1 (reflecting death). Severe anaemia carries a disability weight that is an order of magnitude higher than either moderate or mild anaemia (DW = 0·149 compared with 0·052 and 0·004 respectively), ^312^ and carries a substantial probability of longer hospitalisation and mortality (appendix p. 36).^145^ Measuring overall prevalence also ignores health gains associated with reductions in severity of anaemia. Interventions that reduce the severity of anaemia from severe to moderate, or from moderate to mild, will deliver large improvements in well-being, but are not recognised as a reduction by the metric of overall prevalence.

Third is the unrealistic magnitude of the target. The 50% reduction targets for overall anaemia are ambitious and would represent an enormous improvement in well-being. However, countries must deliver rapid and sustained reductions to reach this threshold – a particular challenge for countries where large proportions of the population are anaemic. The 50% threshold was calculated by considering the previous progress of “exemplar” countries.^311^ However, expecting every other nation to mirror these decline rates is unrealistic; even the exemplar countries themselves have been unable to reproduce them during the SDGs period (Figure 1). In Malawi, for example, a 50% reduction would require 2·8 million women to become non-anaemic out of a population of 9·3 million WRA.^2^ Countries that invested significantly in anaemia reduction prior to the baseline period might have found that further improvements have had diminishing returns,^313^ greatly affecting their ability to reach the proposed 50% reduction.

As described in the introduction, the latest modelled evidence ^2,8^ strongly suggests that most countries are not achieving the rate of reduction in anaemia prevalence that would be needed to meet the 2030 deadline (Figure 1). This conclusion is not a surprise: it matches previous analyses of international progress on anaemia,^5^ and has been highlighted by WHO reporting.^7^ It also mirrors the slow progress towards almost all of the 17 goals.^314^ However, it is slow even by comparison, with SDG Target Indicator 2·2·3 proceeding at less than half the pace of comparable SDGs targets in nutrition (appendix p. 36).^5^

Despite this lack of progress, a new and revised set of targets for anaemia is likely to follow the era of the SDGs and GNTs. Keeping in mind both the explicit purpose of international targets to guide progress, their implicit motivations, and the limitations identified with the current target structure, we propose an alternative methodology for setting international development targets that remain ambitious but are more likely to be achievable.

### A new approach to setting targets

#### Tailored national targets

Our first proposed change to the anaemia target is its uniform nature. Different nations face a unique set of circumstances and challenges which constrain their ability to reduce anaemia prevalence, even with commitment and investment. For any country, measurably achievable progress depends on a set of contextual factors that will affect the relative cost and effectiveness of public health interventions. Progress reflects the current rates and severities of anaemia and its causal drivers, including the age and sex distributions of the population, environmental covariates such as endemic malaria, and social and economic factors, including the current coverage of important interventions.^2,11^ Furthermore, different countries have different pressing health priorities competing for limited budgets, and the opportunity costs of investing in reducing anaemia prevalence need to be carefully considered.

If we accept that countries face different anaemia challenges, and differ in their capacity to address them, then it follows that any uniform target will fail to deliver the combination of ambition and achievability that create an effective development target. If we uniformly set expectations ambitiously high, then countries with lower capacity or more challenging circumstances will be unable to achieve them. Conversely, if our reduction target is modest enough for all countries to achieve, then it will not represent an ambitious goal for countries with higher capacity. A modest target would neither focus attention on the global burden of anaemia, nor create impetus to address it.

Tailored national targets would satisfy the competing demands of ambition and achievability and would remain faithful to the spirit of international development goals which acknowledge the importance of local autonomy. However, separate targets for each country would fail to deliver on the primary implicit aim of international development goals: a unified collective vision for global development. The current uniform target for anaemia is simple, easy-to-communicate, and equitable. These attributes will be lost if it is replaced by 193 separate, nationally tailored targets.

We therefore propose that national targets be determined, but communicated as a single aggregate global target, created by an unweighted average across countries. This approach creates a single ambitious global goal for anaemia reduction that is nonetheless defensibly based on achievable national targets. It mirrors the nested structure of the SDGs as a whole, where the overarching goal (e.g., Zero Hunger) communicates a clear vision, but where the multitude of associated targets and indicators (e.g., anaemia Target Indicator 2·2·3) are specific, tailored, and measurable. Finally, by averaging across countries rather than populations, the process treats the signatory nations as equal participants, with equal responsibility.

#### Moving beyond prevalence

Anaemia prevalence is a simple, unidimensional indicator of the condition, but it is dominated by mild anaemia. To better capture the multifaceted impact of anaemia on human well-being, we propose that the disease burden of anaemia should be considered rather than the overall prevalence. Burden assigns a defensibly greater weight to severe and moderate cases, due to their greater impacts on health and wellbeing. This approach ensures that reducing the severity of anaemia, not just its prevalence, is adequately prioritised and acknowledged. By emphasising the reduction of more severe cases, burden aligns more closely with health impact and human development goals, providing a clearer picture of progress and guiding targeted interventions that address the most debilitating impacts of anaemia. An indicator based on disease burden such as the disability-adjusted life year (DALY) would be no more complex than overall prevalence (i.e., it is a one-dimensional metric), but it better reflects the impact that the disease has on the population. Finally, this greater accuracy does not impose any additional data collection requirements: it is simply a disability-weighted sum of the severity categories, rather than an unweighted sum.

#### Focus on women of reproductive age

Anaemia can affect individuals of any age or gender. Rather than focusing exclusively on a single subpopulation, future development targets could consider the entire population, including children and men. This broader scope would align with principles of equity and universality central to the SDGs and ensure that the long-term impacts of anaemia in children and other vulnerable groups are prioritised. Moreover, a broader demographic target would not necessarily detract attention from WRA. Since they bear a disproportionate burden of anaemia, any interventions targeting this group would deliver disproportionate reductions in prevalence. Nevertheless, WRA are the subpopulation most affected by anaemia,^35^ are often underserved by public health interventions (appendix p. 36)^315^ and their role as mothers also places them at the core of efforts to reduce childhood anaemia and improve other infant and child health outcomes. Consequently, we believe that WRA should remain the central focus of global development targets for anaemia.

#### Accounting for intervention effectiveness, costs and value for money

How we set rational anaemia reduction targets will depend on several factors: existing levels of coverage for proven anaemia interventions, the effectiveness and costs of increasing this coverage, and the resulting value for money of scaling up interventions in comparison to willingness-to-pay thresholds for improvements in health.

A key consideration in determining targets which are achievable is to identify and scale up interventions that are effective in reducing anaemia burden.^316^ For instance, if a country is already applying all proven anaemia interventions across all WRA, it is hard to justify setting a large prevalence reduction target. Anaemia reduction targets should therefore take into consideration the existing coverage of evidence-based interventions, and the estimated impact on anaemia burden of scaling up available interventions.

Estimating the costs associated with strategies to reduce anaemia is also challenging and will vary substantially between countries. Costs can also be incurred from multiple perspectives, such as regulations on private industry (e.g., through requirements for food fortification). Commitments to targets requires adequate planning and budgeting for nations, which can be impeded by a lack of high-quality estimates of the costs of implementing and scaling up interventions. Since the process of setting global targets does not generate financial resources, countries still need to consider how to fund their commitments, and budget impact and feasibility must also be considered in making decisions on healthcare spending.^317^

Finally, targets for anaemia reduction should take into account country contextualised cost-effectiveness, or value for money, of the interventions under consideration, in comparison to countries willingness-to-pay thresholds for reductions in disease burden. There remains ongoing debate around how country-level cost-effectiveness thresholds should be set, and different estimates have been proposed in the literature.^317^ That said, it is also important to question whether global donors may have different willingness-to-pay thresholds.^318^

#### A proposal for evidence-based target setting

Applying the principles of change outlined above to determine nationally tailored targets that consider burden of disease, intervention effectiveness and coverage, and economic contexts, we propose that targets for anaemia reduction be informed by health economic analysis. Health economic models, tailored to each country, can evaluate what might be accomplished with a cost-effective investment in locally appropriate interventions. This would extend and enhance established approaches to strategic planning for health spending, which use predictive models to estimate changes to disease rates following a change in the coverage of effective interventions and apply these to target-setting (appendix p. 36).^319^

**Figure 8:**
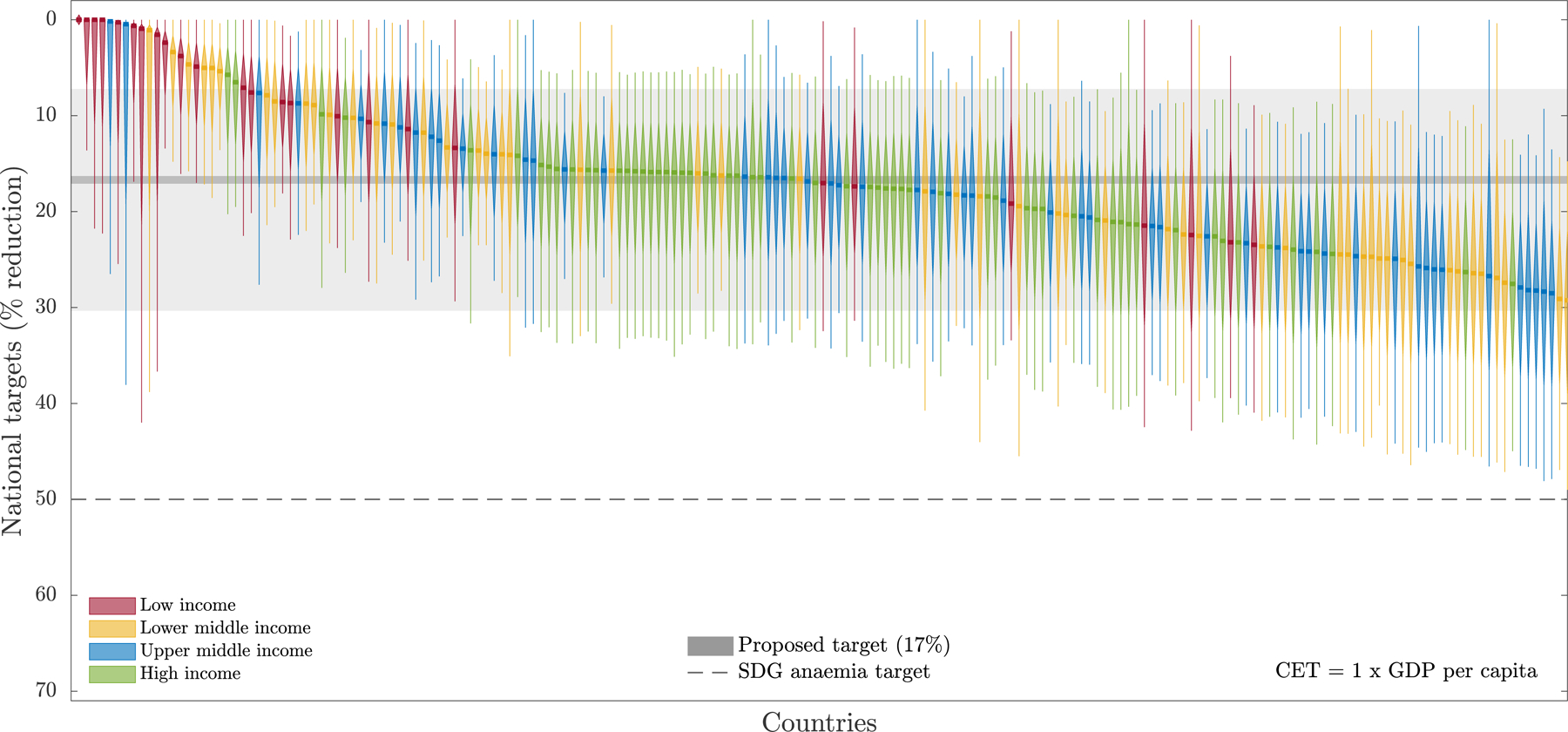

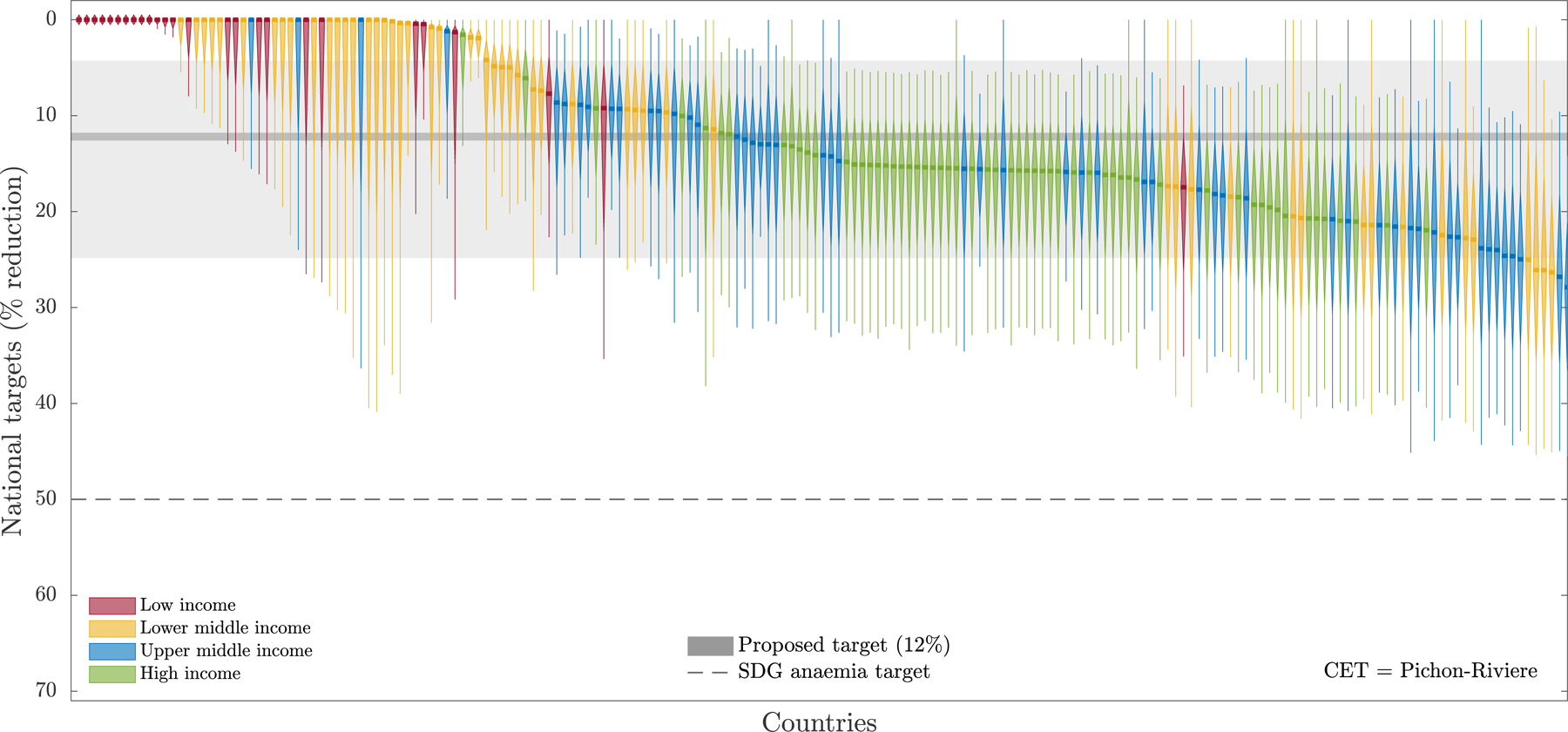

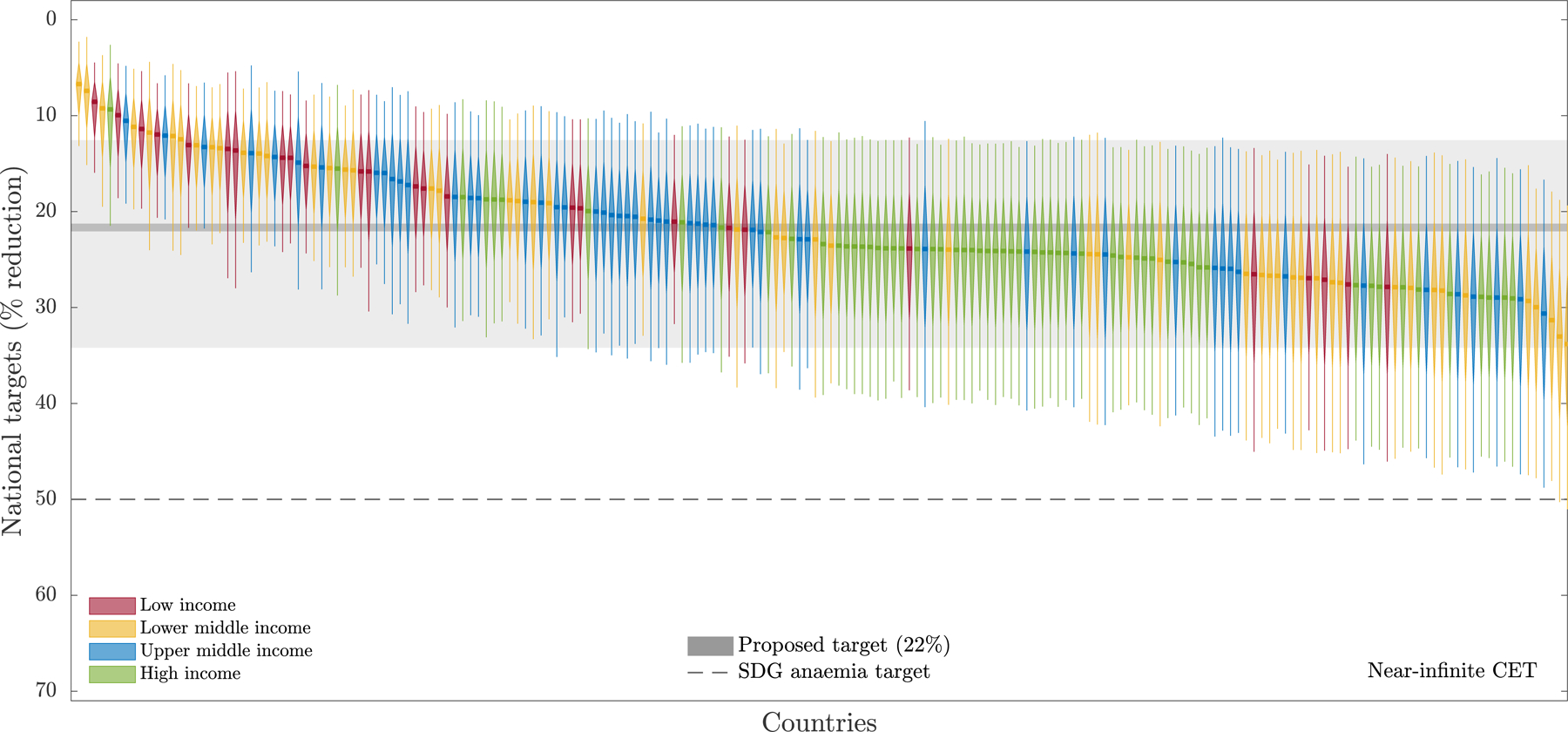
Nationally tailored anaemia reduction targets, and global summary targets, under different potential cost-effectiveness thresholds (CETs). Each panel presents the anaemia reduction targets for 191 countries, colour-coded by income level. Grey line indicates the global summary target; light grey shaded region encloses 95% of global targets across Monte Carlo simulations. Panel A: With a CET of 1 x gross domestic product (GDP)/capita, the global summary target is 17%, with national targets ranging from 0% to ~30%. Panel B: Under a lower CET that approximates an opportunity-cost based approach, the global summary target decreases to 12%, with national targets also ranging from 0% to ~30%. Panel C: When cost constraints are removed, the global summary target rises to 22%, with national targets ranging from ~5% to ~35%. In all scenarios, the global summary targets remain well below the 50% target set by the SDGs and GNTs.

In Box 5 and Table 6, we outline a hypothetical implementation of our proposed target-setting process, which employs a country-level cost-effectiveness analysis to define the optimal set of interventions to put into place. The approach accounts for current anaemia prevalence and severity, the effectiveness of available interventions and their baseline coverage, and the costs of scaling up these interventions. In Figure 8, we report the results of an initial application of these methods to 191 SDG signatory countries. Considering a range of potential national cost-effectiveness thresholds, this analysis calculates that the next global target should aim to reduce average anaemia prevalence in the range of 12–22% over the next international development goal period. However, this single global target summarises a wide variety of national targets, that range from no reduction at all, through to reductions as large as ~35%.

Note that these estimates are limited by the accuracy of our inputs. These were taken from publicly available datasets, and many missing values had to be interpolated from similar countries. Any implementation of this approach would need to ensure that national policymakers have the opportunity to engage directly with the underlying model, with countries encouraged to replace global-scale estimates with locally sourced data – e.g., more precise estimates of intervention costs, coverage, and effectiveness – and to align target-setting with their own budget constraints, cost-effectiveness thresholds and broader health objectives. For example, a country with higher health expenditure or donor support for anaemia programs may set more ambitious targets by adopting a higher cost-effectiveness threshold. Incorporating national expertise and real-world constraints into the target-setting process will help to ensure that international health targets are grounded in rigorous evidence, while being responsive to national needs.

Our new process introduces two key limitations. First, health economic modelling is an uncertain process, particularly when undertaken at a global scale. Setting targets at a national level is complicated by uncertainty around the values of key covariates (e.g., baseline intervention coverage). Cost-effectiveness will be country-specific and determined by local willingness-to-pay thresholds for a given outcome, which can be influenced by subjective or even arbitrary decision-making.^320^ Inaccurate parameter estimates could also lead to strategies being proposed that are less effective, more costly, or inappropriate for the local context. Some of our modelling assumes that particular parameters are uniform across all countries, such as the effect size of each intervention on anaemia prevalence. The substantial uncertainty shown in the Figure 8 – in each of the national reduction targets, as well as in the global summary target – reflects as many of these sources of uncertainty as we could characterise.

Second, our revised process applies an academic approach to setting national reduction targets, with limited participation by the intended end-users. This top-down approach lacks insights into local conditions, preferences, and capacities. It makes it very difficult to incorporate the local knowledge and expertise needed to identify viable and culturally sensitive interventions. An absence of participation in the target-setting process can therefore lead to misalignment with local and national priorities, and a lack of ownership and commitment from the institutions expected to adopt the targets. Moreover, our centralised approach to target-setting stands in direct contrast with the ethos of the SDGs, which emphasise participation, solidarity, and empowerment at both national and global levels.

We propose that an iterative and participatory approach to target setting can simultaneously address the dual limitations of data uncertainty and local participation. In this process, the modelled national targets become the initial conditions of a decision-support system with end-users, particularly national governments, actively participating in the target-setting process. While the model will initially be based on centrally estimated parameters based on available data, national decision-makers will be able to refine these parameters based on local knowledge and priorities. For example, if a particular country has more accurate estimates of baseline coverage for a particular intervention, such as staple food fortification, then they can update the estimates taken from the Global Food Fortification Data Exchange.^321^ Or, if decision-makers feel that our estimate of their cost-effectiveness threshold is too high, then they can override any of our initial parameters.

By providing a transparent platform for collaboration and decision-making, the model thus becomes a dynamic tool for facilitating dialogue between national and international organisations, while retaining a consistent process across all countries. Ideally, the iterative back-and-forth updating will help to ensure that target-setting is not only informed by transparent, rigorous analysis, but will also create a sense of ownership by each country and better reflect the needs of the communities they represent.

#### Challenges related to Future Target Setting

Our recommendations propose two important changes to the international development goals for anaemia. The most obvious change is to the magnitude of the targets themselves, which we propose should change markedly – our results (Box 6; Figure 8) suggest that the current international development targets for anaemia are too large, perhaps by a factor of two. The second change is to the process by which these targets are set. We propose that health economic modelling be used to tailor targets to national circumstances, creating anaemia reduction goals that are achievable as well as ambitious. This combination of modelling and cost-effectiveness principles aligns with common practice in the health economic literature and in philanthropic decision-making. Ideally, each signatory nation will participate in the target-setting process directly, with the models being made available in an interactive format, to invite feedback and input from decision-makers and stakeholders in each country. The wide variety of targets that result from this process will be reconciled under a single, global summary target, averaged across all signatories.

Targets set following this process will be more likely to deliver on both the explicit purposes of international development goals – ambition and achievability – as well as their implicit purposes – a collective vision, equity, and effective communication. However, lengthy negotiations are required to reach consensus on international development goals ^293^, and the changes we propose will prolong this process. The resources and time required to create and iterate national-level health economic models may delay the target-setting process.

From an analytical perspective, our proposed approach is limited by the exclusive use of intervention-focused cost-effectiveness as the basis for determining achievable targets. Public health planning is increasingly moving away from siloed strategies,^322^ towards treating individual diseases as nodes in a complex network of interconnected health issues.^323^ This movement is mirrored by the SDGs, whose holistic goals (e.g., Goal 16: Peace, Justice and Strong Institutions) recognise that development challenges cannot be solved in isolation. By focusing exclusively on the costs of treatment and the burden of disease, our methods may inadvertently overlook opportunities to tackle multiple health challenges simultaneously. Interventions that improve socioeconomic conditions, for example, are closely linked to anaemia prevalence.^324^

This Commission suggests that countries prioritise anaemia interventions based on their comparative cost-effectiveness. However, this does not imply the potential corollaries: that cost-effectiveness analyses are used to determine whether anaemia should be a candidate for public health investment; or, that cost-effectiveness be applied to direct funding between broader development goals. For example, whether funding should be reallocated from anaemia reduction towards breastfeeding (GNT Target 5). At a tactical level, where we compare different anaemia-specific interventions, cost-effectiveness is an appropriate approach. This assumes that decision-makers are agnostic about which techniques are used to achieve their development vision. At a strategic level, by contrast, when comparing qualitatively different objectives such as malnutrition (SDG 2) and clean water (SDG 6), the allocation of resources should reflect national development priorities.

## Conclusions

The year 2030 will conclude the current round of international development goals, including the current anaemia reduction targets. While it is too late to meet our current commitments, we can begin to look beyond the SDGs and GNTs, to identify what changes we must make to get back on track.

Here and in Box 7, we highlight the key recommendations to reduce the global burden of anaemia. To track anaemia reduction progress towards the next targets, accurate, population-based data is fundamental. A strengthened population-based data landscape can facilitate decision-making, particularly the prioritisation of research and interventions, and can offer a more solid foundation for future target-setting. To improve data quality and interpretation, we recommend developing a central data repository for anaemia and its causes and leveraging existing survey platforms that already collect venous blood to add haemoglobin assessment. Additionally, promoting the coordinated use of household surveys together with complementary data sources, and establishing a standardised micronutrient survey to periodically collect data in all affected countries will provide a more holistic understanding of anaemia trends and causes. Ongoing funding and coordination of demographic and health surveys into the future will remain a crucial need to ensure a harmonised global data platform to undertake situational analysis and measure progress towards global targets including anaemia.

Anaemia is a complex public health issue with multi-factorial causes and requires a comprehensive approach. We emphasise the need to close critical gaps in understanding the causes of anaemia and diagnostic challenges to inform the development of targeted intervention strategies. Exploring the underlying mechanisms linking micronutrient deficiencies, malnutrition, infections, socio-economic, and environmental factors, to anaemia across diverse populations in different geographical regions is essential. Additionally, improved identification of heavy menstrual bleeding and affordable point-of-care diagnostics for α-thalassemia and other haemoglobinopathies in prevalent areas are critical.

From a nutritional standpoint, anaemia needs to be addressed comprehensively using evidence-based, context-specific, cost-effective and sustainable interventions. We advocate for innovative nutritional interventions, focusing on promoting equity (particularly gender-equitable access to high-quality food sources so that women and girls can meet their physiologic needs for iron and other micronutrients), sustainability, scalability, and long-term impact. To achieve effective prevention and management of anaemia using nutritional interventions, it is essential to critically evaluate past interventions, recognise their limitations, and identify areas of innovation, including exploring avenues for optimising nutritional responses and transforming social norms. Beyond nutrition, interventions such as infection control, transformative WASH initiatives, and cash transfer programmes should be investigated further to determine their effectiveness in anaemia prevention among vulnerable populations, especially in LMICs. Health systems interventions should focus on increasing women and girls’ access to reproductive health care for family planning, antenatal and postpartum care, and diagnosis and treatment of menstrual disorders that may lead to heavy menstrual bleeding.

Successful implementation of anaemia programmes requires strong governance structures and accountability at global, national, and sub-national levels. We recommend the establishment of clear governance frameworks that promote coordinated action, ensuring accountability and oversight in the fight against anaemia. Expanding on existing national nutrition strategies or creating new anaemia-specific frameworks that integrate cross-sectoral coordination is key for effective anaemia reduction. It is imperative to determine and address implementation barriers to integrating health programmes, including malaria control and nutrition programmes for anaemia reduction. Furthermore, policies and interventions should prioritise social justice and human rights, ensuring that interventions reach the most vulnerable populations. Countries can align their national strategies with targets they participate in setting for the reduction of anaemia.

Failing to achieve self-imposed targets is discouraging, and with the benefit of hindsight, we can see that the ambitious 2030 targets set for anaemia reduction by both the GNTs and the SDGs were never going to be met. This is clear from both the limited reductions in prevalence achieved over the past decade, and also from the health economic modelling predictions we summarise here. This difference between ambition and achievements is not unique to anaemia: previous sets of international development goals were also characterised by a widespread inability to reach their targets, and reviews of progress towards other targets in the SDGs and GNTs demonstrate a similar lack of progress (appendix p. 37).^6^

The next set of post-2030 development goals presents an opportunity to strike a balance between ambition and achievability, leading to potentially greater improvements in human health and well-being. Anaemia is just one of the many targets set in the SDGs but might serve as a template for broader improvement if this approach is effective. A participatory and evidence-based method of target-setting, as we have proposed in this Commission, could lead to more meaningful progress and tangible improvements in global well-being, getting back on track to meet global anaemia reduction targets.

## Supplementary Material

Supplementary Material

## Figures and Tables

**Table 1:** Number of population-based surveys that measured the causes of anaemia among surveys that collected anaemia data between 2000 and 2020, by region.

Cause of anaemia	Global n/N (%)	African n/N (%)	Americas n/N (%)	Eastern Mediterranean n/N (%)	European n/N (%)	South-East Asia n/N (%)	Western Pacific n/N (%)
Chronic diseases	286/407 (70)	90/148 (61)	56/67 (84)	32/47 (68)	38/44 (86)	34/46 (74)	36/55 (65)
Cancer	21/407 (5)	6/148 (4)	6/67 (9)	1/47 (2)	2/44 (5)	5/46 (11)	1/55 (2)
Diabetes	69/407 (17)	13/148 (9)	16/67 (24)	6/47 (13)	12/44 (27)	10/46 (22)	12/55 (22)
Gastrointestinal disease	7/407 (2)	0/148 (0)	2/67 (3)	2/47 (4)	2/44 (5)	0/46 (0)	1/55 (2)
Kidney disease	23/407 (6)	3/148 (2)	5/67 (7)	2/47 (4)	7/44 (16)	3/46 (7)	3/55 (5)
Obesity	282/407 (69)	90/148 (61)	56/67 (84)	30/47 (64)	38/44 (86)	34/46 (74)	34/55 (62)
Infections	NA	NA	NA	NA	NA	NA	NA
HIV	24/119 (20)	24/69 (35)	0/1 (0)	0/9 (0)	0/12 (0)	0/2 (0)	0/26 (0)
Malaria	88/139 (63)	86/134 (64)	NA	1/2 (50)	NA	NA	1/3 (33)
Helminth infection	7/246 (3)	4/127 (3)	0/43 (0)	0/12 (0)	0/9 (0)	1/30 (3)	2/25 (8)
Schistosomiasis	2/210 (1)	2/140 (1)	0/3 (0)	0/29 (0)	NA	0/12 (0)	0/26 (0)
Tuberculosis	0/187 (0)	0/102 (0)	0/12 (0)	0/8 (0)	0/1 (0)	0/29 (0)	0/35 (0)
Visceral leishmaniasis	1/407 (0)	0/148 (0)	0/67 (0)	0/47 (0)	0/44 (0)	1/46 (2)	0/55 (0)
Helicobacter pylori	5/407 (1)	0/148 (0)	3/67 (4)	0/47 (0)	1/44 (2)	1/46 (2)	0/55 (0)
Salmonella	1/407 (0)	0/148 (0)	0/67 (0)	0/47 (0)	1/44 (2)	0/46 (0)	0/55 (0)
Inflammation	67/407 (16)	17/148 (11)	14/67 (21)	11/47 (23)	11/44 (25)	7/46 (15)	7/55 (13)
AGP	45/407 (11)	14/148 (9)	11/67 (16)	8/47 (17)	3/44 (7)	5/46 (11)	4/55 (7)
CRP	59/407 (14)	17/148 (11)	9/67 (13)	8/47 (17)	11/44 (25)	7/46 (15)	7/55 (13)
Micronutrient status	132/407 (32)	27/148 (18)	27/67 (40)	20/47 (43)	21/44 (48)	12/46 (26)	25/55 (45)
Folate	61/407 (15)	11/148 (7)	9/67 (13)	11/47 (23)	16/44 (36)	3/46 (7)	11/55 (20)
Iron	110/407 (27)	20/148 (14)	22/67 (33)	18/47 (38)	21/44 (48)	11/46 (24)	18/55 (33)
Riboflavin	8/407 (2)	0/148 (0)	0/67 (0)	0/47 (0)	8/44 (18)	0/46 (0)	0/55 (0)
Vitamin A	97/407 (24)	26/148 (18)	23/67 (34)	16/47 (34)	8/44 (18)	9/46 (20)	15/55 (27)
Vitamin B12	47/407 (12)	7/148 (5)	13/67 (19)	9/47 (19)	12/44 (27)	2/46 (4)	4/55 (7)
Gynaecological and obstetric conditions	140/407 (34)	72/148 (49)	31/67 (46)	11/47 (23)	11/44 (25)	10/46 (22)	5/55 (9)
Heavy menstrual bleeding	4/407 (1)	0/148 (0)	1/67 (1)	2/47 (4)	0/44 (0)	0/46 (0)	1/55 (2)
Haemorrhage	33/407 (8)	6/148 (4)	14/67 (21)	3/47 (6)	3/44 (7)	4/46 (9)	3/55 (5)
Caesarean section	134/407 (33)	72/148 (49)	29/67 (43)	8/47 (17)	11/44 (25)	10/46 (22)	4/55 (7)
Inherited red blood cell disorders	12/407 (3)	4/148 (3)	0/67 (0)	4/47 (9)	1/44 (2)	1/46 (2)	2/55 (4)

(Denominator determined based on whether the cause is an expected public health problem in a country; Inflammation: AGP = Alpha-1 acid glycoprotein; CRP = C-reactive protein; Obesity: include surveys with anthropometry measurements; Inherited red blood cell disorders: α-thalassemia, β-thalassemia, sickle cell disease, G6PD deficiency, South-East Asian ovalocytosis; Caesarean section: include surveys with elective and emergency caesarean sections; Helminth infection: Roundworm, whipworm, hookworm, strongyloides stercoralis; Micronutrient status: Folate (red blood cell folate, serum folate); Iron (body iron, ferritin, transferrin receptor,); Riboflavin (erythrocyte glutathione reductase activity coefficient; vitamin A (modified relative dose response, retinol, retinol-binding protein); vitamin B12 (serum vitamin B12))

**Table 2:** Potential role of various micronutrient (beyond iron) deficiencies in the development of anaemia.

Micronutrients	Potential impact of micronutrient deficiencies on iron metabolism/anaemia	Populations at risk
**Vitamin A (retinol)** (appendix p.23) ^46^	Impaired mobilisation of iron stores and erythropoiesis; impaired lymphopoiesis increases the risk of infection which can cause anaemia through inflammation and impaired iron metabolism.	Preschool children, pregnant women and women of reproductive age in LMICs.
**Vitamin B2 (riboflavin)** (appendix p.23) ^46^	Impaired mobilisation of iron stores and globin production (leading to impaired erythropoiesis); reduced iron absorption.	Pregnant and lactating women, infants, school-aged children and the elderly.
**Vitamin B6 (pyridoxine)** (appendix p.23) ^46^	Impaired haem synthesis, leading to impaired erythropoiesis; microcytic or normocytic anaemia.	Not well documented.
**Vitamin B9 (folate/ folic acid)** (appendix p.23) ^46^	Impaired DNA synthesis and cell division, leading to impaired erythropoiesis; resulting in macrocytic anaemia.	Pregnant women, preterm infants and individuals living in malaria-endemic regions.
**Vitamin B12 (cobalamin)** (appendix p.23) ^46^	Impaired folate metabolism, DNA synthesis and cell division, leading to impaired erythropoiesis; resulting in macrocytic anaemia.	The elderly, vegetarian and vegan mothers and their infants. Populations with similar diets are also at risk.
**Vitamin C (ascorbic acid)** (appendix p.23) ^46^	Reduced absorption of non-haem iron and mobilization of iron stores; haemolysis due to oxidative damage and blood loss due to capillary haemorrhaging.	Pregnant women, infants fed exclusively with cow’s milk, the elderly and smokers.
**Vitamin D (calciferol)** (appendix p.23) ^326^	Impaired erythropoiesis by reducing erythroid progenitor proliferation, increasing pro-inflammatory cytokines, and elevating hepcidin levels.	Chronic kidney disease, end-stage heart failure, and type 2 diabetes patients, exclusively breastfed infants, people with limited sun exposure, and individuals with darker skin tones.
**Vitamin E (tocopherol)** (appendix p.23) ^46^	Haemolysis due to oxidative damage.	Premature and low-birth-weight infants, and individuals with pathological malabsorption syndromes.
**Copper** (appendix p.23) ^46^	Impaired iron metabolism and erythropoiesis (which requires copper-dependent enzymes).	Premature and low-birth-weight infants fed milk diets, infants and children recovering from malnutrition or prolonged diarrhoea, individuals with malabsorption e.g. with celiac or Crohn’s disease, people with Menkes disease.
**Calcium** (appendix p.23) ^327^	Calcium is involved in proliferation and differentiation of erythroid progenitor cells, terminal enucleation, and mature red blood cell aging and clearance. The presence of calcium in diets may affect iron absorption by competing for shared transport pathways.	Pregnant and lactating women, postmenopausal women, the elderly, individuals with chronic diseases, including chronic kidney disease, and lactose intolerant individuals.
**Zinc** (appendix p.23) ^328^	Inhibits iron absorption. Impaired protein function (haem biosynthetic enzyme, erythropoietin, hepcidin).	Pregnant women, infants and children, individuals consuming vegetarian and vegan diets, individuals with gastrointestinal conditions like Crohn’s disease, ulcerative colitis, or chronic diarrhoea, and the elderly.

**Table 3:** Haemoglobinopathies contributing to the global burden of anaemia

Haemoglobinopathy type	Genetic basis	Clinical phenotype	High prevalence regions
***α-Thalassaemia***			Mediterranean, Middle East, South, South-East and East Asia, and Africa
Silent α-thalassaemia	1 (out of 4) α-globin gene deletion [α−/αα]	Asymptomatic	
α-thalassaemia trait	2 (out of 4) α-globin gene deletion [−α/−α,or − −/αα]	Asymptomatic anaemia, mild microcytosis	
HbH disease	3 (out of 4) α-globin gene deletion [− −/− α]	Non-transfusion dependent thalassaemia	
Hb Barts hydrops fetalis	4 α-globin gene deletion [− −/− −]	Foetal anaemia and hydrops, usually fatal without intrauterine transfusion	
***β-Thalassaemia***			Mediterranean, Middle East, South, South-East and East Asia, and Africa
β-thalassaemia trait	Heterozygous β-thalassaemia mutation (β/β^0^, β/β^+^, β/β^++^)	Asymptomatic anaemia, microcytosis	
β-thalassaemia intermedia	Homozygous or compound heterozygous β-thalassaemia mutation (β^++^/β^++^)	Non-transfusion dependent thalassaemia	
β-thalassaemia major	Homozygous or compound heterozygous β-thalassaemia mutation (β^0^/β^0^, β^+^/β^+^, β^0^/β^+^)	Transfusion dependent thalassaemia	
***Haemoglobin E***			South and South-East Asia
HbE trait	Heterozygous β^E^-globin mutation (β/β^E^)	Asymptomatic	
HbE disease	Homozygous β^E^-globin mutation (β^E^ /β^E^)	Mild anaemia, clinically asymptomatic	
HbE thalassaemia	Compound heterozygous for β^E^ and β-thalassaemia (β^0^/β^E^)	Non-transfusion-dependent or transfusion-dependent thalassaemia	
***Haemoglobin C***			West Africa and South-East Asia
HbC trait	Heterozygous β^C^-globin mutation (β/β^C^)	Asymptomatic	
HbC disease	Homozygous β^C^-globin mutation (β^C^/β^C^)	Mild-moderate anaemia	
***Haemoglobin S***			Africa, Middle East, and South Asia
Sickle cell trait	Heterozygous β^S^-globin mutation (β/β^S^)	Asymptomatic, no anaemia	
Sickle cell anaemia	Homozygous β^s^-globin mutation (β^S^/β^S^).	Mild-moderate anaemia and vaso-occlusive crisis	
Sickle thalassaemia	Compound heterozygous for β^S^ and β-thalassaemia (β^0^/β^S^)	Mild-moderate anaemia and vaso-occlusive crisis	
Haemoglobin SC disease	Compound heterozygous for β^S^ and β^C^ (β^S^/β^C^)	Mild anaemia and vaso-occlusive crisis	

**Table 4:** Knowledge gaps and recommendations in anaemia aetiology and management

Knowledge gaps in anaemia aetiology and management	Recommendations
1) Gaps in mechanistic understanding of anaemia drivers: a. Lack of understanding of how factors like micronutrient deficiencies, malnutrition, infections, heavy menstrual blood loss, environmental enteropathy, and upstream environmental factors interact to contribute to anaemia across populations. b. Limited knowledge on how these interactions vary across life stages or during infection. c. Need to determine how dietary patterns affect iron status and anaemia risk, including optimal algorithms to measure dietary iron bioavailability.	1) Closing knowledge gaps in anaemia drivers: Conduct targeted research to understand key causes and underlying mechanisms of anaemia, including the interaction of environmental, nutritional and physiological factors.
2) Challenges in the assessment of anaemia determinants: a. Lack of improved or standardised tools for detecting heavy menstrual bleeding and haemoglobinopathies. b. Lack of clear criteria for distinguishing IDA, anaemia of inflammation, and unexplained anaemia, particularly in populations with infections, chronic conditions, or in the elderly. c. Challenges in accurately measuring micronutrient deficiencies due to lack of standard biomarkers, cut-offs, and the high cost of assays. d. Complexities in assessing environmental determinants like air pollution and climate change as they require sophisticated models and long-term data collection to account for regional variations.	2) Addressing challenges in anaemia determinants assessment: a. Develop and implement improved or standardised tools for measuring key determinants such as heavy menstrual bleeding, haemoglobinopathies, and other contributors to anaemia. b. Advance research to establish cost-effective and standardised assays for micronutrient deficiencies, as well as tools for assessing the impact of air pollution and climate change on anaemia.
3) Knowledge gaps in nutrition interventions: a. Uncertainty regarding the most effective dosing, formulations, and safety of iron therapy across different populations. b. Unclear optimal combination of micronutrients to address anaemia. c. Insufficient approaches to optimise fortification and biofortification strategies that optimise nutrient bioavailability in staple foods for vulnerable groups. d. Gaps in the understanding of the role of exclusive breastfeeding and the timing of complementary feeding on infant iron status and anaemia.	3) Improving nutritional interventions: a. Investigate optimal iron therapy dosing, formulations, and safety across diverse populations, including those with infections or haemoglobinopathies. b. Identify effective micronutrient combinations tailored to population needs. c. Advance strategies for fortification and biofortification to maximise nutrient bioavailability in staple foods. d. Evaluate the impact of early iron supplementation and improve complementary feeding strategies in LMICs to enhance infant iron status and reduce anaemia.
4) Knowledge gaps in non-nutrition interventions: a. Limited evidence on the effectiveness of postpartum haemorrhage treatments (e.g., TXA, carbetocin), the use of therapies for heavy menstrual bleeding, and the role of birth spacing, in reducing anaemia in LMICs. b. Gaps in integrating infection control with nutrition interventions, and limited evidence on the effectiveness of WASH programmes in anaemia reduction. c. Limited adoption of WHO-recommended delayed cord clamping in high anaemia burden settings in LMICs. d. Limited evidence on the effectiveness of cash transfer and other social protection interventions to reduce anaemia in vulnerable populations.	4) Improving non-nutritional interventions: a. Expand research on the effectiveness and accessibility of postpartum haemorrhage treatments like TXA and carbetocin, especially in LMICs. Investigate therapies for managing heavy menstrual bleeding and strategies for increasing birth spacing. b. Study barriers and facilitators for integrating infection control, nutrition, and WASH programmes to reduce anaemia. c. Conduct implementation research to improve the adoption of the WHO-recommended DCC in LMICs. d. Evaluate the impact of social protection interventions such as cash transfer programmes in addressing anaemia in vulnerable populations.

**Table 5:** Factors shaping effective anaemia implementation, challenges and opportunities at all levels, and recommendations for effective anaemia reduction programme implementation

Principle	Factors shaping effective implementation	Challenges for anaemia targets	Opportunities for anaemia targets	Recommendations for effective implementation of anaemia reduction programmes
Understanding context	1) Aetiology 2) Measurement and Indicators	1) Aetiology is broad, differing by life-stage, gender and geography, and some causes are difficult to address, making it a complex issue to tackle. 2) Adequate precision and accuracy on haemoglobin measurement is unclear 3) Perceived impact of anaemia on morbidity and mortality are not always visible or immediate	1) Spans high- and low- and middle-income countries, so there is global relevance 2) New global guidance	1) Establish clear governance structures and accountability mechanisms at global, national and sub-national levels. 2) Expand national nutrition strategies that integrate anaemia coordination and effective management across multiple sectors. 3) Centre social justice and human rights within anaemia policies and prioritised interventions.
Multi-sectoral governance	1) Policy cohesion 2) Leadership 3) Civil society mobilisation	1) Lack of accountability due to diverse actors and diffusion of power 2) Anaemia policy has been driven by the perspectives of a few individuals	Multidisciplinary, multisectoral solutions for anaemia can offer cost savings in implementing interventions	
Integration	1) Strategic vision 2) Evidence-based interventions 3) Monitoring and evaluation	1) Complexity and cost of precise and accurate measurements and concern about their validity result in lack of commitment 2) Low adherence to interventions due to adverse effects 3) Potential harms associated with population and individually targeted iron interventions in malaria-endemic areas 4) Risk for toxicity with multiple overlapping iron programmes (e.g., fortification, supplementation)		
Equity	Financing	1) Largely a women’s health issue, potentially impairing investment (appendix p. 34)^270^ 2) Solutions have insufficiently addressed underlying social determinants of health	The problem of anaemia has elevated adolescent health on the global agenda	

**Table 6:** Input data and predicted anaemia reduction targets for three hypothetical countries. This table presents key variables used in the model, including baseline anaemia prevalence (mild, moderate, and severe cases), estimated per-person costs (USD) for four interventions (iron supplementation for pregnant women, iron supplementation for women of reproductive age (WRA), IPTp-SP, and staple food fortification), current and maximum feasible intervention coverage, and predicted anaemia reduction targets. Targets are calculated under two cost-effectiveness thresholds (CETs): an opportunity cost CET taken from Pichon-Riviere’s study ^329^ which reflects budget constraints and competing health priorities, and a ‘near infinite’ CET scenario, where interventions are scaled up to their maximum feasible coverage without cost considerations.

	Current Anaemia Prevalence	Unit costs of each intervention (per woman)	Current level of intervention coverage	Maximum feasible level of coverage	Anaemia reduction target w/ uncertainty (near infinite CET)	Anaemia reduction target and uncertainty bounds (CET: Pichon-Riviere)
Low-income Country X	Mild: 19% Mod: 15% Severe: 1% Overall: 35%	Iron (antenatal): $4.12 Iron (WRA): $3.70 Antimalarials: $2.62 Fortification: $0.73	Iron (antenatal): 85% Iron (WRA): 35% Antimalarials: 7% Fortification: 1%	Iron (antenatal): 91% Iron (WRA): 91% Antimalarials: 91% Fortification: 37%	Median: 16% Lower: 9% Upper: 26%	Median: 1% Lower: 0% Upper: 18%
Middle-income Country Y	Mild: 12% Mod: 5% Severe: 0% Overall: 18%	Iron (antenatal): $41.14 Iron (WRA): $23.45 Antimalarials: $0.85 Fortification: $0.69	Iron (antenatal): 81% Iron (WRA): 33% Antimalarials: 0% Fortification: 30%	Iron (antenatal): 100% Iron (WRA): 100% Antimalarials: 100% Fortification: 100%	Median: 26% Lower: 14% Upper: 42%	Median: 21% Lower: 8% Upper: 39%
Low-income Country Z	Mild: 26% Mod: 17% Severe: 1% Overall: 44%	Iron (antenatal): $4.21 Iron (WRA): $4.12 Antimalarials: $2.72 Fortification: $0.04	Iron (antenatal): 36% Iron (WRA): 15% Antimalarials: 0% Fortification: 10%	Iron (antenatal): 72% Iron (WRA): 72% Antimalarials: 72% Fortification: 80%	Median: 27% Lower: 16% Upper: 43%	Median: 18% Lower: 7% Upper: 36%

## Box 2: Impact of 2024 WHO guidelines on haemoglobin cutoffs and altitude adjustments to define anaemia.

**Table:**

To assess the impact of the revised haemoglobin guidelines for diagnosing anaemia, we selected five countries across the Africa, Americas, and South-East Asia regions that were representative of a range of populations residing at different altitudes (see Figure 4, Appendix Tables 6–8 on p. 15–18 and Appendix Figure 3 on p. 18). Among children 6–59 months, updates to the altitude adjustment guidelines resulted in a more pronounced increase in estimated anaemia prevalence compared with the decrease in anaemia estimates caused by lowering the haemoglobin cutoff in the subset of children that are 6–23 months. We found the median change in anaemia prevalence was 4.5 percentage points (range: −3.9 to 12.2) when both the new cutoff and altitude adjustments were applied, 9.2 percentage points (range: 0 to 16) when only new altitude adjustments were applied, and −4.7 percentage points (range: −5.5 to −3.3) when only new cutoffs were applied. Among women 15–49 years, the increase in anaemia estimates when applying the new compared with the old altitude adjustment resulted in a median increase of 6 percentage points (range: 0 to 13.5). Smoking adjustments had minimal impact due to low smoking prevalence in these populations (data not shown). These estimated effects of the revised WHO guidelines for defining anaemia align with recent findings from the BRINDA research group, which reported a net increase in anaemia of 4.7 percentage points in children (accounting for new cutoffs and altitude) and 15.3 percentage points in women (accounting for altitude and smoking).^330^ The direction and magnitude of changes in anaemia prevalence may vary depending on the elevation and haemoglobin distribution of the population being assessed, particularly in children as cutoffs also differ by age.

## Box 3: Challenges in anaemia surveillance and approaches to strengthen the anaemia data landscape

**Table:**

Challenges in anaemia surveillance	Approaches to strengthen the anaemia data landscape
○ Limited data, particularly: ● Population-level anaemia data in most countries-especially in the European region ● Population-level anaemia prevalence estimates for children below six months, school-aged children, adolescents and older adults ● Data on the main causes of anaemia from population-representative surveys measured concurrently with Hb levels, or measured separately and linked ○ Challenges in Hb/ anaemia assessment: ● Insufficient evidence on acceptable uncertainty for haemoglobin assessment at population-level surveys. ● Need for development of the application of automated haematology analysers for use in low-income settings suitable for population-survey use. ● Challenges in statistical analysis approaches to disentangle the different causes of anaemia that could better understand the drivers of anaemia in different contexts	♦ Develop a standard data repository on anaemia and its causes that includes microdata (dataset that contains the raw data and derived variables), as well as paradata (supplementary data on the data collection process that is a byproduct of the data being collected) and metadata (information about the design and the data collected) that can provide insight into the quality of data and support interpretation. ♦ Establish a standardised micronutrient survey, either through The DHS Programme or a newly established platform, to periodically collect comprehensive and standardized data in all affected countries. ♦ Integrate use of data between household surveys and other data sources, such as facility-based surveys, surveillance systems, registries, case notifications, and routine health data. ♦ Leverage suitable population survey platforms that already collect venous blood to add haemoglobin assessment, e.g. Population-based HIV Impact Assessment surveys.^331^

## Box 4: Case studies of countries with successful implementation that led to anaemia reduction.

**Table:**

Since the start of the millennium, several countries have substantially reduced the burden of anaemia among vulnerable population groups. Taking a positive deviance approach, understanding which factors contribute to success in reducing anaemia provides valuable insights that enable lessons to be applied to current and future challenges. Note that A positive deviance approach assumes that solutions to development problems already exist within communities. By identifying outlying countries, those which have succeeded in reducing specific health and nutrition outcomes beyond GDP, allows one to examine the implemented policies and interventions, which can be applied to other country contexts. For example, in the Philippines,^332^ an 11% decline in anaemia prevalence in women of reproductive age was reported between 2008 and 2018^332^. Key direct and indirect nutrition programmes were introduced during this time including universal healthcare and food fortification. Country experts have also credited programmes focused on alleviating micronutrient deficiencies and poverty, and improvements in women’s health and well-being, as contributing factors to the country’s reduction in anaemia prevalence. India’s Anaemia Mukt Bharat programme was launched in 2018,^333^ which focused on reducing anaemia from 50% in 2016 to 32% by 2022.^333^ The programme followed a 6 × 6 × 6 strategy, which aims to reduce anaemia among six beneficiaries age groups through implementation of six interventions and six institutional mechanisms, estimated to reach 450 million beneficiaries. National and subnational governments adopted a holistic approach to address systemic concerns through improved intra-ministerial coordination, convergence with other ministries, strengthening of supply chain and logistics and developing a dashboard which integrated key indicators from the Health Management Information System. These case studies reinforce the importance of adopting comprehensive, multifaceted strategies to combat anaemia effectively. They not only highlight the significance of addressing both direct and underlying determinants of anaemia but also serve as a guide for other nations striving to achieve similar outcomes. As we look to the future, leveraging these insights will be crucial in shaping effective interventions and ensuring sustained progress.

## Box 5: Identifying ambitious and achievable anaemia reduction targets for each nation

**Table:**

We developed a generalisable health economic model to set tailored, achievable, and ambitious anaemia reduction targets for each country. To choose targets, the model integrates information on anaemia prevalence and severity, the effectiveness and current coverage of different interventions, unit costs of delivery, and potential country-specific cost-effectiveness threshold (CET) in terms of cost per disability-adjusted life year (DALY) averted. Full details of the model and results can be found in Blythe et al. (Submitted).^334^ Briefly, data from the Global Burden of Disease study provide baseline estimates of the current prevalence of anaemia among women of reproductive age. We identify WHO-recommended interventions with robust evidence for reducing anaemia prevalence and adjust the reported effectiveness of each intervention to account for implementation factors and national covariates (e.g., malaria endemicity). Country-specific costs for each intervention are estimated using micro-costing. For each of 191 countries, the model searches for the intervention with the most favourable incremental cost-effectiveness ratio (ICER), measured by the reduction in burden per dollar invested. If the ICER falls below the country’s CET, indicating it would be cost-effective, the model then increases the coverage of this intervention to a maximum feasible coverage. Note that in some circumstances, no intervention might be cost-effective. Following this coverage increase, ICERs for all remaining interventions are recalculated, accounting for the modelled change in anaemia prevalence. The process is then repeated for the intervention with the next most favourable ICER until all remaining interventions exceed the country’s cost-effectiveness threshold (CET), or until all interventions are implemented. At this point, the model predicts the prevalence of anaemia in the country and uses the difference between this value and the baseline to create a tailored anaemia reduction target for each nation. The model allows for a range of potential CETs to be considered, including a ‘near infinite’ CET, reflecting implementing all strategies to their maximum feasible coverage with no cost considerations. Uncertainty in each of the input parameters (e.g., burden, intervention effectiveness, coverage, costs, etc.) is propagated through the model using Monte Carlo sampling to generate uncertainty bounds around the proposed target.

## Box 6: Towards setting international targets for anaemia reduction

**Table:**

We undertook a preliminary implementation of the methods outlined in Box 5 and Table 6 to set a new anaemia reduction target for 191 SDG signatory countries. The resulting recommended national anaemia reduction targets display substantial variation, ranging from effectively zero in some countries, up to 30% in others, with considerable uncertainty at the country level. Averaged across countries, the global summary target falls below 20% under two potential cost-effectiveness thresholds CETs (Figure 8, panels A & B). Note that these are not conclusive recommendations: as we outline in the main text, targets should only be set after nations have the opportunity to participate in refining model inputs and assumptions. The variation in national targets highlight the multifaceted and context-specific nature of anaemia, and national differences in underlying burden of disease, important covariates (e.g., malaria prevalence), costs, existing and maximum potential coverage, and health system capacity. They reinforce the importance of tailoring anaemia reduction strategies to each country’s unique demographic and economic context. The considerable uncertainty in our estimates reflects the challenges of setting rational targets for international development, including the need for more accurate information on anaemia prevalence, coverage, and the effectiveness of public health interventions. This underscores the importance of expanding the approach described here to incorporate the knowledge and priorities of country-level stakeholders. Low-income countries often have modest targets (Figure 8, panels A & B), due in part to their more-limited financial capacity to pay, reflected in lower CETs. This means many of the available interventions are not considered cost-effective. By contrast, our model sets higher reduction targets for upper middle-income countries, reflecting their combination of moderate CETs and low levels of existing intervention coverage. In high income countries, our interventions are all considered cost-effective (besides preventative antimalarials, given the low prevalence/absence of malaria). However, targets for high income countries are not commensurately large because of high baseline coverage of proven interventions that limits further opportunities for improvement. Despite these trends, variation in targets within income levels is often larger than the average differences between them. The global anaemia reduction target for WRA is sensitive to CETs used. International development goals, including the SDGs, are intended to attract additional resources to lower-income countries, which could translate into higher thresholds than our estimates. If we remove any cost considerations, allowing countries to fund any proven intervention to the maximum feasible coverage levels, the global target increases to 25% (Figure 8, panel C). Notably, targets for low-income countries increase markedly, and there are no countries with zero targets. Nevertheless, the global target remains much lower than the current SDG and GNT reduction target of 50%, indicating that this level of improvement exceeds what is possible through scaling up available proven interventions.

## Box 7: Summarised list of top 10 recommendations.

**Table:**

**1. Enhance routine data collection:** Broaden population coverage of anaemia epidemiology to include infants, school-aged children, adolescents and the elderly. Improve haemoglobin measurements using venous blood and integrate nutritional and non-nutritional biomarkers into survey platforms. Develop publicly accessible data repositories to support informed decision-making. **2. Establish evidence-based haemoglobin thresholds:** Generate evidence to validate haemoglobin thresholds for anaemia severity classification, aligning them with functional outcomes and health consequences to improve diagnosis and treatment strategies. **3. Close knowledge gaps in anaemia biology:** Conduct targeted research to better understand the biological mechanisms, underlying causes, and context-specific drivers of anaemia. Evaluate the contribution of genetic conditions to anaemia burdens across populations and their interactions with anaemia control programs and reduction targets. **4. Improve and integrate interventions:** Test and scale innovative nutritional solutions for anaemia control, such as novel oral iron formulations, optimised dosing, intravenous iron, and biofortification of staple foods. Integrate these with non-nutritional interventions, including WASH programs, infection control, birth spacing, delayed cord-clamping, and management of heavy menstrual bleeding. Develop context-specific strategies that integrate nutrition-specific and non-nutritional approaches to maximise impact on anaemia reduction. **5. Strengthen governance and coordination:** Develop robust governance frameworks and national anaemia strategies to ensure coordinated actions and effective intervention delivery. Foster intersectoral collaboration for sustainable outcomes. **6. Improve monitoring and accountability systems:** Strengthen monitoring systems to track the implementation, progress, and impact of anaemia interventions at national, sub-national, and global levels. Develop transparent accountability mechanisms and regular reporting frameworks. **7. Prioritise equity and social justice:** Ensure equitable access to anaemia interventions, focusing on vulnerable and marginalised populations. Address gender disparities and adopt a rights-based approach to target those most at risk. **8. Educate and engage stakeholders:** Increase awareness among governments, healthcare providers, public health professionals, donors, and the public about anaemia prevention, management, and its broader health impacts. Leverage advocacy and education initiatives to build momentum for anaemia reduction efforts. **9. Redefine and adopt collaborative anaemia targets:** Develop anaemia reduction targets that are ambitious but realistic, tailored to local contexts, and informed by evidence. Recognise previous successes while identifying opportunities for further progress to create actionable pathways. **10. Increase investment in anaemia research:** Mobilise funding for research focused on basic science, implementation strategies, and innovative solutions to reduce anaemia. Prioritise cost-effective approaches to accelerate progress and improve health outcomes globally.
