# Supplementary material for: Getting back on track to meet global anaemia reduction targets: a Lancet Haematology Commission
Source: Lancet Haematol. Author manuscript; Available in PMC 2026 Jan 6. (PMC12774439; doi:10.1016/S2352-3026(25)00146-2)
Supplement: Supplementary Material [file NIHMS2124103-supplement-Supplementary_Material.pdf]

THE LANCET
Haematology

Supplementary appendix 5

This appendix formed part of the original submission and has been peer reviewed. We post it as supplied by the authors.

Supplement to: Atkinson SH, Suchdev PS, Bode M, et al. Getting back on track to meet global anaemia reduction targets: a *Lancet* Haematology Commission. *Lancet Haematol* 2025; published online Aug 26. https://doi.org/10.1016/S2352-3026(25)00146-2.

Lancet Haematology Commission: Getting back on track to meet global anaemia reduction targets.

Appendix Material

Table of Contents

SECTION 1: THE GLOBAL BURDEN OF ANAEMIA: UNCOVERING DATA GAPS AND CHALLENGES 4

ANAEMIA PREVALENCE ESTIMATES BY POPULATION AND GEOGRAPHY 4

Figure 1: Prevalence of any anaemia in 2021 and data gaps in infants; school-aged children, adolescents according to GBD. 4

Figure 2: Prevalence of any anaemia in 2021 and data gaps in men and older persons according to GBD. 5

Table 1: Commonalities and divergences in methodology for estimating anaemia prevalence between Global Burden of Disease (GBD) and the World Health Organization (WHO). 6

MAJOR DATA GAPS IN ANAEMIA, AND ITS CAUSES IN POPULATION-BASED SURVEYS. 7

Micronutrient databases merge 7

Table 2: Micronutrient and indicator databases. 8

Table 3: Search terms for non-nutritional anaemia causes. 9

Table 4: Number of population-based surveys that measured the number of causes# among surveys that collected anaemia data between 2000 and 2020, by region. 11

ANALYTICAL METHOD CONSIDERATIONS FOR HAEMOGLOBIN MEASUREMENT. 12

Box 1: An example of measuring haemoglobin using venous blood on an automated haematology analyser accompanied by high-quality control measures. 12

ESTABLISHING NEW OR STRENGTHENING EXISTING SURVEY PLATFORMS 13

Table 5*:* Comparison of Survey Platforms for collecting data on anaemia and its causes. 13

RECENT CHANGES IN THE DEFINITION OF ANAEMIA ARE EXPECTED TO AFFECT ESTIMATES OF ANAEMIA. 15

Table 6: Comparison of anaemia prevalence (%) between the new and old cutoffs and altitude adjustment among children aged 6-59 months; and between the old and new altitude and smoking adjustment among non-pregnant women aged 15-49 years, five surveys in African region.# 15

Table 7: Comparison of anaemia prevalence (%) between the new and old cutoffs and altitude adjustment among children aged 6-59 months and between the old and new altitude and smoking adjustment among non-pregnant women aged 15-49 years, five surveys in the region of the Americas.# 16

Table 8: Comparison of anaemia prevalence (%) between the new and old cutoffs and altitude adjustment among children aged 6-59 months and between the old and new altitude and smoking adjustment among non-pregnant women aged 15-49 years, three surveys in the Southeast Asian region.# 17

Figure 3: A) Comparison of anaemia prevalence (%) between the new and old cutoffs and altitude adjustment among children aged 6-59 months in the American Region and in the Southeast Asian Region. B) Comparison of anaemia prevalence (%) between the old and new altitude and smoking adjustment among non-pregnant women aged 15-49 years in the American Region and in the Southeast Asian Region. Error bars denote Wald confidence limits of the percentage. 18

SECTION 3: IMPROVING ANAEMIA CONTROL PROGRAMS: IMPLEMENTATION AND GOVERNANCE 19

CURRENT STATE OF ANAEMIA GOVERNANCE AND IMPLEMENTATION 19

Table 9: Anaemia-related Sustainable Development Goals (SDGs) (2015-2030) 19

ADDITIONAL REFERENCES FOR THE COMMISSION **20**
Box 1 20
SECTION 1: THE GLOBAL BURDEN OF ANAEMIA: UNCOVERING DATA GAPS AND CHALLENGES. **20**
Effects of revised anaemia definitions on prevalence estimates 20
Enhancing data quality: validity and reliability of anaemia estimates 20
Analytical method considerations for haemoglobin measurement 21
Establishing new or strengthening existing survey platforms 21
Using data on anaemia causes to develop context-specific solutions 21
SECTION 2 – ANAEMIA CONTROL MUST CONSIDER ITS COMPLEX MULTIFACTORIAL CAUSES **22**
Physiological iron needs by life stages 22
Role of micronutrient deficiencies in the development of anaemia 22
Table 2: 23
Dietary determinants of iron deficiency and anaemia 23
Dietary iron adequacy and bioavailability *23*
Dietary patterns and iron deficiency and anaemia *24*
Co-occurrence of anaemia and malnutrition 24
Nutritional interventions addressing anaemia: 24
Oral iron supplementation *24*
Multinutrient supplementation to tackle micronutrient deficiencies known to cause anaemia *25*
Industrial food fortification *25*
Food preparation techniques to improve nutrient intake *25*
Agriculture-nutrition programmes *25*
Nutritional education and awareness *26*
Other interventions addressing anaemia: 26
Optimising iron stores through delayed cord clamping *26*
Cash transfer programmes to improve nutrition and address anaemia *27*
Inflammation, infections and anaemia 27
Anaemia of inflammation: the role of hepcidin *27*
Anaemia and Malaria *28*
Tuberculosis disease *29*
HIV *30*
Anaemia due to soil-transmitted helminths and schistosomiasis *30*
The role of gut health in anaemia 31
Anaemia in older people 31
Environmental considerations for anaemia 32
Air pollution *32*
Climate change *32*
Blood loss and anaemia in women of reproductive age 32
Heavy menstrual bleeding *32*
Interventions to reduce heavy menstrual blood loss *33*
Postpartum haemorrhage *33*

SECTION 3: IMPROVING ANAEMIA CONTROL PROGRAMS: IMPLEMENTATION AND GOVERNANCE 34

Current state of anaemia governance and implementation 34

Core Principle 1: leverage existing data and collecting new data 34

Core Principle 2: catalyse multisectoral governance and engagement 34

Global governance for anaemia 34

Current global governance architecture (and Table 5) 34

National government-led anaemia strategies 35

Building and sustaining political commitment for anaemia reduction 35

Mobilising domestic resources and financial flows 35

SECTION 4: A BETTER WAY FORWARD: REDEFINING FUTURE ANAEMIA REDUCTION TARGETS 36

International development goals 36

The purpose of international target-setting 36

Global anaemia targets 36

Focus on women of reproductive age 36

A proposal for evidence-based target setting 36

CHALLENGES RELATED TO FUTURE TARGET SETTING 37

CONCLUSIONS 37

REFERENCES FOR APPENDIX: 38

Section 1: The global burden of anaemia: uncovering data gaps and challenges

Anaemia prevalence estimates by population and geography

Figure 1: Prevalence of any anaemia in 2021 and data gaps in infants; school-aged children, adolescents according to GBD.

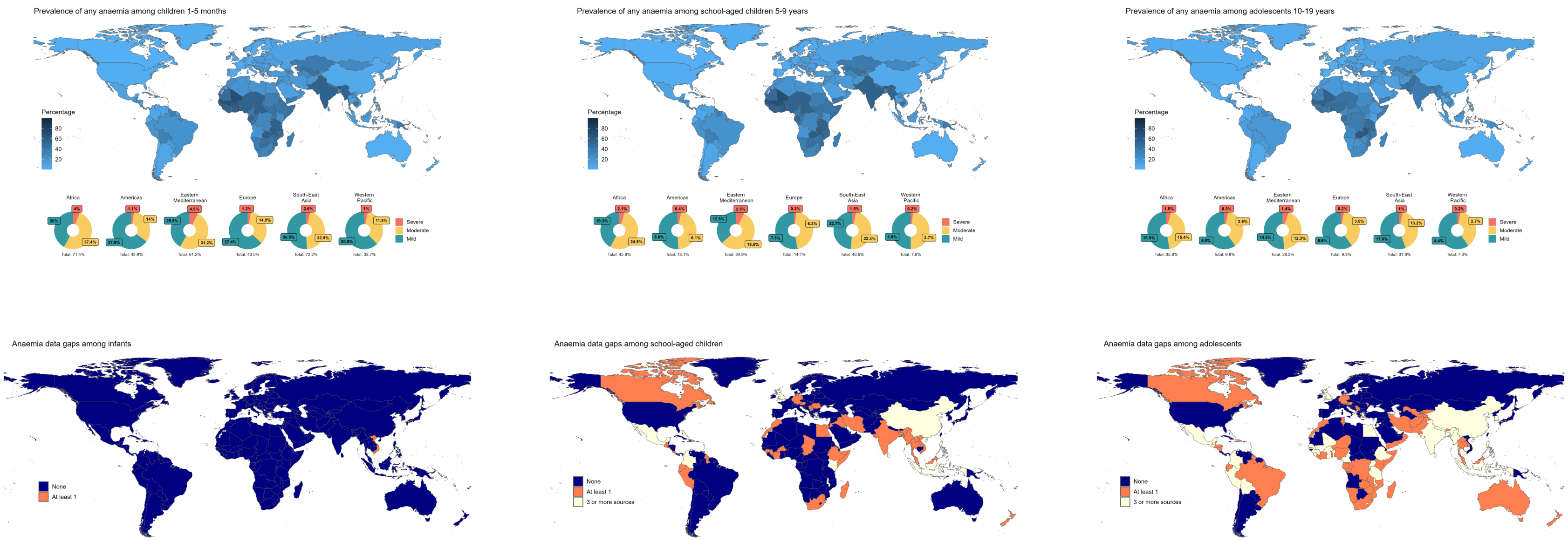

Key (for prevalence): 60, 40, 20.

Key (for gaps): No anaemia data; At least one source (1-2 sources); Three or more sources.

Footnote: Haemoglobin, Hb. Any anaemia defined as Hb <110 g/L in infants 1-5 months; Hb <115 g/L in children aged 5-9 years and in adolescents aged 10-14 years; Data estimates derived from GBD 2023. Estimates are not yet available based on the newly recommended WHO cutoffs and adjustments.[1]

Footnote (for gaps): Data from the Vitamin and Mineral Nutrition Information System (VMNIS) between 2000-2020. Age intervals defined based on WHO's VMNIS classification of population groups and do not exactly cover the age ranges of interest: infants 0-5 months, school-age children 4-19 years, and adolescents 10-49 years. The most common age grouping for each population is 0-5 months for infants (100%), 6-12 years for school-aged children (14%), and 15-19 years for adolescents (46%).

Figure 2: Prevalence of any anaemia in 2021 and data gaps in men and older persons according to GBD.

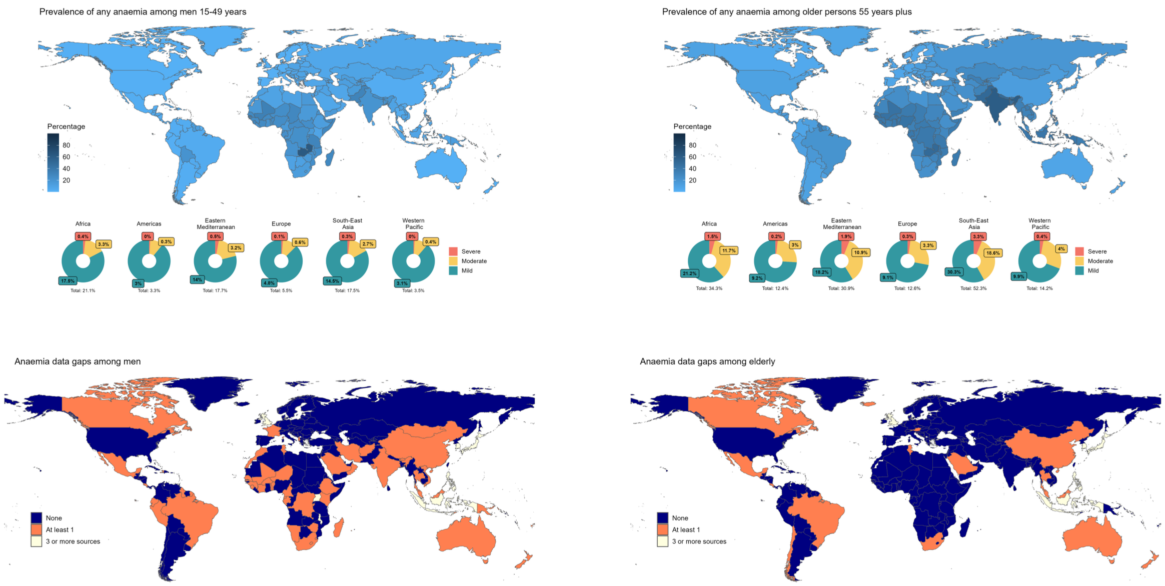

Key (for prevalence): 60, 40, 20.

Key (for gaps): No anaemia data; At least one source (1-2 sources); Three or more sources;

Footnote: Haemoglobin, Hb. Hb <130 g/L in men aged 15-49 years and in men aged 55 and above; Hb <120 g/L in women aged 55 and above. Data estimates derived from GBD 2023. Estimates are not yet available based on the newly recommended WHO cutoffs and adjustments.[1]

Footnote (for gaps): Data from the Vitamin and Mineral Nutrition Information System (VMNIS) between 2000-2020. Age intervals defined based on WHO's VMNIS classification of population groups and do not exactly cover the age ranges of interest: men 10-101 years and older persons 50-101 years. The most common age grouping for each population is 14-49 years for men (31%) and 60-69 years for older persons (45%).

Table 1: Commonalities and divergences in methodology for estimating anaemia prevalence between Global Burden of Disease (GBD) and the World Health Organization (WHO).

**Methodology**	**GBD**	**WHO**
Survey selection criteria	"Population-based sample or group judged to adequately represent the sex, age groups, and location of the study" with the majority of sources from the Vitamin and Mineral Nutrition Information System (VMNIS)	Representative at the national level or at least three first administrative units within the country. Taken from the VMNIS.
Statistical model	Spatio-Temporal Gaussian Process Regression modelling	Bayesian hierarchical mixture modelling
Covariates for imputing estimates when data is sparse or inconsistent	50th percentile of haemoglobin, haemoglobin C trait, haemoglobin S trait, healthcare access and quality index, malaria incidence, modern contraception, summary exposure value of child underweight and child wasting, summary exposure value of impaired kidney function, and sociodemographic index. Selection of covariates varied based on model performance and the regression coefficients were statistically significant ($p < 0{\cdot}05$) and were in the expected direction.	Socio-demographic index, meat supply (kcal/capita), mean BMI (only for women), and log of baseline under-five mortality adjusted to exclude sudden events e.g. natural disasters (only for children).

Major data gaps in anaemia, and its causes in population-based surveys.

We exported the Vitamin and Mineral Nutrition Information System (VMNIS) database for the haemoglobin indicator on the 26th of February 2024, with the filter logic being representativeness is 'national' and year is greater than or equal to 2000 and is less than or equal to 2024. We cross-checked the VMNIS survey identification number in this database with a database provided by the Institute for Health Metrics and Evaluation (IHME) and WHO and formed our base dataset - hereinafter 'anaemia database'.

For calculation of data gaps by population group, we used the WHO population definition in VMNIS. For each population group (infants, pre-school children, school-aged children, adolescents, pregnant women, non-pregnant women, men, and elderly), we identified countries that have no survey, at least one survey, or three or more surveys recorded in the anaemia database.

Micronutrient databases merge

We also exported VMNIS micronutrient databases for all indicators listed below **(Table 2)** individually, hereinafter 'individual indicator database'. We first merged each individual indicator database of the same micronutrient into its 'individual micronutrient database'. We then merged each individual micronutrient database with the anaemia database by the VMNIS survey identification number.

Table 2: Micronutrient and indicator databases.

Micronutrient	Indicator
Folate	Folate (red blood cell)
Folate	Folate (plasma or serum)
Iron	Ferritin
Iron	Serum transferrin receptor
Iron	Body iron stores
Riboflavin (vitamin B2)	Erythrocyte Glutathione Reductase Activity Coefficient (EGRAC)
Vitamin A	Modified Relative Dose Response test (MRDR)
Vitamin A	Retinol (plasma or serum)
Vitamin A	Retinol binding protein
Vitamin B12	Vitamin B12

Non-nutrition causes - data extraction strategy:
We searched the causes of anaemia in the surveys listed in our anaemia database, using the search terms under each cause **(Table 3)**. We excluded search words that were: i) only mentioned in the "background" (but with no data related to causes being collected), or ii) related to awareness/education, or iii) related to vaccines but not the diseases themselves.

Table 3: Search terms for non-nutritional anaemia causes.

**Category**	**Causes**	**Search Terms**
Chronic	Cancer	Cancer, chemotherapy (cancer-related), radiation therapy (cancer-related)
Chronic	Diabetes	Diabetes, Diabetic, Haemoglobin A1C test, Haemoglobin A1C, HbA1c
Chronic	Kidney disease	Kidney disease, CKD, renal disease
Chronic	Obesity	Obesity, Obese, overweight, body mass index, BMI
Chronic	Gastrointestinal disease	Gastrointestinal disease(s)
Infection	HIV/AIDS	HIV, AIDS, human immunodeficiency virus, acquired immunodeficiency syndrome
Infection	Malaria	Malaria, *Plasmodium falciparum (P. falciparum), Plasmodium vivax (P. vivax), Plasmodium malariae (P. malariae), Plasmodium ovale (P. ovale), Plasmodium knowlesi (P. knowlesi)*
Infection	Parasitic worms	Parasitic worms, Roundworm, whipworm, hookworm); *strongyloides stercoralis*, schistosomiasis
Infection	Tuberculosis	Tuberculosis, *Mycobacterium tuberculosis*, TB
Infection	Visceral leishmaniasis	Visceral leishmaniasis
Infection	Helicobacter pylori	Helicobacter pylori, H. pylori
Infection	Salmonella	Salmonella
Infection	Other viruses	Virus

Inflammation	Alpha-1 acid glycoprotein	Alpha-1 acid glycoprotein, α−1 acid glycoprotein, AGP
Inflammation	C-reactive protein	C-reactive protein, CRP
Gynaecological and obstetric conditions	Heavy menstrual bleeding	Heavy menstrual bleeding; Menorrhagia
Gynaecological and obstetric conditions	Haemorrhage	Antepartum haemorrhage, postpartum haemorrhage, or Haemorrhage/Haemorrhage related to delivery, Bleeding
Gynaecological and obstetric conditions	Unnecessary caesarean section	Caesarean; Caesarean; C-section
Inherited red blood cell disorders	α-thalassemia	Alpha-thalassemia, Alpha-thalassaemia, α-thalassaemia, α-thalassemia major, include α-thalassemia minor or carrier
Inherited red blood cell disorders	β-thalassemia	Beta thalassaemia, Beta thalassemia, Beta thalassemia major and minor; Cooley's anaemia, Cooley's anaemia
Inherited red blood cell disorders	Sickle cell disorders	Sickle cell disease(s)/disorder(s), SCD; HbS beta thalassemia, HbSS, HbSC
Inherited red blood cell disorders	Haemoglobin H disease	Haemoglobin H disease; HbH, Haemoglobin H disease
Inherited red blood cell disorders	Glucose-6-phosphate dehydrogenase deficiency	G6PD; Glucose-6-phosphate dehydrogenase
Inherited red blood cell disorders	South-East Asian ovalocytosis	South-East Asian ovalocytosis, SEA ovalocytosis; SAO

Table 4: Number of population-based surveys that measured the number of causes[#] among surveys that collected anaemia data between 2000 and 2020, by region.

**Number of causes**	**Global n/N (%)**	**African n/N (%)**	**Americas n/N (%)**	**Eastern Mediterranean n/N (%)**	**European n/N (%)**	**South-East Asia n/N (%)**	**Western Pacific n/N (%)**
Zero cause	47/407 (12)	16/148 (11)	2/67 (3)	8/47 (17)	1/44 (2)	8/46 (17)	12/55 (22)
One cause	122/407 (30)	42/148 (28)	13/67 (19)	13/47 (28)	15/44 (34)	18/46 (39)	21/55 (38)
Two causes	115/407 (28)	19/148 (13)	36/67 (54)	17/47 (36)	17/44 (39)	13/46 (28)	13/55 (24)
Three causes	109/407 (27)	65/148 (44)	16/67 (24)	5/47 (11)	10/44 (23)	6/46 (13)	7/55 (13)
Four causes	11/407 (3)	5/148 (3)	0/67 (0)	4/47 (9)	1/44 (2)	0/46 (0)	1/55 (2)
Five causes	3/407 (1)	1/148 (1)	0/67 (0)	0/47 (0)	0/44 (0)	1/46 (2)	1/55 (2)
Six causes	0/407 (0)	0/148 (0)	0/67 (0)	0/47 (0)	0/44 (0)	0/46 (0)	0/55 (0)

[#]Causes include chronic diseases, micronutrient status, inherited red blood cell disorders, gynaecological and obstetric conditions, infections, and inflammation. Denominators include all surveys in the analysis.

Analytical method considerations for haemoglobin measurement.

Box 1: An example of measuring haemoglobin using venous blood on an automated haematology analyser accompanied by high-quality control measures.

The National Health and Nutrition Examination Survey (NHANES) has been assessing the health and nutritional status of adults and children in the United States since the 1960s. Participants are interviewed in their households and referred to a mobile exam centre. A full blood count (FBC), including haemoglobin, is completed on-site with participants who are aged one year and above. A phlebotomist draws venous samples and immediately transfers the specimens to a laboratory technician who performs a FBC in duplicate with a CoulterDxH 800 Analyser. Quality control materials are run daily on instruments. External quality control includes participation in an interlaboratory comparison quality assurance programme and proficiency testing through the College of American Pathologists.[2]

Establishing new or strengthening existing survey platforms

Table 5*:* Comparison of Survey Platforms for collecting data on anaemia and its causes.

Survey platform	Advantages	Disadvantages
**Standalone nutrition survey** – Purpose-specific survey, usually focused on collecting nutritional status including anthropometry, anaemia and micronutrient status but could be expanded to other causes	• Survey activities tailored to specific data, and no constraints related to requirements for other indicators<br>• Reduced survey timeline under ideal conditions<br>• High data quality if have well-established quality assurance and control<br>• Respond flexibly and quickly to emerging data needs	• Fewer opportunities for standalone surveys than multi-topic surveys<br>• No global survey platform for collecting this type of data<br>• Increased burden on countries and duplication of efforts<br>• Lack of harmonisation in data collection and reporting<br>• Microdata not usually accessible<br>• Expensive and no clear donor
**Multi-topic survey** – Collects data on a wide set of topics of national and global public health importance. Under a 'piggyback' approach teams follow the main survey team to collect data often in a subset of clusters. Under a fully integrated approach team is moving together to collect the data in all clusters although usually in a subsample of households.	• Global survey platforms well-established offering potential for many data collection opportunities<br>• Robust system in place for survey design and preparation, training and data collection, data processing, and dissemination<br>• Standardised survey design, methodologies, and indicator definitions<br>• Increased data comparability between countries and overtime<br>• Serve more than one function can reduce burden on countries and duplication<br>• Microdata typically open access, easily accessible, and well documented<br>• Data linkage to a range of indicators<br>• Piggybacking is more cost-efficient than standalone	• 'Piggyback' approach is the most logistically complex, followed by full integration, compared to a standalone survey<br>• Typically, added to questionnaire-based surveys and require infrastructure changes, such as a cold chain<br>• Increased length of fieldwork to the survey<br>• May negatively impact the quality of the additional data or the other components of the survey because of focus on multiple types of data<br>• Survey timelines not always aligned (e.g., laboratory testing can take longer than processing other survey data)<br>• Full integration is expensive due to the need for more data collectors (and vehicles) than in either a piggyback or standalone approach

**Surveillance system** – Ongoing collection at regular intervals, each round can be in the same population or in different population[#]	• Long-term and timely tracking on a regular basis<br>• Can vary the type of data collected between each cycle<br>• Opportunity for continual quality improvement<br>• Workflow is continual which helps with sustainability, staff retention, and steady source of funding	• Less expensive than standalone or integrated, and potentially less expensive than piggyback approach<br>• Often requires significant effort to institutionalise<br>• Political or funding changes can disrupt the system

[#] Surveillance system can be a standalone or multi-topic surveillance system with similar associated advantages and disadvantages.

Recent changes in the definition of anaemia are expected to affect estimates of anaemia.

Table 6: Comparison of anaemia prevalence (%) between the new and old cutoffs and altitude adjustment among children aged 6-59 months; and between the old and new altitude and smoking adjustment among non-pregnant women aged 15-49 years, five surveys in African region.[#]

Adjustment	Senegal 2017 <500 m	Eswatini 2006-07 500-999m	Zambia 2018 1000-1499 m	Lesotho 2014 1500-1999m	Ethiopia 2016 2000+m
**Children**	N=10,592	N=2,466	N=8,530	N=1,689	N=8,451
**Old cutoff and old altitude adjustment**	70·8 (69·6, 72·1)	40·4 (38·0, 42·8)	58·0 (56·5, 59·5)	50·7 (47·5, 53·9)	56·9 (54·4, 59·4)
**New cutoff and new altitude adjustment**	66·9 (65·5, 68·2)	47·5 (45·0, 49·9)	70·3 (69·0, 71·7)	61·3 (58·3, 64·3)	66·9 (64·6, 69·3)
**New cutoff and old altitude adjustment**	66·9 (65·5, 68·2)	35·7 (33·4, 38·0)	52·9 (51·4, 54·4)	47·4 (44·1, 50·6)	52·6 (50·0, 55·3)
**Old cutoff and new altitude adjustment**	70·8 (69·6, 72·1)	52·0 (49·5, 54·5)	74·0 (72·7, 75·3)	64·4 (61·6, 67·3)	70·3 (68·1, 72·5)
**Women**	N=7,195	N=4,299	N=12,112	N=3,109	N= 13,440
**Old altitude and smoking adjustment**	53·4 (51·4, 55·4)	29·2 (27·5, 30·9)	30·2 (28·8, 31·5)	27·2 (25·1, 29·2)	23·2 (21·5, 24·8)
**New altitude and smoking adjustment**	53·4 (51·4, 55·4)	37·9 (36·0, 39·8)	43·7 (42·2, 45·3)	38·3 (36·2, 40·4)	33·3 (31·4, 35·1)

[#]New cutoff defined as Hb<105 g/L for children aged 6-23 months and Hb<110 g/L for children aged 24-59 months; Old cutoff defined as Hb<110 g/L for children aged 6-59 months. New altitude adjustment defined as Hb adjustment (g/L) = (0·0056384 x elevation in meters) + (0·0000003 x elevation in meters^2); Old altitude adjustment defined as Hb adjustment (g/L) = - 0·32 x (0·0032808 x elevation in meters) + 0·22 x (0·0032808 x elevation in meters) ^2. New smoking adjustment defined as Hb adjustment (g/L) = (0·4565 x cigarette number per day) + (−0·0078 x cigarette number per day^2).Old smoking adjustment defined by number of cigarettes per day: <10 (no adjustment), 10-19 (-3 g/L), 20-39 (-5 g/L), 40 or more (-7 g/L), unknown quantity or non-cigarettes smoking (-3 g/L). Sample was limited to the legally recognised resident population. Countries were selected based on the highest percentage of the population residing at different altitudes.[3] If two countries met the criteria, one was selected randomly. If the same country had the highest percentage of the population at consecutive altitude bins, the altitude bins were combined. Data was restricted to countries with publicly available microdata for children and women. Numbers in brackets represent Wald confidence limits of the percentage.

Table 7: Comparison of anaemia prevalence (%) between the new and old cutoffs and altitude adjustment among children aged 6-59 months and between the old and new altitude and smoking adjustment among non-pregnant women aged 15-49 years, five surveys in the region of the Americas.[#]

**Adjustment**	**Guyana <500m**	**Honduras 500-999m**	**Guatemala 1000-2500m**	**Bolivia 2500-2999m**	**Peru 3000+m**
**Children**	N=1,601	N=9,252	N=10,803	N=2,535	N=8,626
**Old cutoff and old altitude adjustment**	38·8 (35·1, 42·6)	29·1 (27·7, 30·4)	32·3 (30·9, 33·7)	60·4 (57·5, 63·2)	32·1 (30·6, 33·5)
**New cutoff and new altitude adjustment**	36·9 (33·2, 40·7)	33·6 (32·2, 34·8)	40·0 (38·6, 41·4)	58·2 (55·5, 61·0)	30·9 (29·4, 32·3)
**New cutoff and old altitude adjustment**	33·8 (30·4, 37·1)	24·0 (22·7, 25·2)	26·8 (25·5,28·2)	57·1 (54·2, 60·0)	26·7 (25·3, 28·1)
**Old cutoff and new altitude adjustment**	41·3 (37·3, 45·4)	39·4 (38·0, 40·7)	45·5 (44·1, 46·9)	61·8 (59·2, 64·4)	35·8 (34·3, 37·2)
**Women**	N=4,254	N=20,571	N=24,106	N=5,486	N=22,807
**Old altitude and smoking adjustment**	37·9 (35·9, 40·0)	14·8 (14·2, 15·5)	13·1 (12·4, 13·7)	37·4 (35·6, 39·3)	17·1 (16·4, 17·9)
**New altitude and smoking adjustment**	40·1 (38·0, 42·1)	20·8 (20·0, 21·7)	20·2 (19·4, 21·0)	38·4 (36·5, 40·2)	19·4 (18·7, 20·2)

[#]New cutoff defined as Hb<105 g/L for children aged 6-23 months and Hb<110 g/L for children aged 24-59 months; Old cutoff defined as Hb<110 g/L for children aged 6-59 months. New altitude adjustment defined as Hb adjustment (g/L) = (0·0056384 x elevation in meters) + (0·0000003 x elevation in meters^2); Old altitude adjustment defined as Hb adjustment (g/L) = - 0·32 x (0·0032808 x elevation in meters) + 0·22 x (0·0032808 x elevation in meters)^2. New smoking adjustment defined as Hb adjustment (g/L) = (0·4565 x cigarette number per day) + (−0·0078 x cigarette number per day^2). Old smoking adjustment defined by number of cigarettes per day: <10 (no adjustment), 10-19 (-3 g/L), 20-39 (-5 g/L), 40 or more (-7 g/L), unknown quantity or non-cigarettes smoking (-3 g/L). Sample was limited to the legally recognised resident population. Countries were selected based on the highest percentage of the population residing at different altitudes.[3] If two countries met the criteria, one was selected randomly. If the same country had the highest percentage of the population at consecutive altitude bins, the altitude bins were combined. Data was restricted to countries with publicly available microdata for children and women. Numbers in brackets represent Wald confidence limits of the percentage.

Table 8: Comparison of anaemia prevalence (%) between the new and old cutoffs and altitude adjustment among children aged 6-59 months and between the old and new altitude and smoking adjustment among non-pregnant women aged 15-49 years, three surveys in the Southeast Asian region.[#]

Adjustment	Bangladesh <500m	Timor-Leste 500-999m	Nepal 1000+m
**Children**	N=2,242	N=2,026	N=2,328
**Old cutoff and old altitude adjustment**	51·1 (48·6, 53·7)	40·4 (37·3, 43·5)	43·1 (40·5, 45·6)
**New cutoff and new altitude adjustment**	49·4 (47·0, 51·9)	47·3 (44·1, 50·5)	46·9 (44·2, 49·6)
**New cutoff and old altitude adjustment**	46·1 (43·6, 48·7)	35·7 (32·8, 38·6)	38·5 (35·8, 41·1)
**Old cutoff and new altitude adjustment**	54·7 (52·3, 57·1)	52·1 (48·9, 55·2)	51·2 (48·6, 53·9)
**Women**	N=5,150	N=4,062	N=6,916
**Old altitude and smoking adjustment**	41·7 (39·9, 43·5)	21·6 (20·0, 23·2)	33·8 (31·9, 35·6)
**New altitude and smoking adjustment**	44·6 (42·7, 46·4)	31·7 (29·8, 33·6)	41·5 (39·7, 43·3)

[#]New cutoff defined as Hb<105 g/L for children aged 6-23 months and Hb<110 g/L for children aged 24-59 months; Old cutoff defined as Hb<110 g/L for children aged 6-59 months. New altitude adjustment defined as Hb adjustment (g/L) = (0·0056384 x elevation in meters) + (0·0000003 x elevation in meters^2); Old altitude adjustment defined as Hb adjustment (g/L) = - 0·32 x (0·0032808 x elevation in meters) + 0·22 x (0·0032808 x elevation in meters)^2. New smoking adjustment defined as Hb adjustment (g/L) = (0·4565 x cigarette number per day) + (−0·0078 x cigarette number per day^2). Old smoking adjustment defined by number of cigarettes per day: <10 (no adjustment), 10-19 (-3 g/L), 20-39 (-5 g/L), 40 or more (-7 g/L), unknown quantity or non-cigarettes smoking (-3 g/L). Sample was limited to the legally recognised resident population. Countries were selected based on the highest percentage of the population residing at different altitudes.[3] If two countries met the criteria, one was selected randomly. If the same country had the highest percentage of the population at consecutive altitude bins, the altitude bins were combined. Data was restricted to countries with publicly available microdata for children and women. Numbers in brackets represent Wald confidence limits of the percentage.

Figure 3: A) Comparison of anaemia prevalence (%) between the new and old cutoffs and altitude adjustment among children aged 6-59 months in the American Region and in the Southeast Asian Region. B) Comparison of anaemia prevalence (%) between the old and new altitude and smoking adjustment among non-pregnant women aged 15-49 years in the American Region and in the Southeast Asian Region. Error bars denote Wald confidence limits of the percentage.

A)

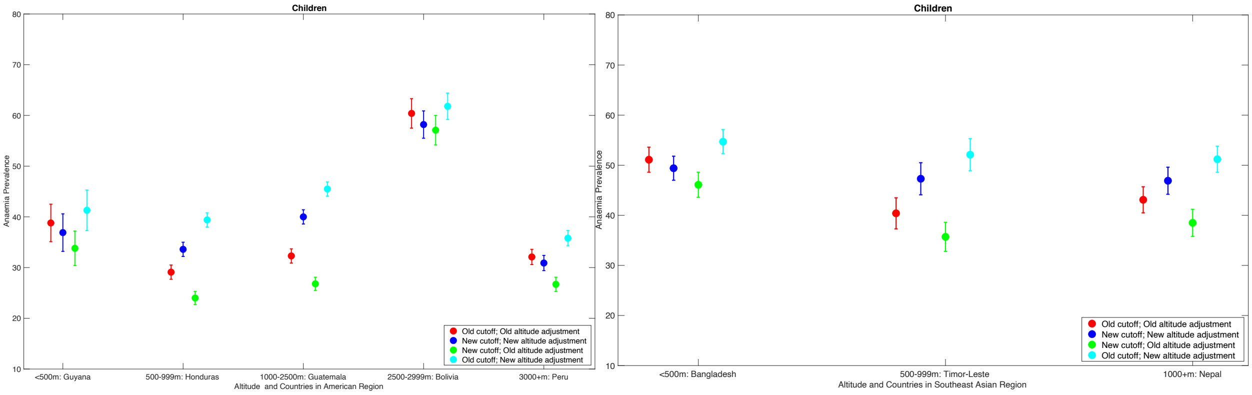

B)

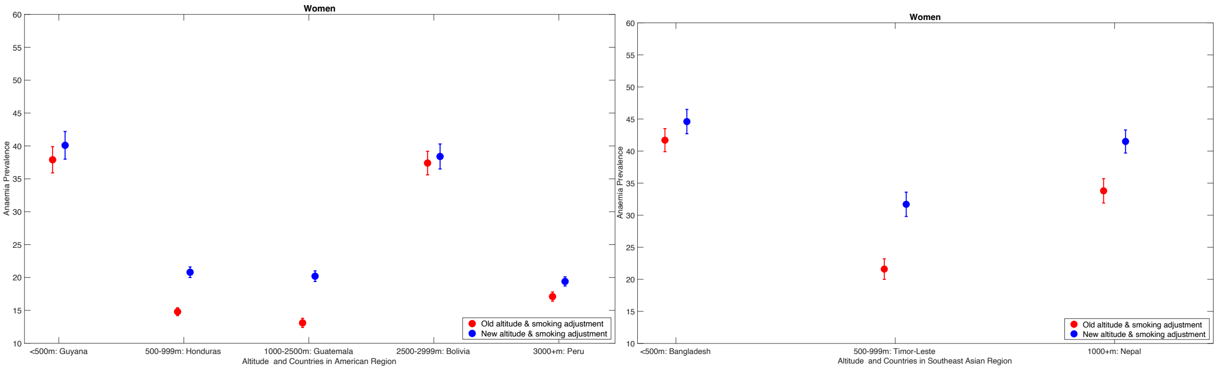

Section 3: Improving anaemia control programs: implementation and governance.

Current state of anaemia governance and implementation

Table 9: Anaemia-related Sustainable Development Goals (SDGs) (2015-2030)

1. SDG 2: Zero Hunger - Anaemia often stems from inadequate nutrition, particularly iron-deficiency anaemia. SDG 2 aims to end hunger, achieve food security, improve nutrition, and promote sustainable agriculture. By focusing on improving food availability, access, and quality, SDG 2 can contribute to reducing anaemia. Specifically, under SDG 2 Target 2·2 (to end all forms of malnutrition by 2030), prevalence of anaemia in women 15-49 years of age, by pregnancy status, is listed as indicator 2·2·3, which aligns with the GNT 2 (50% reduction in WRA anaemia prevalence).
2. SDG 3: Good Health and Well-being - Anaemia affects overall health and well-being, and SDG 3 aims to ensure healthy lives and well-being for all at all ages. Specifically, target 3·2 aims to end preventable deaths of newborns and children under five, which includes addressing anaemia as a significant contributor to child mortality.
3. SDG 4: Quality Education - Anaemia can impact cognitive development and impair learning abilities in children. SDG 4 focuses on ensuring inclusive and equitable quality education for all. Addressing anaemia can contribute to improving the learning outcomes and overall educational attainment of children.
4. SDG 5: Gender Equality - Women and girls are particularly affected by anaemia due to factors such as menstruation, pregnancy, and inadequate nutrition. SDG 5 aims to achieve gender equality and empower all women and girls. Addressing anaemia is crucial to ensuring the well-being and empowerment of women and girls.
5. SDG 10: Reduced Inequalities - Anaemia disproportionately affects vulnerable populations, including those in low-income countries and marginalised communities. SDG 10 focuses on reducing inequalities within and among countries. Addressing anaemia contributes to reducing health disparities and promoting equal access to healthcare services.
6. SDG 17: Partnerships for the Goals - Combating anaemia requires multi-stakeholder collaborations and partnerships. SDG 17 emphasises the importance of global partnerships for sustainable development. Collaborations among governments, healthcare providers, NGOs, research institutions, and the private sector are crucial for developing and implementing effective strategies to address anaemia on a global scale.

Additional references for the Commission

Box 1

Sabet CJ. Medical research funding cuts will save money and spend lives. *The Lancet*. 2025;**405**(10487):1337-8.

Section 1: The global burden of anaemia: uncovering data gaps and challenges.

Effects of revised anaemia definitions on prevalence estimates

Sachdev HS, Porwal A, Acharya R, et al. Haemoglobin thresholds to define anaemia in a national sample of healthy children and adolescents aged 1-19 years in India: a population-based study. *The Lancet Global Health* 2021; **9**(6): e822-e31.

Addo OY, Yu EX, Williams AM, et al. Evaluation of Hemoglobin Cutoff Levels to Define Anemia Among Healthy Individuals. *JAMA Network Open* 2021; **4**(2574-3805 (Electronic)).

Enhancing data quality: validity and reliability of anaemia estimates

The DHS Program. Anemia Estimates Using Venous and Capillary Blood Samples in the 2019–20 Rwanda DHS. Accessed April 2024. 2023

The DHS Program. Anaemia Estimates using Venous and Capillary Blood Samples in the 2022 Tanzania DHS-MIS. 2023.

De la Cruz-Góngora V, Méndez-Gómez-Humarán I, Gaona-Pineda EB, Shamah-Levy T, Dary O. Drops of capillary blood are not appropriate for hemoglobin measurement with point-of-care devices: A comparative study using drop capillary, pooled capillary, and venous blood samples. *Nutrients* 2022; **14**(24): 5346.

Hackl LS, Karakochuk CD, Mazariegos DI, et al. Assessing Accuracy and Precision of Hemoglobin Determination in Venous, Capillary Pool, and Single-Drop Capillary Blood Specimens Using three Different HemoCue® Hb Models: The Multicountry Hemoglobin Measurement (HEME) Study. *The Journal of Nutrition* 2024.

Ayuen DS, Olupot-Olupot P, Muhindo R, et al. Comparing HemoCue® and Quantitative Buffy Coat® and Coulter Counter-measured haemoglobin concentrations in African children with acute uncomplicated malaria: a Bland–Altman analysis. *Malaria Journal* 2025; **24**(1): 77.

Mannino RG, Myers DR, Tyburski EA, et al. Smartphone app for non-invasive detection of anemia using only patient-sourced photos. *Nature Communications* 2018; **9**(1): 4924.

Mitani A, Huang A, Venugopalan S, et al. Detection of anaemia from retinal fundus images via deep learning. *Nature Biomedical Engineering* 2020; **4**(1): 18-27.

Khan R, Maseedupally V, Thakoor KA, Raman R, Roy M. Noninvasive Anemia Detection and Hemoglobin Estimation from Retinal Images Using Deep Learning: A Scalable Solution for Resource-Limited Settings. *Translational Vision Science & Technology* 2025; **14**(1): 20-.

Analytical method considerations for haemoglobin measurement

Larson LM, Braat S, Hasan MI, et al. Preanalytic and analytic factors affecting the measurement of haemoglobin concentration: impact on global estimates of anaemia prevalence. *BMJ Global Health* 2021; **6**(7): e005756.

Establishing new or strengthening existing survey platforms

NIPN Uganda. Towards a Comprehensive Monitoring and Assessment of the NUTRITION SITUATION IN UGANDA - A Nutrition Data Landscape Report of Government Resources, 2020.

Using data on anaemia causes to develop context-specific solutions

Hess SY, McLain AC, Frongillo EA, et al. Challenges for estimating the global prevalence of micronutrient deficiencies and related disease burden: a case study of the global burden of disease study. *Current Developments in Nutrition* 2021; **5**(12): nzab141.

Ferrucci L, Fabbri E. Inflammageing: chronic inflammation in ageing, cardiovascular disease, and frailty. *Nature Reviews Cardiology* 2018; **15**(9): 505-22.

Section 2 – Anaemia control must consider its complex multifactorial causes

Physiological iron needs by life stages

Petry N, Olofin I, Hurrell RF, et al. The proportion of anemia associated with iron deficiency in low, medium, and high human development index countries: a systematic analysis of national surveys. *Nutrients* 2016; **8**(11): 693.

Wirth JP, Woodruff BA, Engle-Stone R, et al. Predictors of anemia in women of reproductive age: Biomarkers Reflecting Inflammation and Nutritional Determinants of Anemia (BRINDA) project. *The American journal of clinical nutrition* 2017; **106**: 416S-27S.

Brittenham GM, Moir-Meyer G, Abuga KM, et al. Biology of anemia: a public health perspective. *The Journal of Nutrition* 2023; **153**: S7-S28.

Pasricha S-R, Tye-Din J, Muckenthaler MU, Swinkels DW. Iron deficiency. *The Lancet* 2021; **397**(10270): 233-48.

Allen LH. Anemia and iron deficiency: effects on pregnancy outcome. *The American journal of clinical nutrition* 2000; **71**(5): 1280S-4S.

Aher S, Malwatkar K, Kadam S. Neonatal anemia. *Seminars in Fetal and Neonatal Medicine* 2008; **13**(4): 239-47.

Mancera-Soto E, Ramos-Caballero DM, Magalhaes J, Chaves Gomez S, Schmidt WF, Cristancho-Mejía E. Quantification of testosterone-dependent erythropoiesis during male puberty. *Experimental Physiology* 2021; **106**(7): 1470-81.

Cotter J, Baldaia C, Ferreira M, Macedo G, Pedroto I. Diagnosis and treatment of iron-deficiency anemia in gastrointestinal bleeding: A systematic review. *World J Gastroenterol* 2020; **26**(45): 7242-57.

Role of micronutrient deficiencies in the development of anaemia

Balarajan Y, Ramakrishnan U, Özaltin E, Shankar AH, Subramanian S. Anaemia in low-income and middle-income countries. *The lancet* 2011; **378**(9809): 2123-35.

Table 2:

Additional references for Vitamin A (retinol)[1, 2]; Vitamin B2 (riboflavin)[1,3]; Vitamin B6 (pyridoxine)[2,4]; Vitamin B9 (folate/ folic acid)[2]; Vitamin B12 (cobalamin)[1,2]; Vitamin C (ascorbic acid)[2]; Vitamin D (calciferol)[5,6]; Vitamin E (tocopherol)[1]; Copper[4,7,30]; Calcium[8,9]; Zinc[10]

1. Fishman SM, Christian P, West KP. The role of vitamins in the prevention and control of anaemia. *Public health nutrition* 2000; **3**(2): 125-50.
2. Semba R, Bloem M. The anemia of vitamin A deficiency: epidemiology and pathogenesis. *European journal of clinical nutrition* 2002; **56**(4): 271-81.
3. Powers HJ. Riboflavin (vitamin B-2) and health. *The American journal of clinical nutrition* 2003; **77**(6): 1352-60.
4. Karakochuk CD, Zimmermann MB, Moretti D, Kraemer K. Nutritional Anemia: Springer International Publishing; 2022.
5. Bacchetta J, Zaritsky JJ, Sea JL, et al. Suppression of iron-regulatory hepcidin by vitamin D. *Journal of the American Society of Nephrology* 2014; **25**(3): 564-72.
6. Mogire RM, Muriuki JM, Morovat A, et al. Vitamin D Deficiency and Its Association with Iron Deficiency in African Children. *Nutrients*, 2022. (accessed.)
7. Knovich MA, Il'yasova D, Ivanova A, Molnár I. The association between serum copper and anaemia in the adult Second National Health and Nutrition Examination Survey (NHANES II) population. *British journal of nutrition* 2008; **99**(6): 1226-9.
8. Krebs J, Agellon LB, Michalak M. Ca2+ homeostasis and endoplasmic reticulum (ER) stress: An integrated view of calcium signaling. *Biochemical and biophysical research communications* 2015; **460**(1): 114-21.
9. Miller BA, Cheung JY. Mechanisms of erythropoietin signal transduction: involvement of calcium channels. *Proceedings of the Society for Experimental Biology and Medicine* 1994; **206**(3): 263-7.
10. Killilea DW, Siekmann JH. The Role of Zinc in the Etiology of Anemia. In: Karakochuk CD, Zimmermann MB, Moretti D, Kraemer K, eds. Nutritional Anemia. Cham: Springer International Publishing; 2022: 187-94.

Dietary determinants of iron deficiency and anaemia

Dietary iron adequacy and bioavailability

Donangelo CM, Woodhouse LR, King SM, Viteri FE, King JC. Supplemental Zinc Lowers Measures of Iron Status in Young Women with Low Iron Reserves. *The Journal of Nutrition* 2002; **132**(7): 1860-4.

Hallberg L, Hulthén L. Prediction of dietary iron absorption: an algorithm for calculating absorption and bioavailability of dietary iron. *The American journal of clinical nutrition* 2000; **71**(5): 1147-60.

Dietary patterns and iron deficiency and anaemia

Ferguson EL, Darmon N, Fahmida U, Fitriyanti S, Harper TB, Premachandra IM. Design of optimal food-based complementary feeding recommendations and identification of key "problem nutrients" using goal programming. *The Journal of nutrition* 2006; **136**(9): 2399-404.

Gorst-Rasmussen A, Dahm CC, Dethlefsen C, Scheike T, Overvad K. Exploring Dietary Patterns By Using the Treelet Transform. *American Journal of Epidemiology* 2011; **173**(10): 1097-104.

Li T, Guan L, Wang X, et al. Relationship Between Dietary Patterns and Chronic Diseases in Rural Population: Management Plays an Important Role in the Link. *Front Nutr* 2022; **9**: 866400.

Haider LM, Schwingshackl L, Hoffmann G, Ekmekcioglu C. The effect of vegetarian diets on iron status in adults: A systematic review and meta-analysis. *Critical reviews in food science and nutrition* 2018; **58**(8): 1359-74.

Co-occurrence of anaemia and malnutrition

Rahman MS, Mushfiquee M, Masud MS, Howlader T. Association between malnutrition and anemia in under-five children and women of reproductive age: Evidence from Bangladesh Demographic and Health Survey 2011. *PloS one* 2019; **14**(7): e0219170.

Rytter MJH, Kolte L, Briend A, Friis H, Christensen VB. The immune system in children with malnutrition—a systematic review. *PloS one* 2014; **9**(8): e105017.

Nutritional interventions addressing anaemia:

Oral iron supplementation

World Health Organization. Use of multiple micronutrient powders for point-of-use fortification of foods consumed by infants and young children aged 6–23 months and children aged 2–12 years. Geneva, Switzerland, 2016.

World Health Organization. Guideline: Intermittent iron and folic acid supplementation in menstruating women, 2011.

World Health Organization. Daily iron supplementation in infants and children. Geneva, Switzerland; 2016.

Multinutrient supplementation to tackle micronutrient deficiencies known to cause anaemia

World Health Organization. (2007). Standards for maternal and neonatal care. World Health Organization. https://iris.who.int/handle/10665/69735

Ajayi OA, Nnaji UR. Effect of ascorbic acid supplementation on haematological response and ascorbic acid status of young female adults. *Annals of nutrition and metabolism* 1990; **34**(1): 32-6.

Seshadri S, Shah A, Bhade S. Haematologic response of anaemic preschool children to ascorbic acid supplementation. *Human nutrition Applied nutrition* 1985; **39**(2): 151-4.

Industrial food fortification

Dewi NU, Mahmudiono T. Effectiveness of food fortification in improving nutritional status of mothers and children in Indonesia. *International journal of environmental research and public health* 2021; **18**(4): 2133.

Peña-Rosas JP, Mithra P, Unnikrishnan B, et al. Fortification of rice with vitamins and minerals for addressing micronutrient malnutrition. *Cochrane Database Syst Rev* 2019; **2019**(10).

Food preparation techniques to improve nutrient intake

Anaemene D, Fadupin G. Anti-nutrient reduction and nutrient retention capacity of fermentation, germination and combined germination-fermentation in legume processing. *Applied Food Research* 2022; **2**(1): 100059.

Charles CV, Dewey CE, Daniell WE, Summerlee AJ. Iron-deficiency anaemia in rural Cambodia: community trial of a novel iron supplementation technique. *European journal of public health* 2011; **21**(1): 43-8.

Agriculture-nutrition programmes

Baliki G, Weiffen D, Schreinemachers P, et al. Effect of an Integrated School Garden and Home Garden Intervention on Anemia Among School-Aged Children in Nepal: Evidence From a Cluster Randomised Controlled Trial. *Food and Nutrition Bulletin* 2023; **44**(3): 195-206.

Michaux KD, Hou K, Karakochuk CD, et al. Effect of enhanced homestead food production on anaemia among Cambodian women and children: A cluster randomized controlled trial. *Maternal & Child Nutrition* 2019; **15**: e12757.

Osei A, Pandey P, Nielsen J, et al. Combining home garden, poultry, and nutrition education program targeted to families with young children improved anemia among children and anemia and underweight among nonpregnant women in Nepal. *Food and nutrition bulletin* 2017; **38**(1): 49-64.

Fraeye I, Kratka M, Vandenburgh H, Thorrez L. Sensorial and nutritional aspects of cultured meat in comparison to traditional meat: much to be inferred. *Frontiers in nutrition* 2020; **7**: 35.

Nutritional education and awareness

Kyere P, Veerman JL, Lee P, Stewart DE. Effectiveness of school-based nutrition interventions in sub-Saharan Africa: a systematic review. *Public Health Nutrition* 2020; **23**(14): 2626-36.

Lua PL, Elena WDWP. The impact of nutrition education interventions on the dietary habits of college students in developed nations: a brief review. *The Malaysian journal of medical sciences: MJMS* 2012; **19**(1): 4.

Kaya Kaçar H, Kaçar ÖF, McCullough F. Nutrition Messaging by Healthcare Students: A Mixed-Methods Study Exploring Social Media Usage and Digital Competence. *Nutrients*, 2024. (accessed.

Other interventions addressing anaemia:

Optimising iron stores through delayed cord clamping

Farrar D, Airey R, Law GR, Tuffnell D, Cattle B, Duley L. Measuring placental transfusion for term births: weighing babies with cord intact. *BJOG* 2011; **118**(1): 70-5.

Mercer JS. Current best evidence: a review of the literature on umbilical cord clamping. *J Midwifery Womens Health* 2001; **46**(6): 402-14.

Rabe H, Mercer J, Erickson-Owens D. What does the evidence tell us? Revisiting optimal cord management at the time of birth. *Eur J Pediatr* 2022; **181**(5): 1797-807

Dipak NK, Nanavat RN, Kabra NK, Srinivasan A, Ananthan A. Effect of Delayed Cord Clamping on Hematocrit, and Thermal and Hemodynamic Stability in Preterm Neonates: A Randomized Controlled Trial. *Indian Pediatr* 2017; **54**(2): 112-5.

Boere I, Smit M, Roest AAW, Lopriore E, van Lith JMM, te Pas AB. Current practice of cord clamping in the Netherlands: a questionnaire study. *Neonatology* 2015; **107**(1): 50-5.

Sæther E. Re: Avnavling ved fødsel – praksis ved norske fødeinstitusjoner. *Tidsskr Nor Laegeforen* 2014; **134**: 692-.

Erlandsson K, Wells MB, Wagoro MC, et al. Implementing an internet-based capacity building program for interdisciplinary midwifery-lead teams in Ethiopia, Kenya Malawi and Somalia. *Sex Reprod Healthc* 2021; **30**: 100670.

Ramadhani FB, Liu Y, Lembuka MM. Knowledge and barriers on correct use of modified guidelines for active management of third stage of labour: a cross sectional survey of nurse-midwives at three referral hospitals in Dar es Salaam, Tanzania. *Afr Health Sci* 2020; **20**(4): 1908-17.

Aydogan Kirmizi D, Başer E, Demir Çaltekin M, Onat T, Kara M, Yalvac ES. Behaviors and Attitudes of Obstetricians in Turkey Related to Cord Clamping, Cord Milking, and Skin-To-Skin Contact. *Cureus* 2021; **13**(7): e16227.

Payne L, Walker KF, Mitchell EJ. Timing of umbilical cord clamping for preterm infants in low-and-middle-income countries: A survey of current practice. *Eur J Obstet Gynecol Reprod Biol* 2021; **264**: 15-20.

Cash transfer programmes to improve nutrition and address anaemia

Avitabile C, Cunha JM, Meilman Cohn R. The medium term impacts of cash and in-kind food transfers on learning. *Available at SSRN 3501896* 2019.

Gertler P. Do conditional cash transfers improve child health? Evidence from PROGRESA′ s control randomized experiment. *American economic review* 2004; **94**(2): 336-41.

Segura-Pérez S, Grajeda R, Pérez-Escamilla R. Conditional cash transfer programs and the health and nutrition of Latin American children. *Revista Panamericana de Salud Pública* 2016; **40**: 124-37.

Schady N. Cash transfers and anemia among women of reproductive age. *Economics Letters* 2012; **117**(3): 887-90.

Angrist N, Evans DK, Filmer D, Glennerster R, Rogers FH, Sabarwal S. How to improve education outcomes most efficiently. *A Comparison of* 2020; **150**.

Banerjee A, Andrab T, Banerji R, et al. 2023 Cost-effective Approaches to Improve Global Learning-What does Recent Evidence Tell Us are "Smart Buys" for Improving Learning in Low-and Middle-income Countries? *The Global Education Evidence Advisory Panel (GEEAP),* 2023.

Bergstrom K, Özler B. Improving the well-being of adolescent girls in developing countries. *The World Bank Research Observer* 2023; **38**(2): 179-212.

Evans DK, Yuan F. What we learn about girls' education from interventions that do not focus on girls. *The World Bank Economic Review* 2022; **36**(1): 244-67.

Inflammation, infections and anaemia

Anaemia of inflammation: the role of hepcidin

Layoun A, Samba-Mondonga M, Fragoso G, Calvé A, Santos MM. MyD88 adaptor protein is required for appropriate hepcidin induction in response to dietary iron overload in mice. *Frontiers in Physiology* 2018; **9**: 339243

Aschemeyer S, Qiao B, Stefanova D, et al. Structure-function analysis of ferroportin defines the binding site and an alternative mechanism of action of hepcidin. *Blood, The Journal of the American Society of Hematology* 2018; **131**(8): 899-910.

Anaemia and Malaria

World Health Organization. Strategy to respond to antimalarial drug resistance in Africa. Geneva: World Health Organization; 2022.

Burgmann H, Looareesuwan S, Kapiotis S, et al. Serum levels of erythropoietin in acute Plasmodium falciparum malaria. *The American journal of tropical medicine and hygiene* 1996; **54**(3): 280-3.

Douglas NM, Anstey NM, Buffet PA, et al. The anaemia of Plasmodium vivax malaria. *Malaria journal* 2012; **11**: 1-14.

Knüttgen H. The bone marrow of non-immune Europeans in acute malaria infection: a topical review. *Annals of Tropical Medicine & Parasitology* 1987; **81**(5): 567-76.

Phillips R, Looareesuwan S, Warrell D, et al. The importance of anaemia in cerebral and uncomplicated falciparum malaria: role of complications, dyserythropoiesis and iron sequestration. *QJM: An International Journal of Medicine* 1986; **58**(3-4): 305-23.

Price RN, Simpson JA, Nosten F, et al. Factors contributing to anemia after uncomplicated falciparum malaria. *The American journal of tropical medicine and hygiene* 2001; **65**(5): 614.

Vedovato M, Vitali EdP, Dapporto M, Salvatorelli G. Defective erythropoietin production in the anaemia of malaria. *Nephrology, dialysis, transplantation* 1999; **14**: 1043-4.

Aregawi MW, Ali AS, Al-Mafazy A-W, et al. Reductions in malaria and anaemia case and death burden at hospitals following scale-up of malaria control in Zanzibar, 1999-2008. *Malaria journal* 2011; **10**: 1-9.

Korenromp EL, Armstrong-Schellenberg JR, Williams BG, Nahlen BL, Snow RW. Impact of malaria control on childhood anaemia in Africa–a quantitative review. *Tropical medicine & international health* 2004; **9**(10): 1050-65.

Mathanga DP, Campbell CH, Eng JV, et al. Comparison of anaemia and parasitaemia as indicators of malaria control in household and EPI-health facility surveys in Malawi. *Malaria journal* 2010; **9**: 1-10.

Langford S, Douglas NM, Lampah DA, et al. Plasmodium malariae infection associated with a high burden of anemia: a hospital-based surveillance study. *PLoS neglected tropical diseases* 2015; **9**(12): e0004195.

Pava Z, Burdam FH, Handayuni I, et al. Submicroscopic and asymptomatic Plasmodium parasitaemia associated with significant risk of anaemia in Papua, Indonesia. *PloS one* 2016; **11**(10): e0165340.

Saute F, Menendez C, Mayor A, et al. Malaria in pregnancy in rural Mozambique: the role of parity, submicroscopic and multiple Plasmodium falciparum infections. *Tropical Medicine & International Health* 2002; **7**(1): 19-28.

van Eijk AM, Sutton PL, Ramanathapuram L, et al. The burden of submicroscopic and asymptomatic malaria in India revealed from epidemiology studies at three varied transmission sites in India. *Scientific reports* 2019; **9**(1): 17095.

de Mast Q, Syafruddin D, Keijmel S, et al. Increased serum hepcidin and alterations in blood iron parameters associated with asymptomatic P. falciparum and P. vivax malaria. *haematologica* 2010; **95**(7): 1068.

Cercamondi CI, Egli IM, Ahouandjinou E, et al. Afebrile Plasmodium falciparum parasitemia decreases absorption of fortification iron but does not affect systemic iron utilization: a double stable-isotope study in young Beninese women. *The American journal of clinical nutrition* 2010; **92**(6): 1385-92.

Glinz D, Hurrell RF, Righetti AA, et al. In Ivorian school-age children, infection with hookworm does not reduce dietary iron absorption or systemic iron utilization, whereas afebrile Plasmodium falciparum infection reduces iron absorption by half. *The American journal of clinical nutrition* 2015; **101**(3): 462-70.

Tuberculosis disease

Van Lettow M, West C, van Der Meer J, Wieringa F, Semba R. Low plasma selenium concentrations, high plasma human immunodeficiency virus load and high interleukin-6 concentrations are risk factors associated with anemia in adults presenting with pulmonary tuberculosis in Zomba district, Malawi. *European journal of clinical nutrition* 2005; **59**(4): 526-32.

Libregts SF, Gutiérrez L, de Bruin AM, et al. Chronic IFN-γ production in mice induces anemia by reducing erythrocyte life span and inhibiting erythropoiesis through an IRF-1/PU. 1 axis. *Blood, The Journal of the American Society of Hematology* 2011; **118**(9): 2578-88.

De Voss JJ, Rutter K, Schroeder BG, Barry III CE. Iron acquisition and metabolism by mycobacteria. *Journal of bacteriology* 1999; **181**(15): 4443-51.

Schaible UE, Collins HL, Priem F, Kaufmann SH. Correction of the iron overload defect in β-2-microglobulin knockout mice by lactoferrin abolishes their increased susceptibility to tuberculosis. *The Journal of experimental medicine* 2002; **196**(11): 1507-13.

Devi U, Rao CM, Srivastava VK, Rath PK, Das BS. Effect of iron supplementation on mild to moderate anaemia in pulmonary tuberculosis. *British Journal of Nutrition* 2003; **90**(3): 541-50.

HIV

Clark T, Mmiro F, Ndugwa C, et al. Risk factors and cumulative incidence of anaemia among human immunodeficiency virus-infected children in Uganda. *Annals of tropical paediatrics* 2002; **22**(1): 11-7.

Ellaurie M, Burns ER, Rubinstein A. Hematologic manifestations in pediatric HIV infection: severe anemia as a prognostic factor. *Journal of Pediatric Hematology/Oncology* 1990; **12**(4): 449-53.

Redig AJ, Berliner N. Pathogenesis and clinical implications of HIV-related anemia in 2013. *Hematology 2013, the American Society of Hematology Education Program Book* 2013; **2013**(1): 377-81.

Parinitha SS, Kulkarni MH. Haematological changes in HIV infection with correlation to CD4 cell count. *The Australasian Medical Journal* 2012; **5**: 157-62.

Anaemia due to soil-transmitted helminths and schistosomiasis

Buonfrate D, Requena-Mendez A, Angheben A, et al. Accuracy of molecular biology techniques for the diagnosis of Strongyloides stercoralis infection—a systematic review and meta-analysis. *PLoS neglected tropical diseases* 2018; **12**(2): e0006229.

Dassah SD, Nyaah KE, Senoo DKJ, et al. Co-infection of Plasmodium falciparum and Schistosoma mansoni is associated with anaemia. *Malaria Journal* 2023; **22**(1): 272.

Hotez PJ, Alvarado M, Basáñez M-G, et al. The global burden of disease study 2010: interpretation and implications for the neglected tropical diseases. *PLoS neglected tropical diseases* 2014; **8**(7): e2865.

Welch VA, Ghogomu E, Hossain A, et al. Mass deworming to improve developmental health and wellbeing of children in low-income and middle-income countries: a systematic review and network meta-analysis. *The Lancet Global Health* 2017; **5**(1): e40-e50.

The role of gut health in anaemia

Arthur CM, Nalbant D, Feldman HA, et al. Anemia induces gut inflammation and injury in an animal model of preterm infants. *Transfusion* 2019; **59**(4): 1233-45.

Jaeggi T, Kortman GA, Moretti D, et al. Iron fortification adversely affects the gut microbiome, increases pathogen abundance and induces intestinal inflammation in Kenyan infants. *Gut* 2015; **64**(5): 731-42.

Paganini D, Uyoga MA, Zimmermann MB. Iron fortification of foods for infants and children in low-income countries: effects on the gut microbiome, gut inflammation, and diarrhea. *Nutrients* 2016; **8**(8): 494.

Larsen DA, Grisham T, Slawsky E, Narine L. An individual-level meta-analysis assessing the impact of community-level sanitation access on child stunting, anemia, and diarrhea: Evidence from DHS and MICS surveys. *PLoS Negl Trop Dis* 2017; **11**(6): e0005591.

Stewart CP, Dewey KG, Lin A, et al. Effects of lipid-based nutrient supplements and infant and young child feeding counseling with or without improved water, sanitation, and hygiene (WASH) on anemia and micronutrient status: results from 2 cluster-randomized trials in Kenya and Bangladesh. *The American Journal of Clinical Nutrition* 2019; **109**(1): 148-64.

Stoler J, Guzmán DB, Adams EA. Measuring transformative WASH: A new paradigm for evaluating water, sanitation, and hygiene interventions. *Wiley Interdisciplinary Reviews: Water* 2023; **10**(5): e1674.

Anaemia in older people

McQuilten ZK, Thao LTP, Pasricha S-R, et al. Effect of Low-Dose Aspirin Versus Placebo on Incidence of Anemia in the Elderly. *Annals of Internal Medicine* 2023; **176**(7): 913-21.

Environmental considerations for anaemia

Air pollution

Honda T, Pun VC, Manjourides J, Suh H. Anemia prevalence and hemoglobin levels are associated with long-term exposure to air pollution in an older population. *Environment International* 2017; **101**: 125-32.

Kwag Y, Ye S, Oh J, et al. Direct and Indirect Effects of Indoor Particulate Matter on Blood Indicators Related to Anemia. *International Journal of Environmental Research and Public Health*, 2021. (accessed).

Deng Y, Steenland K, Sinharoy SS, et al. Association of household air pollution exposure and anemia among pregnant women: Analysis of baseline data from 'Household Air Pollution Intervention Network (HAPIN)' trial. *Environment International* 2024; **190**: 108815.

Ghio AJ, Soukup JM, Dailey LA, Madden MC. Air pollutants disrupt iron homeostasis to impact oxidant generation, biological effects, and tissue injury. *Free radical biology & medicine* 2020; **151**(1873-4596 (Electronic)): 38–55.

Brody JS, Coburn RF. Carbon Monoxide-Induced Arterial Hypoxemia. *Science* 1969; **164**(3885): 1297-8.

Climate change

Owino V, Kumwenda C, Ekesa B, et al. The impact of climate change on food systems, diet quality, nutrition, and health outcomes: A narrative review. *Frontiers in Climate* 2022; **4**.

Blood loss and anaemia in women of reproductive age

Heavy menstrual bleeding

DeLoughery TG. Iron Deficiency Anemia. In: America MCoN, editor.; 2017. p. 319-32.

Munro MG. Heavy menstrual bleeding, iron deficiency, and iron deficiency anemia: Framing the issue. *International Journal of Gynecology & Obstetrics* 2023; **162**: 7-13.

Interventions to reduce heavy menstrual blood loss

Jensen JT, Parke S, Mellinger U, Machlitt A, Fraser IS. Effective treatment of heavy menstrual bleeding with estradiol valerate and dienogest: a randomized controlled trial. *Obstetrics & Gynecology* 2011; **117**(4): 777-87.

Sayed GH, Zakherah MS, El-Nashar SA, Shaaban MM. A randomized clinical trial of a levonorgestrel-releasing intrauterine system and a low-dose combined oral contraceptive for fibroid-related menorrhagia. *International Journal of Gynecology & Obstetrics* 2011; **112**(2): 126-30.

Shabaan MM, Zakherah MS, El-Nashar SA, Sayed GH. Levonorgestrel-releasing intrauterine system compared to low dose combined oral contraceptive pills for idiopathic menorrhagia: a randomized clinical trial. *Contraception* 2011; **83**(1): 48-54.

Postpartum haemorrhage

Glonnegger H, Glenzer MM, Lancaster L, Barnes RF, von Drygalski A. Prepartum Anemia and Risk of Postpartum Hemorrhage: A Meta-Analysis and Brief Review. *Clinical and Applied Thrombosis/Hemostasis* 2023; **29**: 10760296231214536.

Omotayo MO, Abioye AI, Kuyebi M, Eke AC. Prenatal anemia and postpartum hemorrhage risk: A systematic review and meta-analysis. *Journal of Obstetrics and Gynaecology Research* 2021; **47**(8): 2565-76.

Bambo GM, Kebede SS, Sitotaw C, Shiferaw E, Melku M. Postpartum anemia and its determinant factors among postnatal women in two selected health institutes in Gondar, Northwest Ethiopia: A facility-based, cross-sectional study. *Frontiers in Medicine* 2023; **10**.

Section 3: Improving anaemia control programs: implementation and governance.

Current state of anaemia governance and implementation

Hamdan M, Brabin B, Bates I. Implications of inconsistent anaemia policies for children and adolescents in Africa. *Public Health Nutrition*. 2014;**17**(11):2587-94.

Kentikelenis A, Rochford C. Power asymmetries in global governance for health: a conceptual framework for analyzing the political-economic determinants of health inequities. *Globalization and Health*. 2019;**15**(1):70.

Core Principle 1: leverage existing data and collecting new data

Morrison J, Giri R, Arjyal A, et al. Addressing anaemia in pregnancy in rural plains Nepal: A qualitative, formative study. *Matern Child Nutr* 2021; **17 Suppl 1**(Suppl 1): e13170.

Core Principle 2: catalyse multisectoral governance and engagement

Sarkar D, Murphy H, Fisseha T, et al. Understanding the process of strengthening multi-sectoral efforts for anemia reduction: Qualitative findings from Sierra Leone and Uganda. *Int J Health Plann Manage* 2018; **33**(4): 1024-44.

World Health Organization. Promoting Health in All Policies and intersectoral action capacities. Available from: https://www.who.int/activities/promoting-health-in-all-policies-and-intersectoral-action-capacities.

Global governance for anaemia

Current global governance architecture (and Table 5)

Galea LA, Parekh RS. Ending the neglect of women's health in research. *BMJ* 2023; **381**: 1303.

Every Woman Every Child. The Global Strategy for Women’s, Children’s and Adolescents’ Health (2016–30). 2015.

World Health Organization. The Global Action Plan for Healthy Lives and Well-being for All (SDG3 GAP) 2019. Available from: https://www.who.int/initiatives/sdg3-global-action-plan.

Funding research on women’s health. *Nature Reviews Bioengineering* 2024; **2**(10): 797-8.

Smith K. Women’s health research lacks funding – these charts show how. *Nature* 2023.

Wilson SE, Rogers LM, Garcia-Casal MN, et al. Comprehensive framework for integrated action on the prevention, diagnosis, and management of anemia: An introduction. *Annals of the New York Academy of Sciences* 2023; **1524**(1): 5-9.

Gore R, Parker R. Analysing power and politics in health policies and systems. *Global Public Health* 2019; **14**(4): 481-8.

National government-led anaemia strategies

Building and sustaining political commitment for anaemia reduction

Heaver RA. Good work -- but not enough of it : a review of the World Bank's experience in nutrition. Washington, D.C: World Bank Group, 2006.

Natalicchio M, Garrett J, Mulder-Sibanda M, Ndegwa S, Voorbraak D. Carrots and sticks : the political economy of nutrition policy reforms. Washington, D.C: World Bank Group, 2009.

Acosta AM. Analysing Success in the Fight against Malnutrition in Peru. *IDS Working Papers* 2011; **2011**(367): 2-49.

Pelletier DL, P. M, Ngo T, Frongillo EA, Frongillo D. The nutrition policy process: the role of strategic capacity in advancing national nutrition agendas. *Food Nutr Bull* 2011; **32**(0379-5721 (Print)): S59-69.

Mobilising domestic resources and financial flows

World Bank. Optima Nutrition Learning Tool 2019. Available from: https://www.worldbank.org/en/topic/health/brief/optima-nutrition-learning-tool.

Section 4: A better way forward: redefining future anaemia reduction targets

Mannar V, Micha R, Allemandi L, et al. 2020 Global nutrition report: action on equity to end malnutrition. Bristol, U.K.: Development Initiatives Poverty Research, 2020.

International development goals

Gupta S, Hammond B, Leete R, Swanson E. Progress Toward the International Development Goals, 2000.

The purpose of international target-setting

Gurin J, Manley L, Ariss A. Sustainable Development Goals and Open Data. 2015. https://blogs.worldbank.org/en/digital-development/sustainable-development-goals-and-open-data.

Reddy SG, Pogge T. How Not to Count the Poor. Debates on the Measurement of Global Poverty: Oxford University Press; 2010. p. 0.

Global anaemia targets

Zaninetti C, Klersy C, Scavariello C, Bastia R, Balduini CL, Invernizzi R. Prevalence of anemia in hospitalized internal medicine patients: Correlations with comorbidities and length of hospital stay. *European Journal of Internal Medicine* 2018; **51**: 11-7.

Mason J, Martorell R, Saldanha L, Shrimpton R. Reduction of anaemia. *The Lancet Global Health* 2013; **1**(1): e4-e6.

Focus on women of reproductive age

Weber AM, Cislaghi B, Meausoone V, et al. Gender norms and health: insights from global survey data. *The Lancet* 2019; **393**(10189): 2455-68.

A proposal for evidence-based target setting

Walker N, Tam Y, Friberg IK. Overview of the Lives Saved Tool (LiST). *BMC Public Health* 2013; **13 Suppl 3**(Suppl 3): S1.

Jamison DT, Alwan A, Mock CN, et al. Universal health coverage and intersectoral action for health: key messages from Disease Control Priorities, 3rd edition. *The Lancet* 2018; **391**(10125): 1108-20.

Gelband H, Sankaranarayanan R, Gauvreau CL, et al. Costs, affordability, and feasibility of an essential package of cancer control interventions in low-income and middle-income countries: key messages from Disease Control Priorities, 3rd edition. *The Lancet* 2016; **387**(10033): 2133-44.

Challenges related to future target setting

Carvalho N, Sousa TV, Mizdrak A, Jones A, Wilson N, Blakely T. Comparing health gains, costs and cost-effectiveness of 100s of interventions in Australia and New Zealand: an online interactive league table. *Population Health Metrics*. 2022;**20**(1):17.

Conclusions

Andrews N, Khalema NE, Assié-Lumumba NDT. Millennium Development Goals (MDGs) in Retrospect Africa's Development Beyond 2015; 2015.

Department of Economic and Social Affairs (United Nations). Millennium Development Goals: 2015 Progress Chart: United Nations, 2015.

United Nations Development Programme. Destination 2030 Accelerating progress on the Sustainable Development Goals. 2024. https://www.undp.org/destination-2030-accelerating-progress-sustainable-development-goals2024).

References for Appendix:

1. World Health Organization. Guideline on haemoglobin cutoffs to define anaemia in individuals and populations. Geneva, 2024.

2. Centers for Disease Control Prevention. National health and nutrition examination survey (NHANES): MEC Laboratory Procedures Manual. Atlanta: Centers for Disease Control and Prevention; 2020.

3. Tremblay JC, Ainslie PN. Global and country-level estimates of human population at high altitude. *Proceedings of the National Academy of Sciences* 2021; **118**(1091-6490 (Electronic)).
